# Image Contrast, Image Pre-Processing, and T_1_ Mapping Affect MRI Radiomic Feature Repeatability in Patients with Colorectal Cancer Liver Metastases

**DOI:** 10.3390/cancers13020240

**Published:** 2021-01-11

**Authors:** Damien J. McHugh, Nuria Porta, Ross A. Little, Susan Cheung, Yvonne Watson, Geoff J. M. Parker, Gordon C. Jayson, James P. B. O’Connor

**Affiliations:** 1 Division of Cancer Sciences, The University of Manchester, Manchester M13 9PL, UK; damien.mchugh@manchester.ac.uk (D.J.M.); ross.little@manchester.ac.uk (R.A.L.); susan.cheung@manchester.ac.uk (S.C.); yvonwatson2@gmail.com (Y.W.); gordon.jayson@manchester.ac.uk (G.C.J.); 2 Quantitative Biomedical Imaging Laboratory, The University of Manchester, Manchester M13 9PL, UK; 3 Clinical Trials and Statistics Unit, Institute of Cancer Research, London SW3 6JB, UK; nuria.porta@icr.ac.uk; 4 Centre for Medical Image Computing, University College London, London WC1V 6LJ, UK; geoff.parker@ucl.ac.uk; 5 Bioxydyn Ltd., Manchester M15 6SZ, UK; 6 Department of Medical Oncology, The Christie Hospital, Manchester M20 4BX, UK; 7 Department of Radiology, The Christie Hospital, Manchester M20 4BX, UK; 8 Division of Radiotherapy and Imaging, Institute of Cancer Research, London SW3 6JB, UK

**Keywords:** radiomics, MRI, repeatability, repeatability coefficient, intraclass correlation coefficient, liver metastases

## Abstract

**Simple Summary:**

Medical images are data. They contain more information than is routinely identified by radiologists reading scans. Many scientists are investigating if extracting shape and grey-scale features from images can predict which oncology patients will respond to therapy. This approach, termed ‘radiomics’, must be validated before being ready for clinical use. One step is to determine measurement repeatability to ensure that radiomic features are robust, and that changes in features reflect genuine changes in tumours. In this study patients had two repeated sets of magnetic resonance imaging scans. We found that radiomic feature repeatability varied depending on scan acquisition parameters and the use of an administered contrast agent. We also compared how different repeatability assessment methods can best reveal these findings. We conclude that measuring radiomic feature repeatability is essential, but is also complex and prone to pitfalls. Overall, our study provides several insights into how radiomic feature repeatability is best assessed.

**Abstract:**

Imaging biomarkers require technical, biological, and clinical validation to be translated into robust tools in research or clinical settings. This study contributes to the technical validation of radiomic features from magnetic resonance imaging (MRI) by evaluating the repeatability of features from four MR sequences: pre-contrast T${}_{1}$- and T${}_{2}$-weighted images, pre-contrast quantitative T${}_{1}$ maps (qT${}_{1}$), and contrast-enhanced T${}_{1}$-weighted images. Fifty-one patients with colorectal cancer liver metastases were scanned twice, up to 7 days apart. Repeatability was quantified using the intraclass correlation coefficient (ICC) and repeatability coefficient (RC), and the impact of non-Gaussian feature distributions and image normalisation was evaluated. Most radiomic features had non-Gaussian distributions, but Box–Cox transformations enabled ICCs and RCs to be calculated appropriately for an average of 97% of features across sequences. ICCs ranged from 0.30 to 0.99, with volume and other shape features tending to be most repeatable; volume ICC > 0.98 for all sequences. 19% of features from non-normalised images exhibited significantly different ICCs in pair-wise sequence comparisons. Normalisation tended to increase ICCs for pre-contrast T${}_{1}$- and T${}_{2}$-weighted images, and decrease ICCs for qT${}_{1}$ maps. RCs tended to vary more between sequences than ICCs, showing that evaluations of feature performance depend on the chosen metric. This work suggests that feature-specific repeatability, from specific combinations of MR sequence and pre-processing steps, should be evaluated to select robust radiomic features as biomarkers in specific studies. In addition, as different repeatability metrics can provide different insights into a specific feature, consideration of the appropriate metric should be taken in a study-specific context.

## 1. Introduction

Imaging underpins much of the current management of patients with cancer, through diagnosis, staging and monitoring response to therapy. There is considerable current interest in evaluating if high throughput analysis of medical images—in an approach termed ‘radiomics’—can further extend the role of imaging by producing signatures that are prognostic or are predictive of clinical outcome [1,2,3].

For radiomics to yield robust imaging biomarkers, technical, biological, and clinical validation are required [4,5]. Such validation is a multi-step process, in which various aspects of a proposed biomarker’s performance are evaluated. Technical validation requires the evaluation of biomarker accuracy, repeatability, reproducibility, and availability, while biological and clinical validation require an understanding of how features relate to underlying biology, and how they relate to outcome, respectively. There has been a substantial amount of research into how radiomic features relate to tumour biology and outcome. Many studies have focused on finding a statistical association between either one radiomic feature, or several features combined into a ‘signature’, and an underlying biological feature or clinical outcome [6,7]. For example, computed tomography (CT) radiomic features from the Gray-Level Run Length Matrix in the tumour and peripheral ring, along with the minimum value in the tumour, have been associated with CD8 cell infiltration across a range of tumour types [8], and CT features related to tumour heterogeneity and compactness/sphericity have shown an association with survival in lung and head and neck cancer [2].

In terms of technical validation, measuring repeatability in single centres and reproducibility across multiple centres is crucial, and provides an important step in developing metrology standards for quantitative imaging biomarkers in general [9], including radiomic features [10]. While a number of studies have assessed the repeatability and/or reproducibility of CT-derived cancer radiomic features [11], there are fewer studies investigating the repeatability of MR-derived radiomic features [12,13,14,15,16,17,18,19,20,21]. These studies are limited by small patient numbers (≤17, with the exception of Kickingereder et al., [13] and Merisaari et al. [20], with 55 and 112 patients, respectively), and the use of only one or two MR sequences (except in [13] where three were used). Furthermore, given the difficulty in directly comparing MR signal intensities from different scans, the effect of image normalisation on repeatability needs to be considered for different MR sequences, as does the validity of assumptions underlying the statistical analysis of repeatability. Finally, the impact of gadolinium-based contrast agents on the repeatability of features from T${}_{1}$-weighted images, and the repeatability of features from T${}_{1}$ maps, is yet to be evaluated in tumours.

This study aimed to address these knowledge gaps. To achieve this we sought to provide a comprehensive evaluation of the repeatability of MRI derived radiomic features in patients with liver metastases from colorectal cancer, which is an emerging clinical site of interest [22].

## 2. Materials and Methods

### 2.1. Image Acquisition and ROI Definition

**Figure 1:**
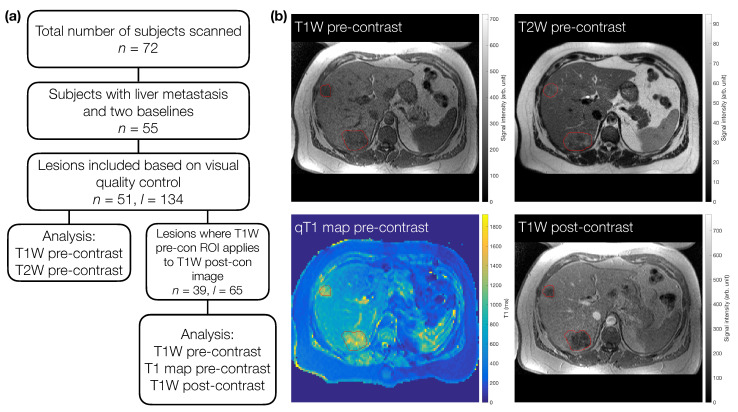
(**a**) Flowchart for subject and lesion selection. For T${}_{1}$W and T${}_{2}$W pre-contrast images, a total of 51 subjects (*n*) with 134 lesions (*l*) were included. Sixty-five of these lesions were also analysed on T${}_{1}$W post-contrast images and pre-contrast quantitative T${}_{1}$ maps. For direct comparison in terms of number of lesions included, the T${}_{1}$W pre-contrast images from this subset were re-analysed in 39 patients. (**b**) Example images for one subject with two metastases (red outlines).

We examined pre-treatment MRI scan data in patients recruited for a clinical trial (EudraCT number 2009-011377-33) [23]. All patients gave written informed consent. The study was conducted in accordance with the Declaration of Helsinki, and the study received institutional board approval (North West–Greater Manchester Central Research Ethics Committee, REC 09/H1008/99). All included patients were aged 18 years or over, had primary colorectal cancer, with at least one liver metastasis measuring >2.5 cm in maximum dimension on their screening CT scan, had a performance status of 0–2, and had two pre-treatment scans (median time between scans =4 days, range = 2–7 days) (Figure 1a). Given the retrospective nature of this study, the available data determined the sample size, rather than a formal power calculation.

MRI acquisition and analysis was performed to Good Clinical Practice (GCP) standards. Data were acquired on a 1.5 T Philips Achieva scanner (Philips Healthcare, Best, The Netherlands). All imaging sequences were acquired without breath-holds or gating, with 25 axial slices, either 4 or 8 mm thick. Individual sequence parameters were:

- Multislice 2D T${}_{1}$-weighted turbo field-echo sequence prior to contrast agent administration (flip angle (FA) = $15^{\circ }$, repetition time (TR) = 10 ms, echo time (TE) = 4.60 ms, field of view (FoV) = 375 mm × 264 mm, acquired in-plane resolution = 1.46 mm × 2.09 mm, reconstructed in-plane resolution = 1.46 mm × 1.46 mm, 25 slices); hereafter termed T${}_{1}$W pre-contrast.
- Multislice 2D T${}_{2}$-weighted turbo spin-echo sequence (FA = $90^{\circ }$, TR = 541 ms, TE = 80 ms, FoV = 375 mm × 264 mm, acquired in-plane resolution = 1.46 mm × 1.84 mm, reconstructed in-plane resolution = 1.46 mm × 1.46 mm, 25 slices); hereafter termed T${}_{2}$W pre-contrast.
- Three 3D spoiled gradient-echo sequences (FAs = $2^{\circ },10^{\circ },20^{\circ }$, TR = 4 ms, TE = 0.82 ms, FoV = 375 mm × 375 mm, acquired in-plane resolution = 2.93 mm × 3.71 mm, reconstructed in-plane resolution = 2.93 mm × 2.93 mm, 25 slices). T${}_{1}$ was quantified by fitting the spoiled gradient-echo equation [24] to the signal intensities, *S*, in the three flip angle images: $S\left(\alpha \right)=M_{0}\sin\alpha \left(1-E\right)/\left(1-E\cos\alpha \right)$, where $M_{0}$ is a factor proportional to proton density, α is the flip angle, and $E=\exp\left(-\mathrm{TR}/T_{1}\right)$. Fitting was performed on a voxelwise basis using a Levenberg-Marquardt algorithm, and the resulting T${}_{1}$ maps are hereafter termed qT${}_{1}$ maps.
- Multislice 2D T${}_{1}$-weighted turbo field-echo sequence acquired 5 min after contrast agent administration (FA = $15^{\circ }$, TR = 10 ms, TE = 4.60 ms, FoV 375 mm × 264 mm, acquired in-plane resolution = 1.46 mm × 2.09 mm, reconstructed in-plane resolution = 1.46 mm × 1.46 mm, 25 slices). 0.1 mmol/kg of gadoterate meglumine contrast agent (Dotarem, Guebert, France) was administered intravenously at a rate of 3 ml/s, using a Medrad Spectris power injector (Bayer, Reading, UK); hereafter termed T${}_{1}$W post-contrast.

Regions of interest (ROIs) were defined manually using Java Image software (JIM version 6.0_16, Xinpase Systems Ltd, UK) by a radiologist (J.P.B.O’C; 16 years of experience). In each case an ROI was drawn on the T${}_{1}$W pre-contrast images, and again on the T${}_{2}$W pre-contrast images. Both pre-treatment scans were annotated together. Up to five target lesions were identified for each patient. Next, the qT${}_{1}$ maps and the T${}_{1}$W post-contrast images were inspected along with the ROIs drawn on the accompanying T${}_{1}$W pre-contrast image to determine if the ROIs provided accurate delineation of each target lesion on these sequences. If the ROI was not deemed accurate for the qT${}_{1}$ maps and the T${}_{1}$W post-contrast images then data were excluded for those target lesions. Lesions included for the qT${}_{1}$ maps and T${}_{1}$W post-contrast images were therefore a subset of those for the T${}_{1}$W and T${}_{2}$W pre-contrast images; the T${}_{1}$W pre-contrast images from this subset were treated as another dataset, to allow a direct comparison (in terms of lesion numbers) with qT${}_{1}$ map and T${}_{1}$W post-contrast data (Figure 1a). The qT${}_{1}$ map masks were created based on the ROI defined on the higher-resolution T${}_{1}$W pre-contrast images. Due to the different resolutions of the weighted and quantitative images, these qT${}_{1}$ map masks were subsequently up-sampled for application to the subset of T${}_{1}$W pre-contrast images, to ensure directly comparable masks were used when comparing qT${}_{1}$ maps to the T${}_{1}$W pre-contrast subset (Supplementary Information Figure S1). Each patient therefore had data for up to four sequences for two separate scan sessions. Example ROIs for one patient are shown in Figure 1b.

### 2.2. Radiomic Feature Extraction

For all ROI in all imaging sequences, radiomic features were extracted using PyRadiomics, version 2.2.0.post41+gc46ed88 [25]. PyRadiomics was chosen as it is open source, which facilitates reproducible research, and is largely ISBI-compliant (for details of differences see https://pyradiomics.readthedocs.io/en/latest/faq.html), which can aid comparison with studies using other ISBI-compliant platforms [26]. In total, 105 features were extracted from every lesion, with each feature belonging to one of the seven standard classes in PyRadiomics: Shape, First Order, Gray Level Co-occurrence Matrix (GLCM), Gray Level Run Length Matrix (GLRLM), Gray Level Size Zone Matrix (GLSZM), Gray Level Dependence Matrix (GLDM), and Neighbouring Gray Tone Difference Matrix (NGTDM). Descriptions and formulae for all features can be found at https://pyradiomics.readthedocs.io/en/latest/features.html. 3D feature extraction was performed for images without normalisation, and for images normalised to have a mean of 0 and standard deviation of 100, using PyRadiomics’ standard linear normalisation. In all cases, a bin width of 5 was used, and no filters were applied to images before feature extraction. For T${}_{1}$W pre-contrast, T${}_{2}$W pre-contrast, qT${}_{1}$ pre-contrast, and T${}_{1}$W post-contrast, the median number of bins over all lesions and visits was 60, 13, 310, and 110 (non-normalised) and 29, 42, 45, and 38 (normalised). YAML parameter files and analysis code used in this study can be found at https://gitlab.com/manchester_qbi/manchester_qbi_public/radiomics_repeatability.

### 2.3. Repeatability Analysis and Statistical Comparison

The repeatability of radiomic features was evaluated using the intraclass correlation coefficient (ICC) [27,28,29] and the repeatability coefficient (RC) [30]. ICC is a measure of within-subjects consistency relative to the total variability observed in the population, and it can be estimated in different ways, depending on the underlying statistical model that best captures the data structure under study; indeed, a feature’s ICC depends on the variability of the feature across the cohort studied [28]. Here, ICC(1,1) [28] was judged to be the most suitable (see Supplementary Information), with point estimates and 95% CIs calculated as described by McGraw and Wong [27]. RC point estimates and 95% CIs were calculated as described by Barnhart and Barboriak [30]. RC is proportional to the within-subject standard deviation, and for a given feature the difference between repeat measurements is expected to fall within −RC and +RC, for 95% of patients. As such, RC is a useful metric for determining significant changes in a feature over time, for example in assessing treatment-induced changes relative to baseline. In contrast to ICCs, RCs depend on the magnitude and unit of the underlying feature, and themselves have the unit that the feature is measured in.

The models underlying ICC and RC calculations assume the feature follows a Gaussian distribution [29,31], and deviations from this assumption have been reported to impact ICC point estimates and confidence intervals (CIs) [32]. The assumption of Gaussian feature distributions was tested using the Shapiro–Wilk test for all radiomic features extracted from all MR sequences (for non-normalised and normalised images). Feature distributions were judged to be non-Gaussian based on a Bonferroni-corrected p<0.05/105 threshold in the Shapiro–Wilk test (given 105 features per dataset). For non-Gaussian features, the optimal λ parameter to use in a Box–Cox transformation was found, such that the original feature distribution, *x*, could be transformed to a new distribution, *y*, which was consistent with a Gaussian distribution, according to $y=(x^{\lambda }-1)/\lambda$, if λ≠0; y=log(x), if λ=0 [33]. Shapiro–Wilk tests and Box–Cox transformations were carried out using the *stats* subpackage of the SciPy library in Python [34].

Note that we refer to ‘Gaussian distributions’ of the radiomics features rather than ‘normal distributions’, to avoid confusion with the term ‘image normalisation’, which refers to the transformation applied to image signal intensities prior to feature extraction. To assess the impact of Box–Cox transformations and image normalisation across images with different magnitudes and units, only ICCs were used, as RCs depend on the magnitude and unit of the underlying feature. As such, there were 16 datasets from which ICCs could be obtained: 4 MR sequences, each without and with image normalisation, and each without and with applying Box–Cox transformations to feature distributions.

To compare repeatability across different sequences, both ICC and RC were estimated, but for RC, only normalised datasets were used (where signal intensities become dimensionless) and were only estimated on the subset of features where the application of the Box–Cox transformation was consistent for all sequences (that is, for features where the transformation either was applied for all sequences, or was not applied for any sequence, to ensure comparable feature magnitudes). It should be noted that for features where the Box–Cox transformation was used, the direct comparison of RCs across sequences is hampered due to the different optimal λ values for the different sequences, as λ influences the feature magnitudes. This may potentially confound interpretability when comparing RCs from Box–Cox transformed features.

ICCs were formally compared using Fisher’s *Z*-test [35], which involves applying a transformation to the ICCs; note that this is independent of the Box–Cox transformations described above. The test allowed ICCs to be compared between features without and with Box–Cox transformations (e.g., T${}_{1}$W pre-contrast without Box–Cox transformations vs. T${}_{1}$W pre-contrast with Box–Cox transformations), between features from different MR sequences (e.g., T${}_{1}$W pre-contrast vs. T${}_{2}$W pre-contrast), and between features from non-normalised and normalised images (e.g., T${}_{1}$W pre-contrast without normalisation vs. T${}_{1}$W pre-contrast with normalisation). In these comparisons, ICCs were taken to be significantly different based on a Bonferroni-corrected p<0.05/105 threshold. RCs were descriptively compared across sequences, and with ICCs, on a per-feature basis.

## 3. Results

### 3.1. Effect of Box–Cox Transformations

**Figure 2:**
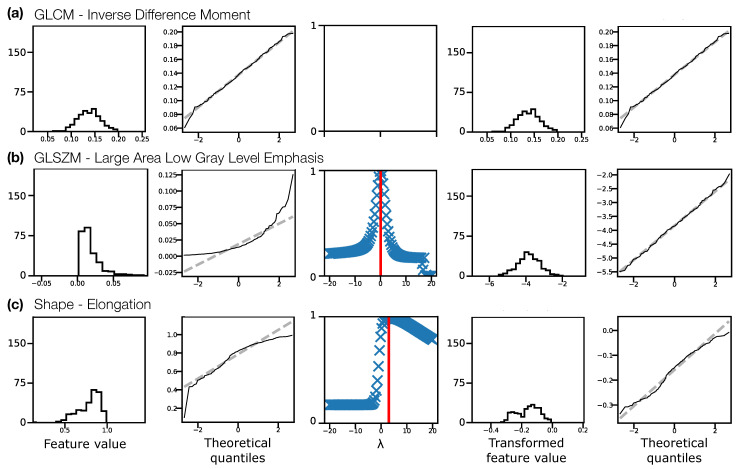
Feature distributions and transformations for three example features, (**a**) Inverse Difference Moment, (**b**) Large Area Low Gray Level Emphasis, (**c**) Elongation, from non-normalised T${}_{1}$W pre-contrast images, pooling feature values from both visits for all subjects. Feature distributions and Q-Q plots for the original features are shown in the first and second columns, respectively. The third column shows the Box–Cox normality plots, with red vertical lines indicating the optimal λ to use to transform the distributions. Feature distributions and Q-Q plots for the transformed features are shown in the fourth and fifth columns, respectively. In (**a**) the original distribution is consistent with a Gaussian distribution (Shapiro–Wilk test), and does not require a transformation. In (**b**) the original distribution is not consistent with a Gaussian distribution, and the transformation corrects this. In (**c**) the original distribution is not consistent with a Gaussian distribution, but the transformed distribution is still not Gaussian, though the Q-Q plots suggest the transformed distribution is closer to Gaussian than the original. Y-axis labels are omitted for clarity, but are: Counts, Sample quantiles, Correlation coefficient, Counts, Sample quantiles, for each column, respectively.

Figure 2 illustrates the Box–Cox transformation procedure for three example features from non-normalised T${}_{1}$W pre-contrast images. The chosen features, GLCM Inverse Difference Moment, GLSZM Large Area Low Gray Level Emphasis, and Elongation reflect three scenarios in terms of feature distributions: (a) the original distribution is Gaussian, and does not need transforming before ICC calculation; (b) the original distribution is not Gaussian, but becomes Gaussian after a Box–Cox transformation; and (c) the original distribution is not Gaussian, but is still not Gaussian after a Box–Cox transformation. In case (c), although the feature still fails the Shapiro–Wilk test, the quantile-quantile (Q-Q) plot indicates that the transformation does help to make the distribution more consistent with a Gaussian. Across all sequences, with and without normalisation, the majority of features reflected scenario (b), where the transformation corrected a previously non-Gaussian distribution (Table 1). With this procedure, Gaussian distributions could be obtained for >93% of features for all datasets. For features where the applied transformation still did not yield a Gaussian distribution, qualitative assessment of Q-Q plots suggested some improvement, so these transformed features were still used. Supplementary Information Figures S2–S5 show feature histograms, Box–Cox transformations, and Q-Q plots for all features, for all sequences.

**Figure 3:**
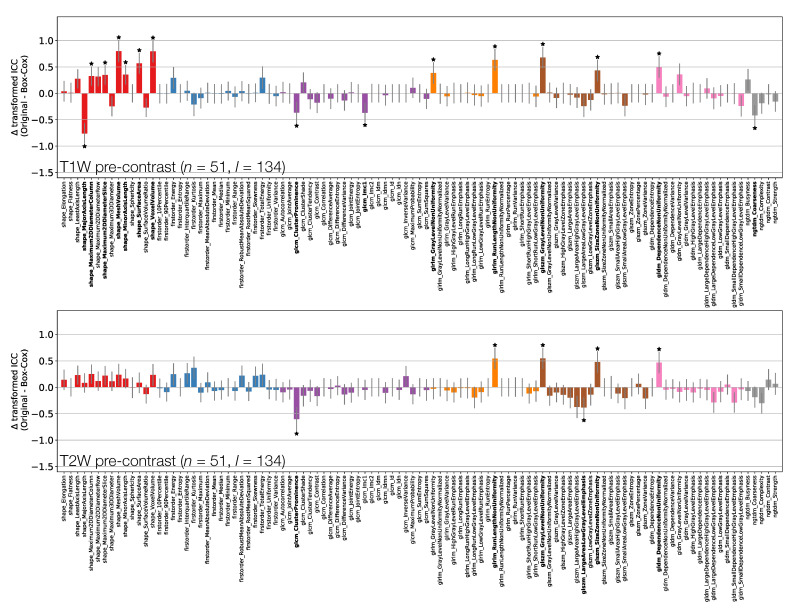
Effect of applying Box–Cox transformations to feature distributions, for T${}_{1}$W (top) and T${}_{2}$W (bottom) pre-contrast images. Bars represent the difference in ICC point estimates (after applying Fisher’s *Z*-transformation), and error bars represent 95% CIs. Features are colour coded according to their class: Shape (red), First Order (blue), GLCM (purple), GLRLM (orange), GLSZM (brown), GLDM (pink), and NGTDM (grey). Black stars and bold fonts indicate features where ICCs from original and Box–Cox transformed data are significantly different.

Figure 3 shows the effect of the Box–Cox transformations on calculated ICCs, for T${}_{1}$W and T${}_{2}$W pre-contrast non-normalised images (Supplementary Information Figure S6 shows equivalent plots for qT${}_{1}$ maps and T${}_{1}$W post-contrast non-normalised images). Note that these figures do not directly plot the ICC differences, but rather the difference in ICCs after applying Fisher’s *Z*-transformation, and the error bars show the 95% CI on this difference. Note also that not all features where the 95% CIs do not contain zero are marked as significant due to the use of Bonferroni correction.

Depending on the feature, ICC point estimates could increase or decrease due to the transformation, though differences tended to be relatively small, and significant differences were observed for a minority of features. This suggests that while ICC calculations assume features follow a Gaussian distribution, there is a degree of robustness against cases where this assumption is invalid. While the application of the transformations does not have a dramatic effect on ICCs, it does help ensure the validity of methodological assumptions, and it can significantly affect the repeatability of some features. As such, ICCs from Box–Cox transformed features were used throughout the rest of the analysis.

### 3.2. ICC and RC Overview

Features exhibited a wide range of repeatabilities, with ICCs ranging from 0.30 (GLSZM Small Area Emphasis for T${}_{2}$W images without normalisation) to 0.99 (Voxel Volume for T${}_{1}$W post-contrast images). For all sequences, Voxel Volume and Mesh Volume were the features with the two highest ICCs (>0.98), indicating that tumour volume was the most repeatable feature.

**Figure 4:**
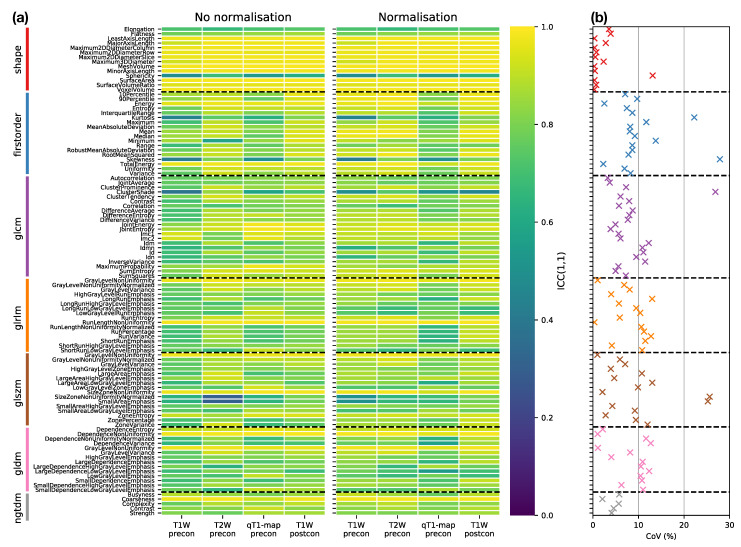
(**a**) ICC point estimates for all Box–Cox transformed features (rows), for each sequence (columns), from images without (left panel) and with (right panel) normalisation. Horizontal dashed black lines separate features in different classes. (**b**) Variability of ICCs across sequences and normalisations, plotted as the coefficient of variation (CoV) of ICCs over the eight datasets shown in the columns of (**a**). Features are colour-coded according to their class, and horizontal dashed black lines separate features in different classes.

Figure 4a provides an overview of the ICC results, plotting ICCs for all features, for each sequence, with and without normalisation, facilitating comparisons across all datasets. The dominance of yellow in the Shape class illustrates the tendency for higher ICCs for these features, though Sphericity has a notably lower ICC (∼0.58) across all sequences. Excluding Shape features, nine features have ICC >0.90 for all sequences, with and without normalisation): Energy, Total Energy, GLRLM Gray Level Non Uniformity, GLRLM Run Length Non Uniformity, GLSZM Gray Level Non Uniformity, GLSZM Size Zone Non Uniformity, GLDM Dependence Non Uniformity, GLDM Gray Level Non Uniformity, and NGTDM Coarseness. However, all these features are correlated with Mesh Volume (absolute Spearman’s ρ ranged from 0.62 to 0.97, across all sequences, with and without normalisation), suggesting these features offer limited independent information beyond tumour size. Figure 4b further summaries the impact of sequence choice and normalisation on repeatabilities, plotting the coefficient of variation (CoVs) in ICCs across all sequences, with and without normalisation. Shape features, with the exception of Sphericity, and NGTDM features tend to show the lowest variation, while features in other classes show greater variation in repeatability.

**Figure 5:**
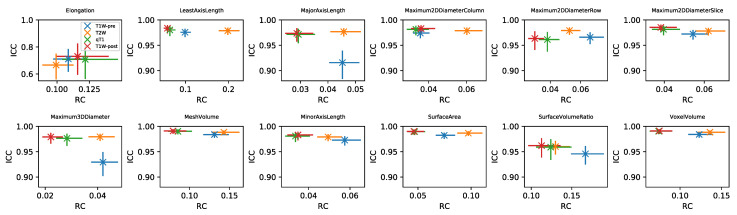
Plots of RC against ICC for Shape features, for four sequences (colours). In each panel, data points and error bars represent point estimates and 95% CIs.

RC values for all features and datasets are shown in Supplementary Information Figures S7–S14. Note that in general these values cannot be directly compared for different features and sequences, due to their differing units. For the subset of Shape features where RC values can be compared, Figure 5 plots RC against ICC for the four sequences. RC tends to show more variability between sequences than ICC, with qT${}_{1}$ and T${}_{1}$W-post tending to have lower RCs. While ICCs tend to be uniformly high across sequences, indicating good repeatability regardless of sequence, the lower RCs for qT${}_{1}$ map and T${}_{1}$W-post suggest these would be preferred for detecting longitudinal changes in Shape features. ICCs and RCs can therefore be seen to offer different perspectives on repeatability, with the choice of metric informed by the context of a particular study. Supplementary Information Figures S15–S20 plot RC against ICC for the other feature classes. For some features, ICCs and RCs are inversely correlated, suggesting they provide comparable information about repeatability (with higher ICCs and lower RCs indicating better repeatability); for others, however, correlations are not observed, showing that for certain features, a low RC does not necessarily imply a high ICC. For example, Energy and Total Energy from qT${}_{1}$ maps have relatively low RCs, but have the lowest ICCs of the four sequences. Conversely, for most other First Order features, qT${}_{1}$ maps exhibit the highest RC and lowest ICC (Figure S15). Again, it should be noted that the direct comparison of RCs for Box–Cox transformed features is confounded by having different optimal λ values for the different sequences.

As ICCs are more readily comparable across features and datasets, the following sections analyse ICCs in more detail, comparing non-normalised data across the different MR sequences, and investigating the effect of normalisation.

### 3.3. Comparing MR Sequences

**Figure 6:**
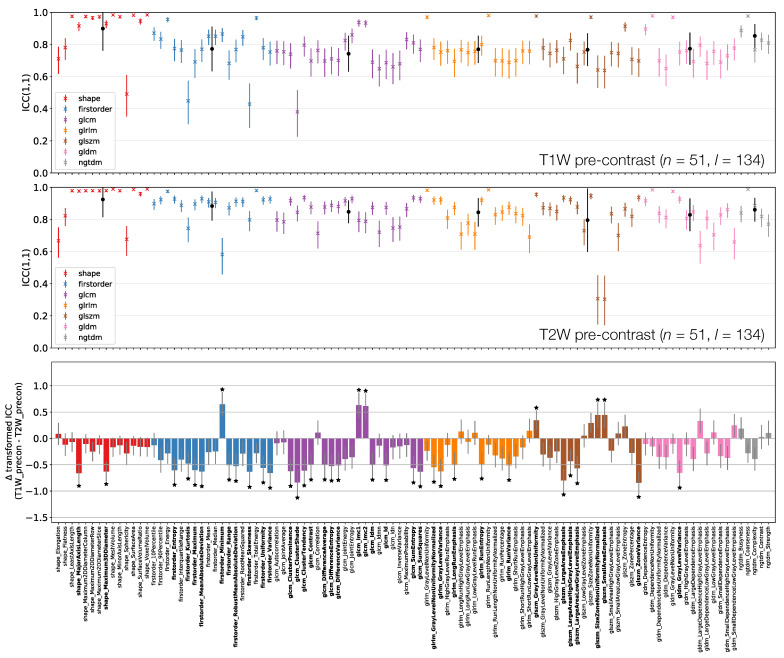
ICCs for Box–Cox transformed features from T${}_{1}$W (top) and T${}_{2}$W (middle) pre-contrast images. Data points and error bars represent ICC point estimates and 95% CIs. Features are colour coded according to their class and black points correspond to mean ± standard deviation ICCs over features within each class. The bottom panel represents the difference in ICCs (after applying Fisher’s *Z*-transformation) for T${}_{1}$W and T${}_{2}$W pre-contrast images. Black stars and bold fonts indicate features where ICCs from the two sequences are significantly different.

**Figure 7:**
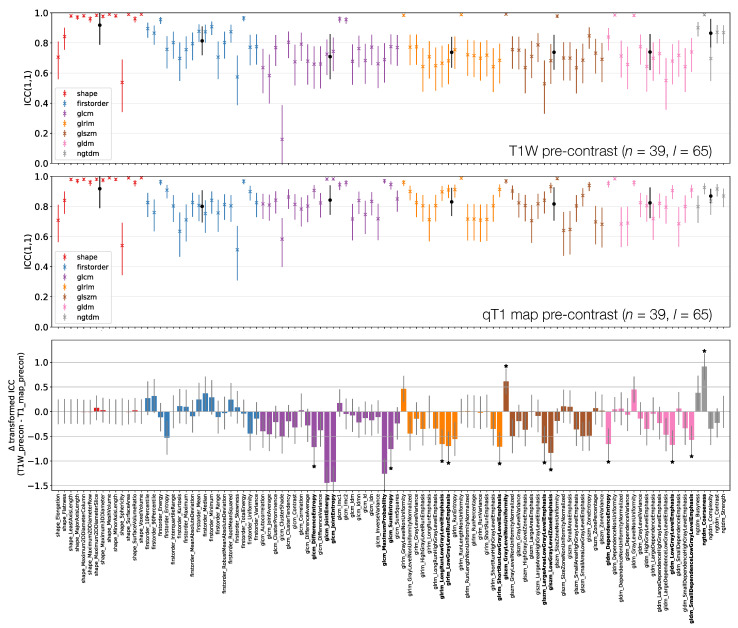
ICCs for Box–Cox transformed features from T${}_{1}$W (top) and qT${}_{1}$ map (middle) pre-contrast images. Data points and error bars represent ICC point estimates and 95% CIs. Features are colour coded according to their class and black points correspond to mean ± standard deviation ICCs over features within each class. The bottom panel represents the difference in ICCs (after applying Fisher’s *Z*-transformation) for T${}_{1}$W and qT${}_{1}$ map pre-contrast images. Black stars and bold fonts indicate features where ICCs from the two sequences are significantly different.

**Figure 8:**
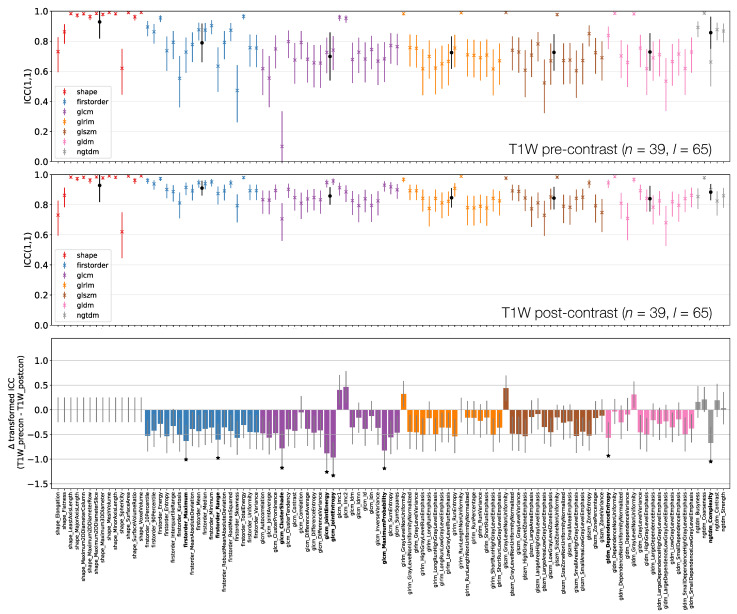
ICCs for Box–Cox transformed features from T${}_{1}$W pre-contrast (top) and T${}_{1}$W post-contrast (middle) images. Data points and error bars represent ICC point estimates and 95% CIs. Features are colour coded according to their class and black points correspond to mean ± ICCs over features within each class. The bottom panel represents the difference in ICCs (after applying Fisher’s *Z*-transformation) for T${}_{1}$W pre-contrast and T${}_{1}$W post-contrast. Black stars and bold fonts indicate features where ICCs from the two sequences are significantly different.

Figure 6, Figure 7 and Figure 8 plot ICCs for non-normalised images from the 4 MR sequences. Note that Figure 6 includes data from 134 lesions, while in Figure 7 and Figure 8 the presented data come from the subset of 65 T${}_{1}$W pre-contrast lesions that match those used in qT${}_{1}$ map and T${}_{1}$W post-contrast analyses. The bottom panel in Figure 6, Figure 7 and Figure 8 plot the differences in ICCs between two sequences, allowing comparison of pre-contrast anatomical images (T${}_{1}$W and T${}_{2}$W), comparison of pre-contrast anatomical and quantitative images (T${}_{1}$W and qT${}_{1}$ map), and comparison of pre- and post-contrast images (T${}_{1}$W pre- and post-contrast). These specific comparisons will be described below. As in Figure 3 above, note that these plots show the difference in ICCs after applying Fisher’s *Z*-transformation, and the error bars show the 95% CI on this difference.

#### 3.3.1. T${}_{1}$W Pre-Contrast and T${}_{1}$W Pre-Contrast

Figure 6 shows that shape feature ICCs tend to be very similar between T${}_{1}$W and T${}_{2}$W pre-contrast images. Point estimates for Sphericity are most dissimilar, although the relatively wide confidence intervals means this difference is not significant; Major Axis Length and Maximum 3D Diameter ICCs are significantly higher on T${}_{2}$W, though point estimates >0.91 on both images. Of the 38/105 features whose ICCs are significantly different, 10 are from the First Order class, with all but one of these (Minimum) exhibiting a higher ICC on T${}_{2}$W. Of the remaining 26 significantly different features in the texture classes, 21 exhibit a higher ICC on T${}_{2}$W.

#### 3.3.2. T${}_{1}$W Pre-Contrast and qT${}_{1}$ map Pre-Contrast

Figure 7 shows that First Order features have comparable ICCs from T${}_{1}$W pre-contrast images and qT${}_{1}$ maps. Shape features, and therefore their associated ICCs, are essentially identical here as the same mask was used for both sets of images, with minor differences due to the different resolutions (see Section 2.1); note that qT${}_{1}$ maps had a lower resolution than all other images. In all, 15/105 features have significantly different ICCs. Thirteen of these 15 show higher ICCs from qT${}_{1}$ maps, and while most features are not significantly different, the tendency is for ICCs to be higher for qT${}_{1}$ maps. Note that CIs tend to be wider on the T${}_{1}$W pre-contrast plot here compared with the top row of Figure 6, as fewer lesions were included in the analysis for this comparison.

#### 3.3.3. T${}_{1}$W Pre- and Post-Contrast

Figure 8 shows that all First Order features from T${}_{1}$W post-contrast images have higher ICCs than those from T${}_{1}$W pre-contrast images, though only two are statistically significant. This tends to be true for all other classes, with the vast majority of features exhibiting higher ICCs on T${}_{1}$W post-contrast images. Of the 8/105 features whose ICCs do differ significantly, all have ICCs which are higher on T${}_{1}$W post-contrast images. Note that Shape features here are identical for pre- and post-contrast images, as the same masks were used for both.

### 3.4. Effect of Normalisation

**Figure 9:**
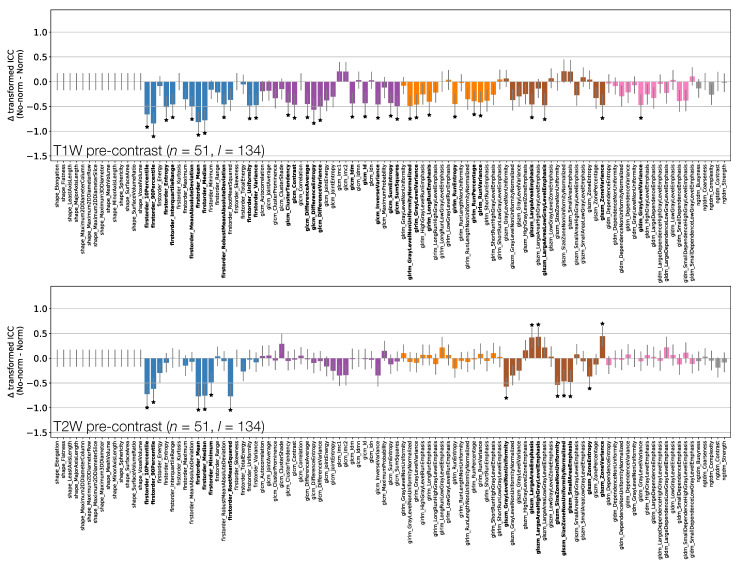
Effect of image normalisation on T${}_{1}$W and T${}_{2}$W pre-contrast images. Comparison of ICCs for Box–Cox transformed features from non-normalised and normalised images, for T${}_{1}$W (top) and T${}_{2}$W (bottom) pre-contrast images. Bars represent the difference in ICC point estimates (after applying Fisher’s *Z*-transformation), and error bars represent 95% CIs. Features are colour coded according to their class. Black stars and bold fonts indicate features where ICCs from non-normalised and normalised images are significantly different.

**Figure 10:**
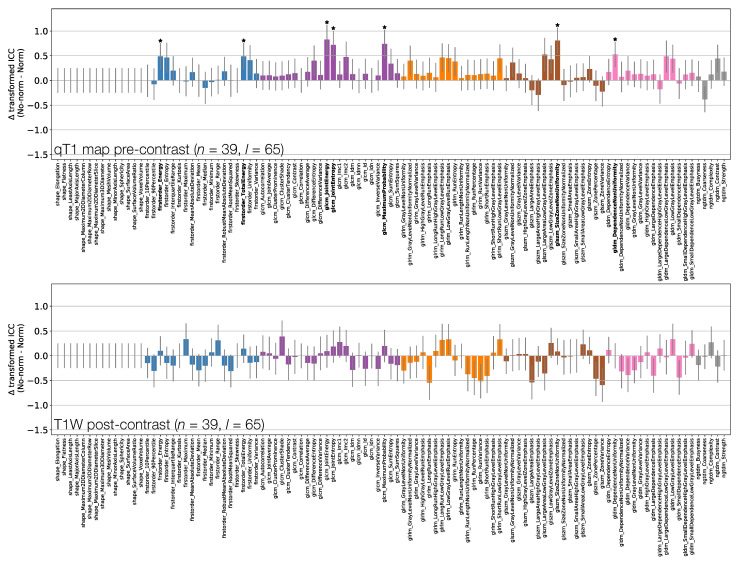
Effect of image normalisation on qT${}_{1}$ maps and T${}_{1}$W post-contrast images. Comparison of ICCs for Box–Cox transformed features from non-normalised and normalised images, for qT${}_{1}$ map pre-contrast (top) and T${}_{1}$W post-contrast (bottom). Bars represent the difference in ICC point estimates (after applying Fisher’s *Z*-transformation), and error bars represent 95% CIs. Features are colour coded according to their class. Black stars and bold fonts indicate features where ICCs from non-normalised and normalised images are significantly different.

Figure 9 and Figure 10 show how ICCs are affected by applying image normalisation prior to feature extraction, comparing ICCs between images with and without normalisation, for each sequence. Normalisation tends to affect T${}_{1}$W and T${}_{2}$W pre-contrast images more than qT${}_{1}$ maps or T${}_{1}$W post-contrast images, with 30 and 14 ICCs significantly changed by applying normalisation to T${}_{1}$W and T${}_{2}$W pre-contrast images, respectively, with only seven ICCs significantly affected on qT${}_{1}$ maps, and no ICCs significantly affected for T${}_{1}$W post-contrast. For T${}_{1}$W pre-contrast images, all ICCs which are significantly affected are higher when normalisation is applied; for T${}_{2}$W, 11 of the 14 ICCs significantly affected improve with normalisation. Conversely, for qT${}_{1}$ maps, although most ICCs are unaffected by normalisation, for the seven features which are significantly different, all have lower ICCs when normalisation is applied. Note that Shape features only depend on the masks, and so are unaffected by normalisation; their ICCs are therefore identical with and without normalisation.

### 3.5. Summary of Results

Taken together, the results from the present analysis highlight several aspects of radiomic feature repeatability which may be important to consider in future studies. Firstly, while most features had non-Gaussian distributions, the use of Box–Cox transformations enabled ICCs and RCs to be calculated appropriately for an average of 97% of features across sequences. Secondly, features exhibited a wide range of ICCs, with Shape features tending to have the highest ICCs. Thirdly, 19% of features from non-normalised images exhibited significantly different ICCs in pair-wise comparisons between different MR acquisitions. Fourthly, the use of image normalisation tended to increase ICCs for pre-contrast T${}_{1}$- and T${}_{2}$-weighted images, and decrease ICCs for qT${}_{1}$ maps. Finally, RCs and ICCs can provide different insights into feature repeatability.

## 4. Discussion

Evaluating repeatability is a key step in the technical validation of imaging biomarkers, which itself is essential for translating such biomarkers into clinical practice [4]. In the context of radiomics, repeatability is an important factor when determining which features should be included in a predictive model. For example, if a feature has poor single site repeatability it is unlikely that it will have good multi-centre reproducibility, limiting its utility in a model. Conversely, good single site repeatability can be seen as a necessary, but not sufficient, condition for utility, as multi-centre reproducibility would still need to be demonstrated. As such, feature repeatability is a prerequisite for contributing to a robust predictive signature, or use as a biomarker of treatment response. Importantly, a feature’s repeatability according to a particular metric may suggest its suitability for a particular use case, with a high ICC implying good performance when used as a diagnostic and/or predictive biomarker, whereas a low RC would be required when using a feature as a biomarker of treatment response. Note that what is considered ‘low’ in this context is dependent on the magnitude of the expected biomarker change, which needs to be considered alongside RC when evaluating the relative utility of features as biomarkers of change. Appropriately evaluating feature repeatability has practical consequences, as sample sizes and statistical power can be influenced by the repeatability of the biomarker; for example, a study using as a biomarker a feature with low repeatability would require a larger sample size than if a feature with high repeatability was used [28]. By assessing MR radiomic feature repeatability using two different metrics in a relatively large clinical cohort, investigating the effects of MR sequence, image normalisation, and assumptions about feature distributions, this work contributes to the technical validation of radiomic features. By focussing on liver metastases, and using quantitative T${}_{1}$ maps and post-contrast T${}_{1}$W images, this work complements existing repeatability studies using other MR sequences in other tumour types [12,14,15,16,17,18,19,20].

Evaluating repeatability using the metrics described in this work requires features to follow a Gaussian distribution, though this assumption is often not confirmed. This work found most radiomic feature distributions to be non-Gaussian, questioning the appropriateness of directly applying ICC or RC calculations. While ICCs are relatively robust to this assumption being invalid, it can make a significant difference for some radiomic features. As such, we suggest that examining feature distributions should form part of radiomic analyses, with results here demonstrating that Box–Cox transformations can be an effective way of obtaining Gaussian feature distributions. It is important to note that performing the Box–Cox transformation will alter some feature repeatability metrics significantly.

Across all datasets, Voxel Volume and Mesh Volume were the most repeatable features. As a class, Shape features tended to have the highest ICCs, consistent with observations on T${}_{2}$W images of cervical tumours [15], quantitative diffusion kurtosis maps of prostate tumours [20], and quantitative apparent diffusion coefficient maps of liver metastases and ovarian tumours [19]. For T${}_{2}$W images in rectal cancer, Gourtsoyianni et al. note that Gray Level Size Zone Matrix and Neighbouring Gray Tone Difference Matrix features tended to have poor repeatability [12]. In the present work, the features with the lowest ICCs on T${}_{2}$W were from the Gray Level Size Zone Matrix, while Neighbouring Gray Tone Difference Matrix features performed reasonably well (ICCs >0.77). Gourtsoyianni et al. did specifically note that Coarseness was an exception in terms of Neighbouring Gray Tone Difference Matrix features, which is consistent with the present work where it had ICC >0.93 for all sequences. Of the nine non-shape features with ICC >0.90 across all datasets in the present work, four (Energy, Total Energy, Run Length Non Uniformity, and Coarseness) were also found to have excellent repeatability and reproducibility in a phantom study using T${}_{2}$W images [36], providing further evidence of their robustness; note that in the phantom study these features tended to come from images filtered prior to feature extraction, while filtering was not investigated in the present work. Along with differences in filtering, the variation between studies in terms of MR sequence, image normalisation, and tumour type, make it challenging to directly compare repeatabilities across studies. Even comparing across the same type of MR sequence can be confounded by different studies using different parameters, with radiomic features showing sensitivity to echo time and repetition time in T${}_{2}$W acquisitions [36]. In the present work, the use of zero-filling during image reconstruction should also be noted, as this will tend to reduce intra-lesion heterogeneity in signal intensities, and hence impact many radiomic features. As the use of zero-filling differed between acquisitions, this will contribute to feature differences across sequences, in addition to the inherent MR weighting. Although not considered here, the reconstructed images could be resampled to achieve isotropic voxel sizes, which would be expected to impact texture features extracted in 3D. These points should also be noted in relation to multi-centre and multi-vendor reproducibility assessments, as precise acquisition and reconstruction details may vary between scanners, potentially impacting radiomic features. Further work is needed to understand the benefits of harmonising MR acquisitions and reconstructions for improving radiomic feature reproducibility, relative to post-acquisition harmonisation approaches [37]. Such approaches are especially relevant for retrospective studies where prospective acquisition harmonisation is not possible, and include the use of neural networks for pre-processing acquired images prior to feature extraction [37], and methods such as ComBat for mitigating the effects of feature variability related to specific centres or scanners [38].

Also note that in the present study, the nine non-shape features with ICC > 0.90 all showed strong correlations with Mesh Volume, which may contribute to their repeatability. Correlations between radiomic features and tumour volume have been reported previously [39]; indeed, it has been noted that using repeatability to guide feature selection may result in radiomic signatures which essentially reflect tumour volume [39]. As such, care must be taken to evaluate repeatability along with feature correlations.

In addition to their generally high ICCs, Shape features also tended to show the lowest variability in ICCs across sequences; this is to be expected as Shape features are insensitive to normalisation, and the same masks were used for T${}_{1}$-weighted images and qT${}_{1}$ maps. For a more comprehensive evaluation of Shape features, inter-observer variability in contouring could also be evaluated, and compared with test-retest repeatability; this would provide analogous data to that presented perviously for T${}_{2}$W images of cervical tumours [15], where Shape features had high ICCs for both test-retest repeatability (all but one feature having ICC >0.9) and inter-observer reproducibility (all features having ICC >0.9). The low inter-sequence variability in Shape ICCs observed here may also imply that Shape feature repeatability does not strongly depend on MR image contrast; note however that RC values for Shape features tended to show more variation across sequences, implying that the contrast can impact repeatability. (This effect of contrast can only stem from the fact that tumours may be more readily distinguished from surrounding tissue on some sequences, facilitating more repeatable delineation; once contours are defined, Shape features are independent of signal intensities). In general, ICCs and RCs provide different information about feature repeatability, and the choice of metric needs to be considered in a study-specific context. Practically, this means that a given feature from a given sequence may have favourable repeatability characteristics for one application (for example, as a diagnostic or predictive biomarker), but less favourable characteristics for another (for example, as a biomarker of treatment response).

The ICCs of non-shape features tended to exhibit greater sensitivity to sequence and normalisation, particularly for Skewness, Cluster Shade, Small Area Emphasis and Size Zone Non-Uniformity Normalized. As such, it may be expected that the repeatability of such features may vary more across different studies, if different MR acquisitions and normalisation approaches are used, necessitating study-specific repeatability analyses. Furthermore, unlike for RC, a feature’s ICC depends on the inter-subject variability of that feature [28], meaning that repeatability as evaluated from ICCs may vary across different cohorts, even for the same sequence and normalisation, further motivating repeatability assessments on a per-study basis using the most appropriate metric for that study.

When comparing ICCs from non-normalised T${}_{1}$W and T${}_{2}$W pre-contrast images, First Order features tended to show better repeatability on T${}_{2}$W. This could reflect easier lesion definition on T${}_{2}$W images, leading to more repeatable signal intensity distributions, or could be due to T${}_{1}$W signals having sensitivity to genuine changes in the lesions between scans, resulting in apparently poorer repeatability. Normalisation tended to improve repeatability for both sequences, with ICCs increasing significantly in 29% and 13% of features for T${}_{1}$W and T${}_{2}$W, respectively. This improvement with normalisation would be expected if repeated scans differed by a uniform scaling factor. However, it should not be assumed that this is the only difference, as non-uniformities may be present that could vary between visits, for example through $B_{0}$ or $B_{1}$ inhomogeneities [40]. Although the differences are not dramatic, these ICC results suggest a slight preference of T${}_{2}$W over T${}_{1}$W images for obtaining repeatable radiomic features in this dataset.

As image signal intensities have no inherent units in MR, it cannot be assumed that they can be directly compared across different sequences, or even across repeated acquisitions of the same sequence. A key motivation in the use of quantitative MR techniques is their ability to yield biomarkers which are independent of absolute image signal intensities. However, comparing First Order features between non-normalised T${}_{1}$W and qT${}_{1}$ pre-contrast images showed no significant differences in repeatability, implying that signal intensities are as comparable as quantitative T${}_{1}$ values; note that this must be considered in the context of the present study, where a single scanner was used, with the same acquisitions performed for all repeat scans. While quantitative values may be expected to be more repeatable than signal intensities, quantitative maps may be more sensitive to motion, given that T${}_{1}$ is here quantified through modelling signals across three separate acquisitions. Radiomic features will in general be affected by motion artefacts, which are especially relevant for free-breathing liver acquisitions, and these effects may differ between weighted and quantitative images. As applying normalisation had relatively little effect on repeatabilities from qT${}_{1}$ maps, but tended to improve those from T${}_{1}$W images, the use of normalisation resulted in T${}_{1}$W images yielding higher ICCs than qT${}_{1}$ maps for all First Order features, with eight of these being significantly higher. This highlights the different effects of normalisation on weighted and quantitative images. It could be argued that normalisation is more appropriate for weighted images, as it aims to correct for potential scaling differences between signal intensities, while quantitative maps are insensitive to such scalings. Contrary to this, it has been reported that normalisation improved repeatability for quantitative maps (the apparent diffusion coefficient), but not for weighted images (T${}_{2}$W) [17]. Further work is therefore needed to fully understand the use of quantitative maps, as opposed to weighted images, as radiomics inputs. This may be especially important for multi-centre studies, where comparison of signal intensities across scanners is likely to be more problematic than for a single scanner, and quantitative maps may be expected to yield more reproducible features. Although only a simple linear normalisation has been used in the present work, it should be noted that there are many approaches that could be employed [41,42,43,44]. The issue of normalisation is also especially relevant when seeking to compare radiomic features over time, for example in assessing treatment response; here, the use of normalisation could confound genuine signal intensity changes. Given the conflicting results and importance of this step, further investigation of normalisation techniques, along with the use of quantitative maps, is warranted. The potential for using normalised voxel sizes could also be investigated, and may be especially relevant for comparisons between anatomical images and quantitative maps, given that the former would typically be acquired at a higher resolution.

Although most differences are not statistically significant, there is a tendency for higher ICCs on T${}_{1}$W post-contrast images than on T${}_{1}$W pre-contrast images. This cannot be explained by improved lesion conspicuity post-contrast, as the same masks were used for both sequences and were drawn on pre-contrast images. The trend for better repeatability could be related to a higher signal-to-noise ratio post-contrast, due to T${}_{1}$ shortening, or could reflect an increased inter-subject variability due to differences in contrast agent uptake. Of the four sequences assessed here, T${}_{1}$W post-contrast features were least affected by normalisation, with no features showing significant differences. Note that throughout this study, statistical significance was judged based on a Bonferroni-adjusted *p*-value which accounted for the dominant source of multiple comparisons, namely, the number of features extracted. Given the exploratory nature of the investigation, further adjustments for comparisons between different sequences were not included; these could be incorporated into future prospective comparison studies to lower the threshold for determining significance.

The main limitations of the study are that repeatability was assessed over a period of 2–7 days, and that motion correction techniques were not applied. By having up to a week between repeat scans for some patients, it is possible that tumours may have undergone genuine macroscopic or microscopic changes, which radiomic features may be sensitive to. This could lead to an underestimate of feature repeatability, and could be mitigated by performing test-retest studies with features evaluated from scans performed minutes apart [15,16,20]; note however that this would not be feasible for T${}_{1}$W post-contrast scans, as the contrast agent administration cannot be repeated in such a short time window. As liver motion could affect the comparison between different sequences, future work could investigate the impact of applying motion correction before extracting radiomic features from multiple sequences. Finally, in the context of biomarker validation, multi-centre and multi-vendor reproducibility is an essential next step. Comparison across scanners may be especially important for radiomic features which directly depend on signal intensities, as these are unlikely to be directly comparable across scanners. While the use of quantitative parametric maps removes the dependence on signal intensities, quantitative T${}_{1}$ values in the human brain have been reported to differ between vendors [45], indicating that there is also a need for inter-vendor comparisons of radiomic features from quantitative maps.

## 5. Conclusions

Radiomic features from colorectal cancer liver metastases exhibit a wide range of repeatabilities. Some, but not all, of these can be significantly affected by the choice of MR sequence, image quantitation, use of contrast agent, and image normalisation. The presence, magnitude and direction of significant changes due to these factors is not readily predictable without conducting such an analysis in a test cohort, as performed here for one study scenario. The choice of repeatability metric can influence conclusions regarding feature repeatability, and the study context should be used to determine the most appropriate metric. These general principles are likely to extend to other MR studies using different sequences, suggesting that feature-specific repeatability, from specific combinations of MR sequence and pre-processing steps, should be evaluated with the most appropriate metric in order to select robust radiomic features as biomarkers in specific studies.

## Figures and Tables

**Table 1:** Effectiveness of Box–Cox transformations, assessed by the number of features with distributions consistent with a Gaussian distribution in different scenarios, for four MR sequences (rows), without and with normalisation (left and right). The first, second, and third column on each side show the number of features whose original distribution was Gaussian (Pre Box–Cox), the number of features whose original non-Gaussian distribution was transformed to Gaussian (Post Box–Cox), and the number of features whose distribution was non-Gaussian before and after applying transformations (Never). In all cases the total is 105, reflecting the number of features extracted.

	No Normalisation			Normalisation		
	Pre Box–Cox	Post Box–Cox	Never	Pre Box–Cox	Post Box–Cox	Never
T${}_{1}$W pre-contrast	22	77	6	17	81	7
T${}_{2}$W pre-contrast	17	84	4	15	87	3
qT${}_{1}$ map pre-contrast	25	78	2	27	77	1
T${}_{1}$W post-contrast	26	77	2	34	70	1

## Data Availability

Extracted radiomic features can be found at https://gitlab.com/manchester_qbi/manchester_qbi_public/radiomics_repeatability.

## Supplementary Materials

The following are available online at https://www.mdpi.com/2072-6694/13/2/240/s1, Figure S1, example masks for T1W pre-contrast, qT1 map pre-contrast, and T1W pre-contrast subset; Figures S2–5, feature histograms, Box-Cox normality plots, transformed feature histograms, and QQ-plots for T1W pre-contrast, T2 pre-contrast, qT1 map pre-contrast, and T1W post-contrast; Figure S6, effect of applying Box-Cox transforms to feature distributions, for qT1 map pre-contrast and T1W post-contrast; Figures S7–14, Bland-Altman plots with RC values for all features and datasets; Figures S15–20, plots of RC against ICC for each feature class except Shape.
