# Supplementary material for: Image Contrast, Image Pre-Processing, and T1 Mapping Affect MRI Radiomic Feature Repeatability in Patients with Colorectal Cancer Liver Metastases
Source: Cancers (Basel). 2021 Jan 11;13(2):240. doi: 10.3390/cancers13020240 (PMC7826650; doi:10.3390/cancers13020240)
Supplement: Supplementary file 1 [file cancers-13-00240-s001.zip › MDPI_template_Cancers_radiomics_supporting_information_submission_071220/supporting_information.pdf]

Supplementary Information

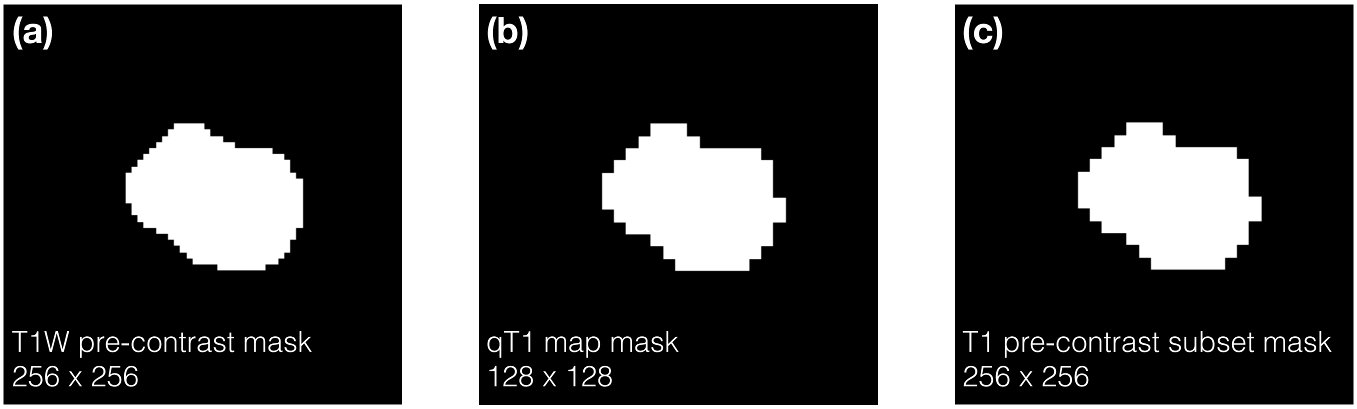

FIG. S1. Example masks for **(a)** T1W pre-contrast, **(b)** qT1 map, and **(c)** T1W pre-contrast subset. Note that **(a)** and **(b)** were generated based on the same ROI (defined on a T1W pre-contrast image), while **(c)** was generated by upsampling **(b)**, to allow for a direct comparison (in terms of masks applied) between qT1 maps and the T1W pre-contrast subset.

Choice of ICC

In order to assess whether there was a time effect or systematic difference between scans, ICC(1,1) and ICC(1,2) [1] were statistically compared (these are equivalent to ICC(1) and ICC(A,1) as defined in [2]). This was performed by fitting mixed effects models without and with a random effect for visit (where visits 1 and 2 refer to a subject's first and second scan), using the *lme4* package in R [3, 4], to Box-Cox transformed features. The simpler model without a random effect for visit was favoured in all cases when comparing model fits; this, along with the observation that ICCs from both models were very similar (maximum absolute difference of 0.06), lead to the use of ICC(1,1) in all cases for consistency. Point estimates and 95% confidence intervals (CIs) were calculated as described by McGraw and Wong [5]; these analytically calculated CIs were compared with those from 1000 bootstraps calculated using *rptR* [6], with very good agreement between the two (maximum absolute difference in lower and upper limits of 0.02 and 0.007, respectively). Mixed effects models in *rptR* were also used to investigate the effect of accounting for the fact that patients have different numbers of lesions, by specifying models where lesions were nested within patients. ICCs calculated from these nested models generally showed negligible difference (maximum absolute difference of 0.008) from those calculated without nesting, that is, when treating all lesions as independent; as such, all subsequent analyses used ICC(1,1), which assumes all lesions are independent.

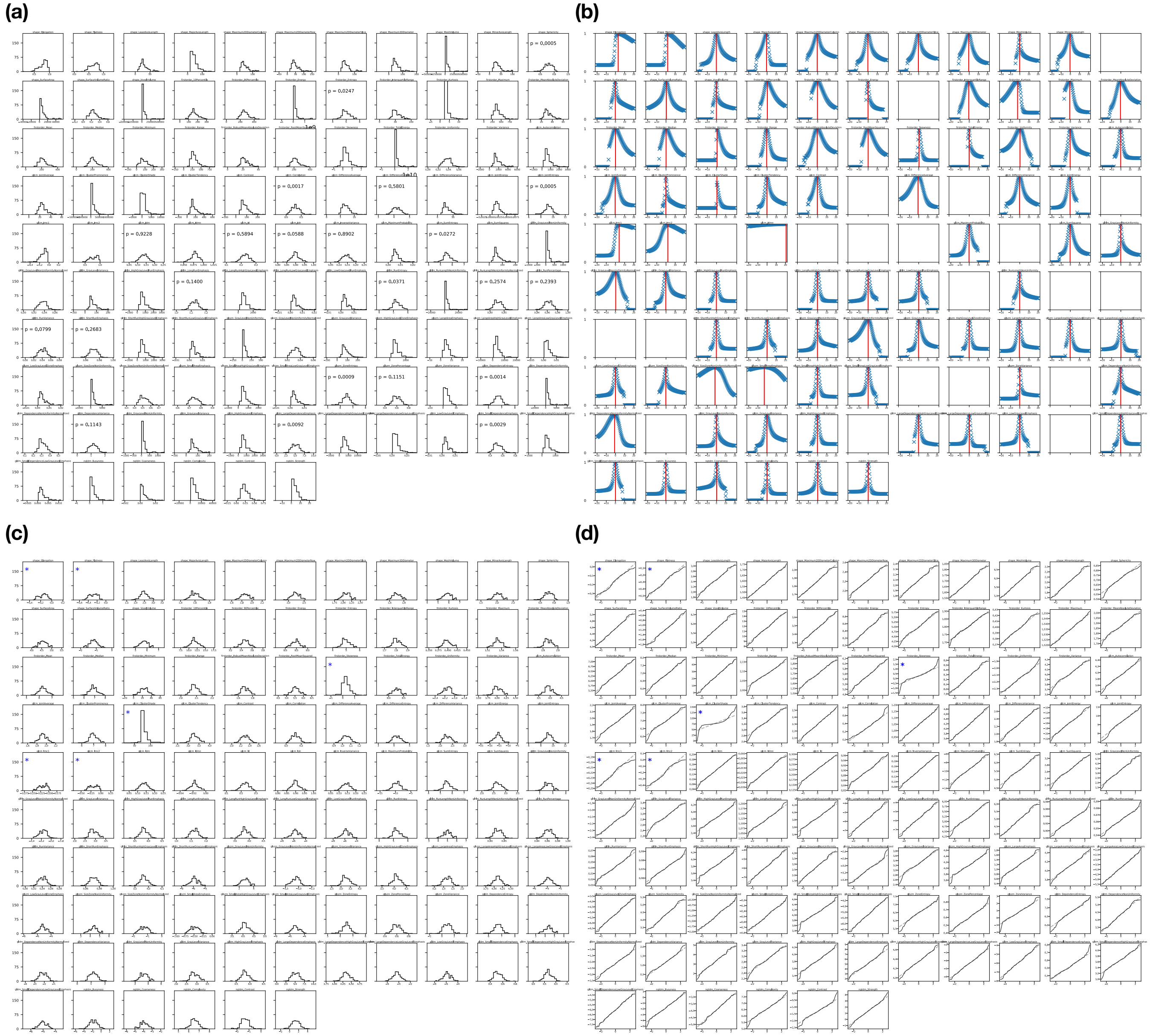

FIG. S2. **(a)** Histograms for each feature from T1W pre-contrast images, pooling feature values from both visits for all subjects. The majority of features have non-normal distributions, based on the Shapiro-Wilk test. Features which have distributions consistent with a normal distribution are indicated with the p-value for the test, which is greater than the Bonferroni-corrected threshold ($p > 0.05/105$). **(b)** Box-Cox normality plots for each feature distribution in **(a)**. Red vertical lines indicate the optimal λ to use to transform the distribution; empty panels correspond to the features whose original distributions were consistent with a normal distribution and therefore do not require a transform. **(c)** Histograms for each transformed feature from T1W pre-contrast images, pooling feature values from both visits for all subjects. Distributions come from applying optimal λ parameters shown in **(b)** to the original feature distributions shown in **(a)**, for features whose original distribution were non-normal. Features whose original distributions were consistent with a normal distribution did not undergo transformation. Features whose transformed distributions are not consistent with a normal distribution are indicated with a blue asterisk. **(d)** Q-Q plots for each feature distribution in **(c)**).

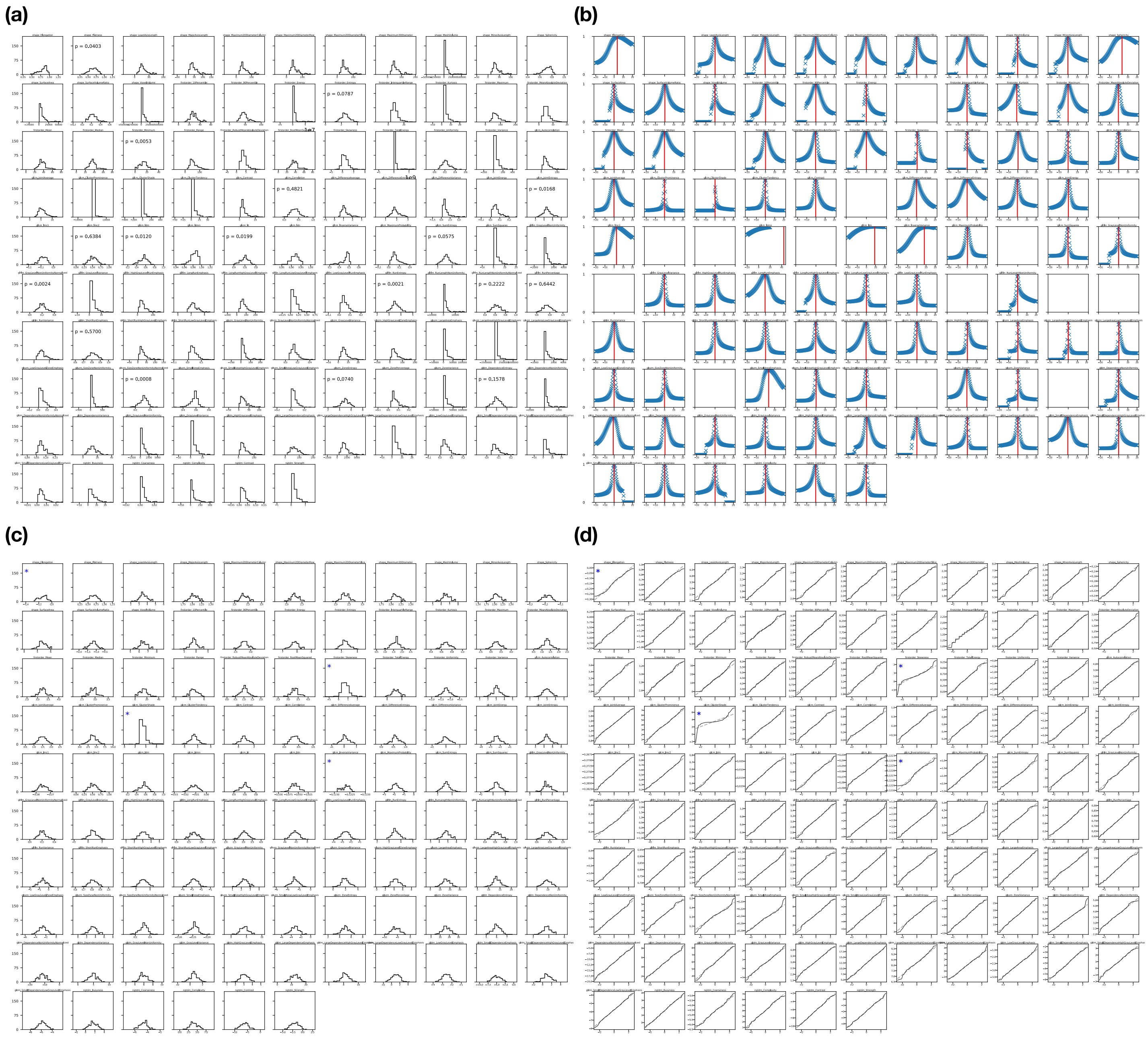

FIG. S3. As Figure S2 but for T2W pre-contrast images.

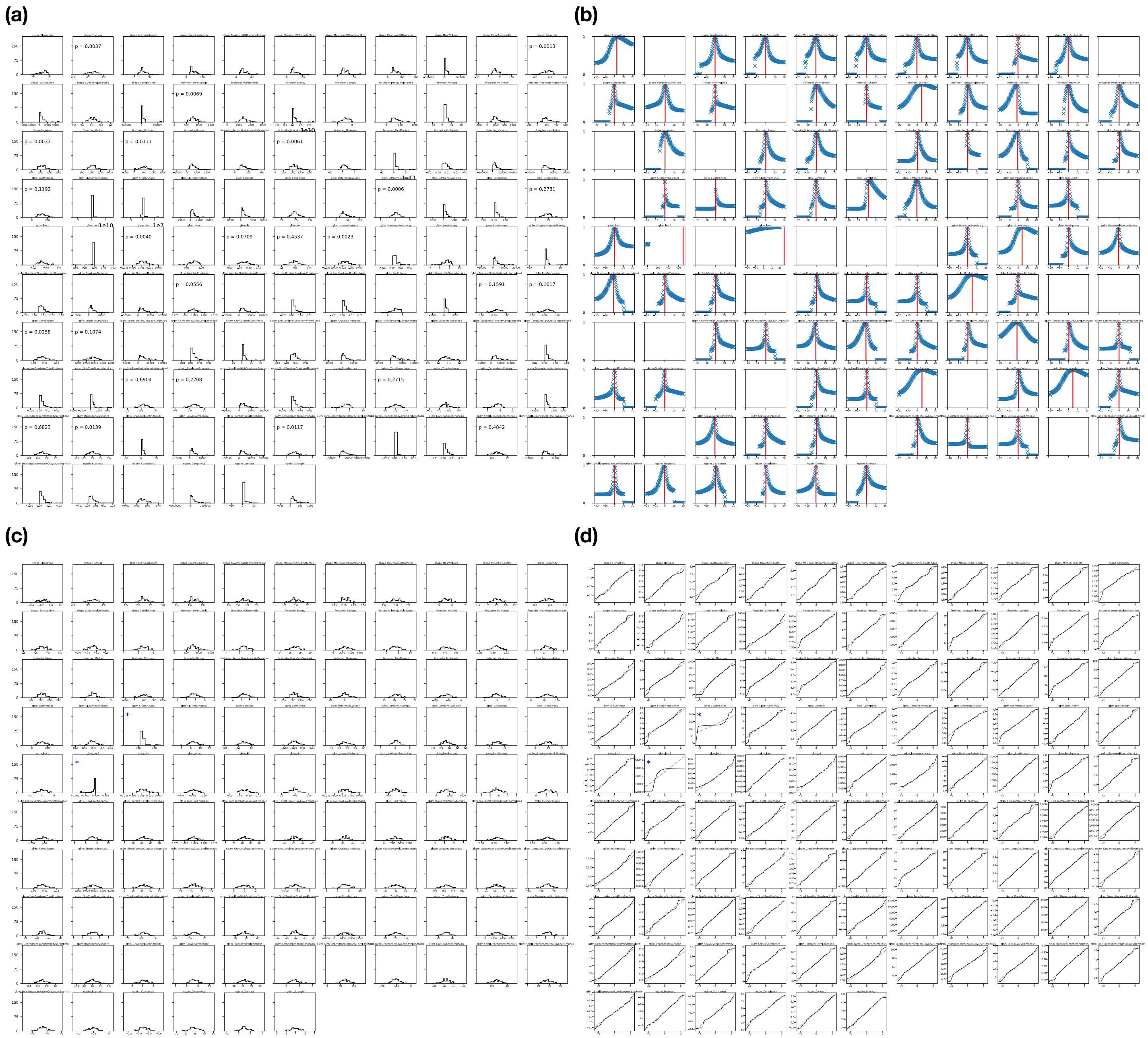

FIG. S4. As Figure S2 but for pre-contrast qT1 maps.

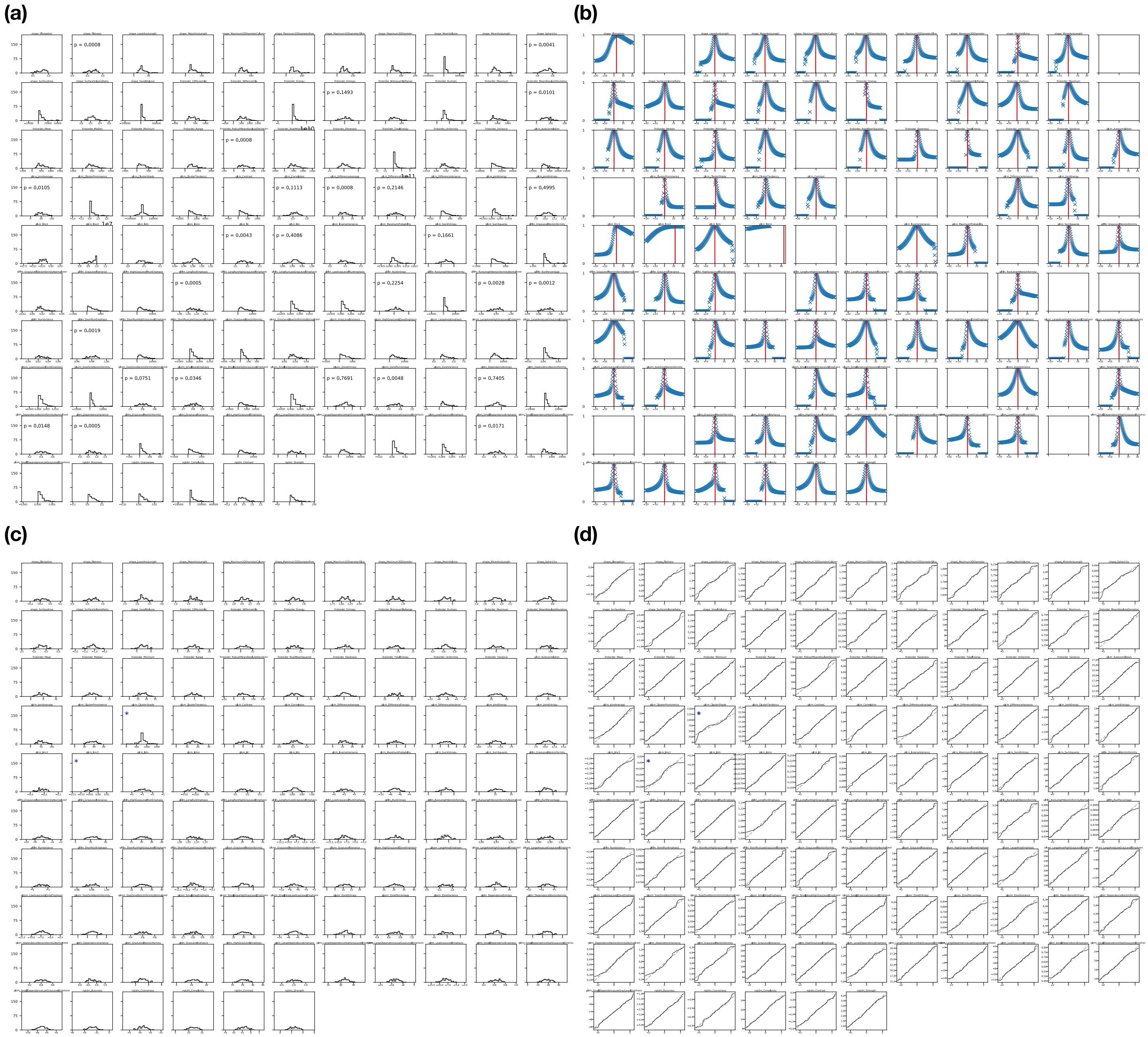

FIG. S5. As Figure S2 but for T1W post-contrast images.

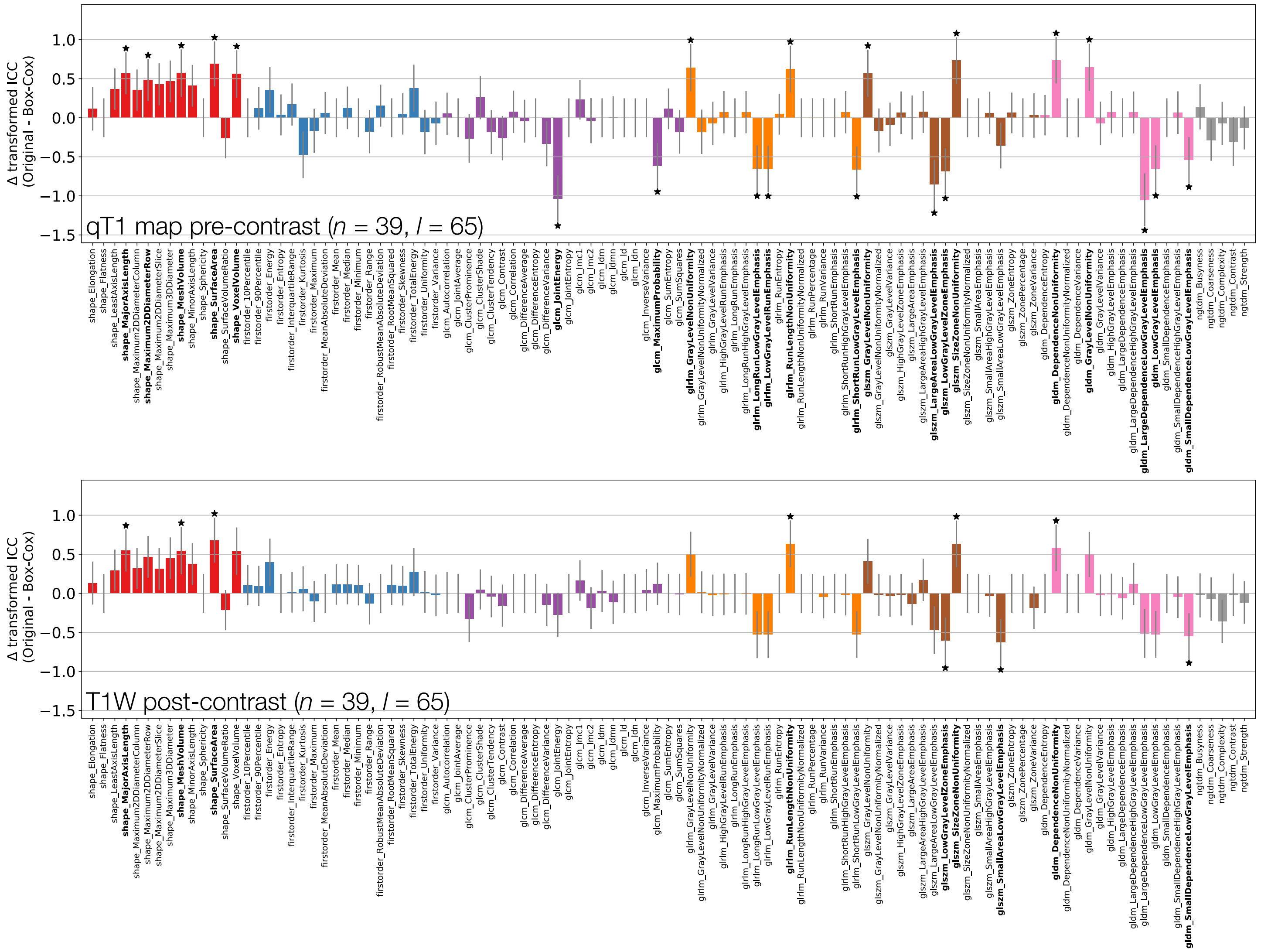

FIG. S6. Effect of applying Box-Cox transforms to feature distributions, for qT1 map pre-contrast (top) and T1W post-contrast (bottom) images. Bars represent the difference in ICC point estimates (after applying Fisher's Z-transform), and error bars represent 95% CIs. Features are colour coded according to their class. Black stars and bold fonts indicate features where ICCs from original and Box-Cox transformed data are significantly different.

Repeatability coefficients

Figures S7-S14 below show Bland-Altman plots for all features and all datasets, with RC values and CIs indicated in each panel. Figures S15-S20 show RC values plotted against ICC values for each non-Shape feature class, for normalised datasets. The subset of features in each class correspond to those for which Box-Cox transformations were consistently applied across the four sequences (see *Repeatability analysis and statistical comparison* in the main text).

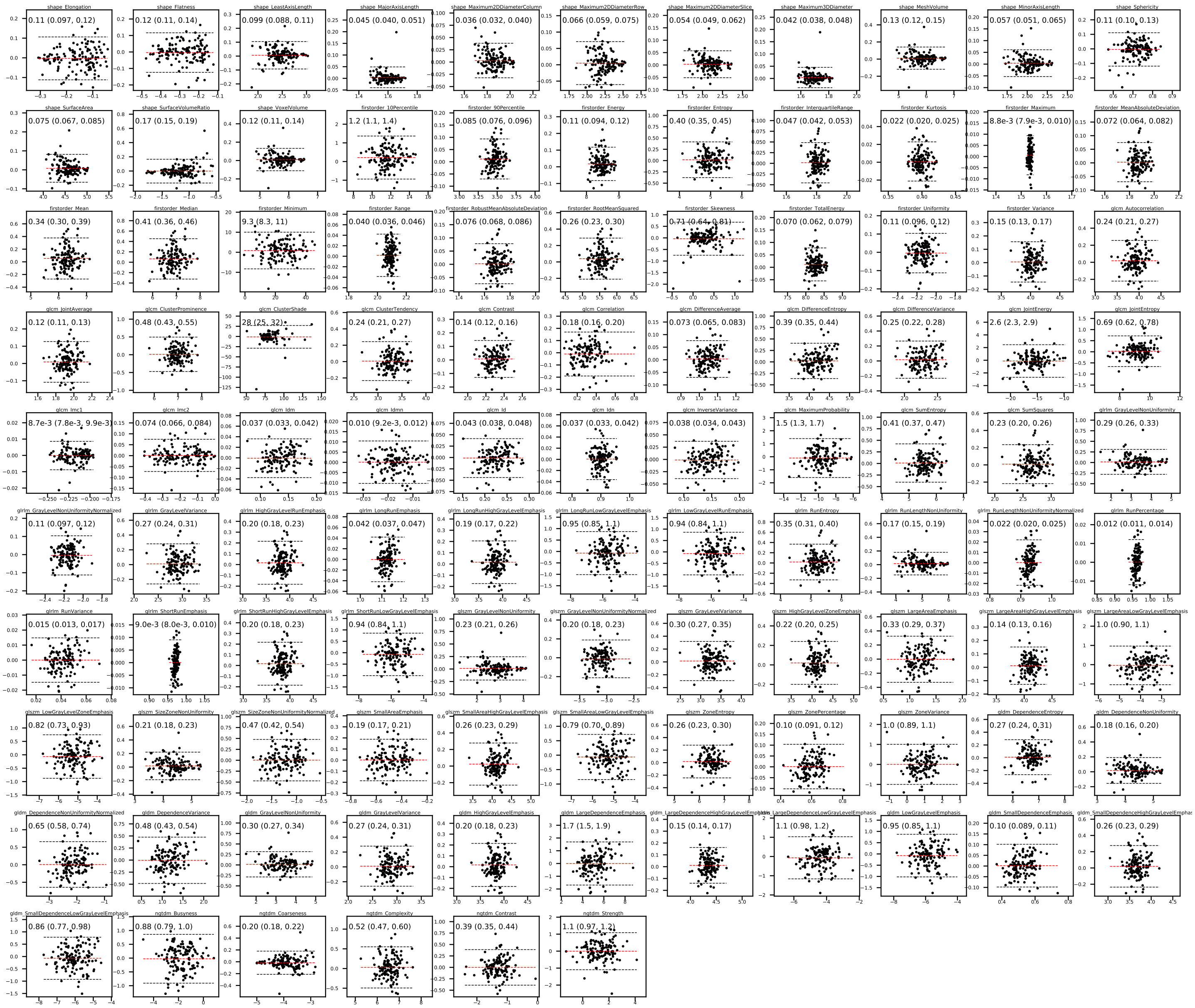

FIG. S7. Bland-Altman plots for T1W pre-contrast images. For each feature, the difference between visits (visit 2 − visit 1) is plotted against the mean of the two visits. Red and black dashed lines indicate the mean difference and 95% limits of agreement, respectively. In each panel, the feature's RC and CIs are quoted.

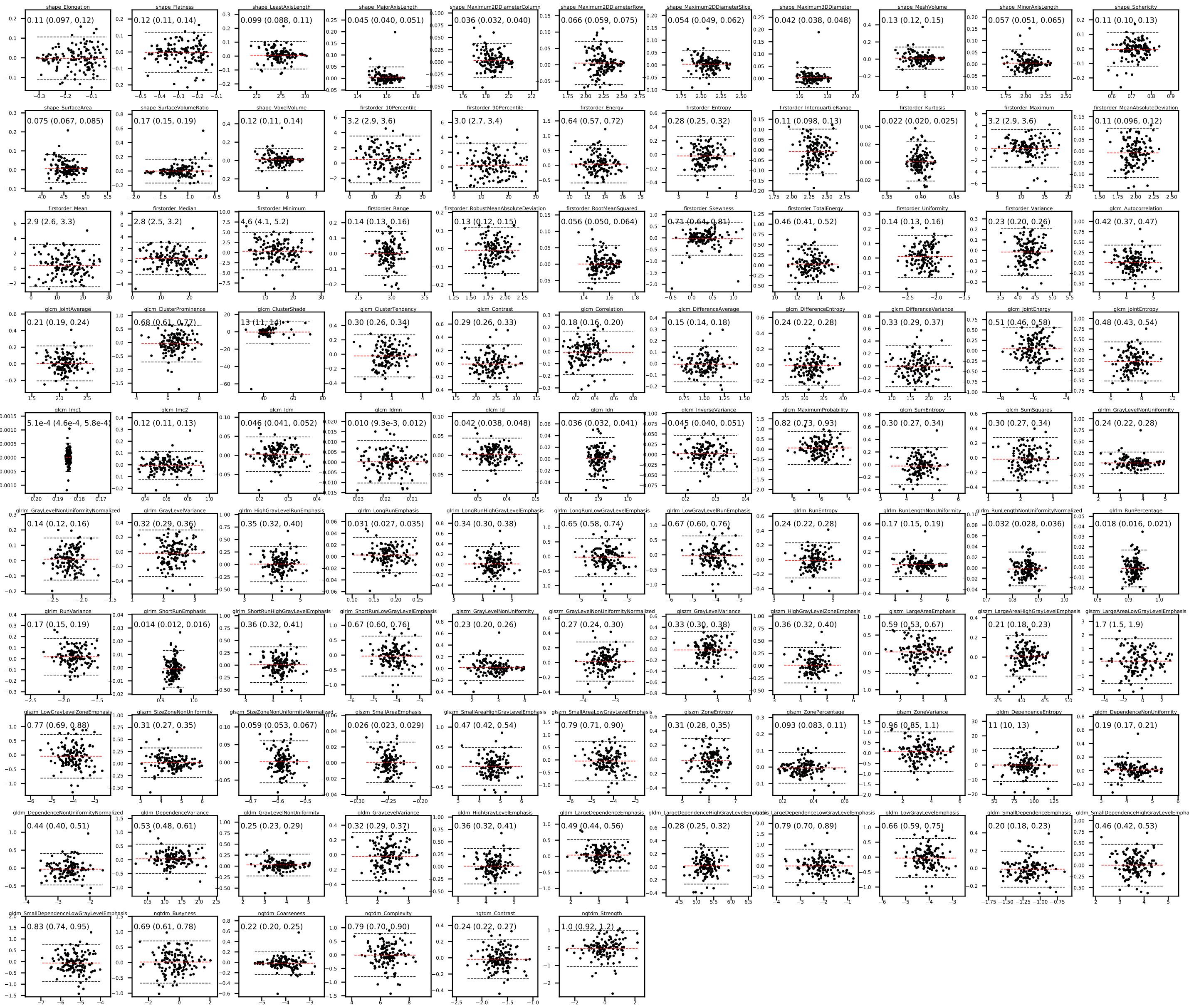

FIG. S8. Bland-Altman plots for normalised T1W pre-contrast images. For each feature, the difference between visits (visit 2 − visit 1) is plotted against the mean of the two visits. Red and black dashed lines indicate the mean difference and 95% limits of agreement, respectively. In each panel, the feature's RC and CIs are quoted.

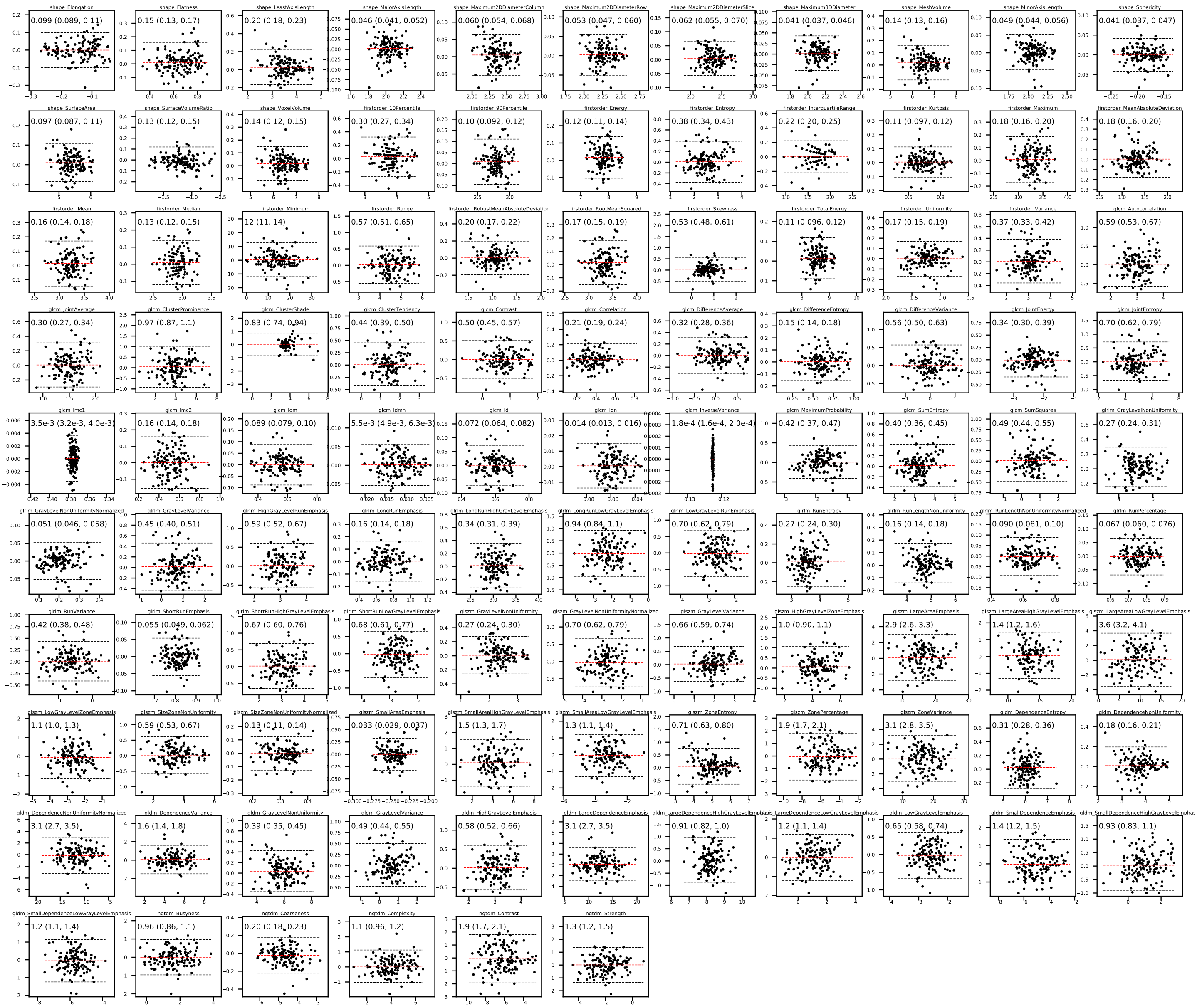

FIG. S9. Bland-Altman plots for T2W pre-contrast images. For each feature, the difference between visits (visit 2 − visit 1) is plotted against the mean of the two visits. Red and black dashed lines indicate the mean difference and 95% limits of agreement, respectively. In each panel, the feature's RC and CIs are quoted.

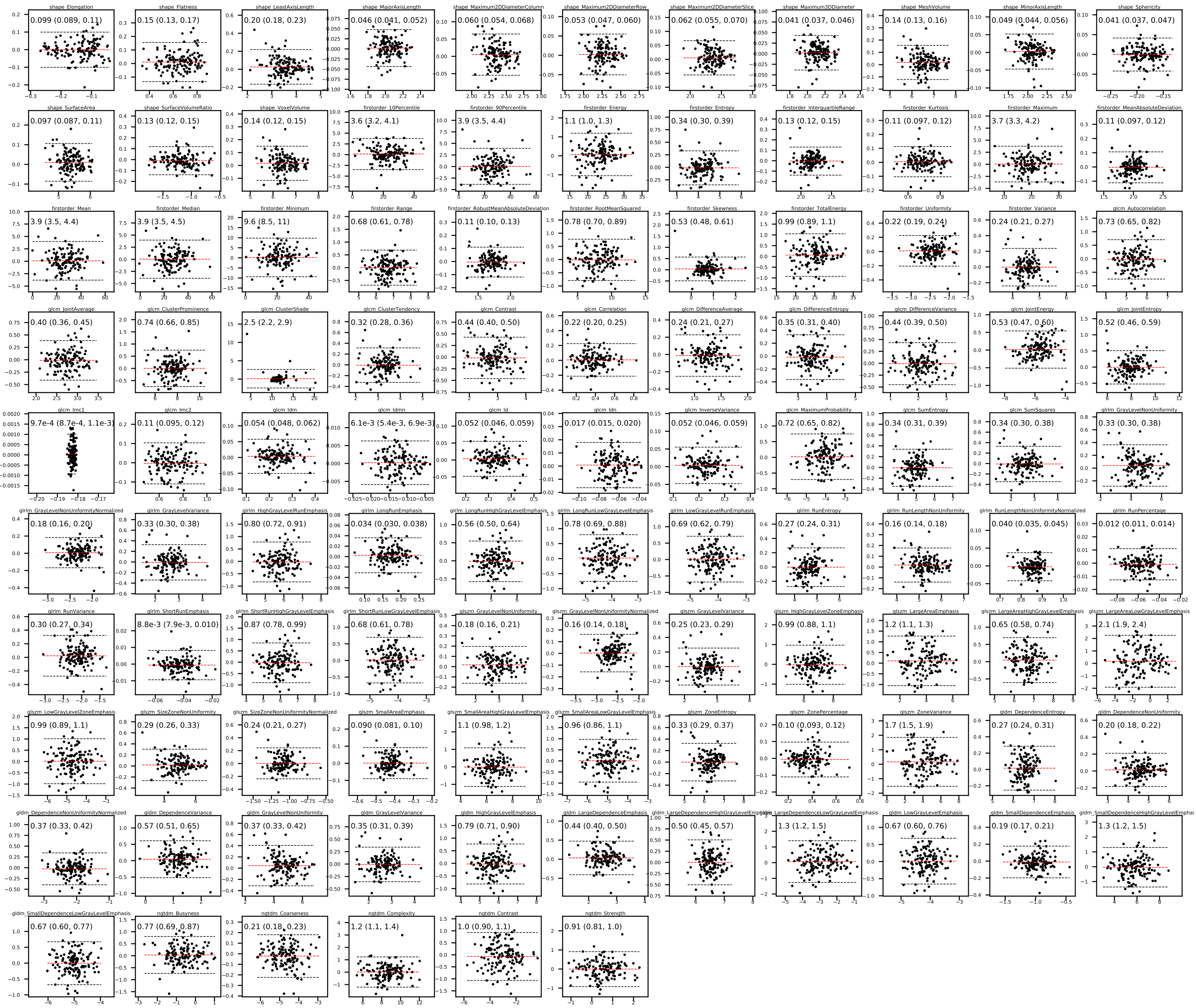

FIG. S10. Bland-Altman plots for normalised T2W pre-contrast images. For each feature, the difference between visits (visit 2 − visit 1) is plotted against the mean of the two visits. Red and black dashed lines indicate the mean difference and 95% limits of agreement, respectively. In each panel, the feature's RC and CIs are quoted.

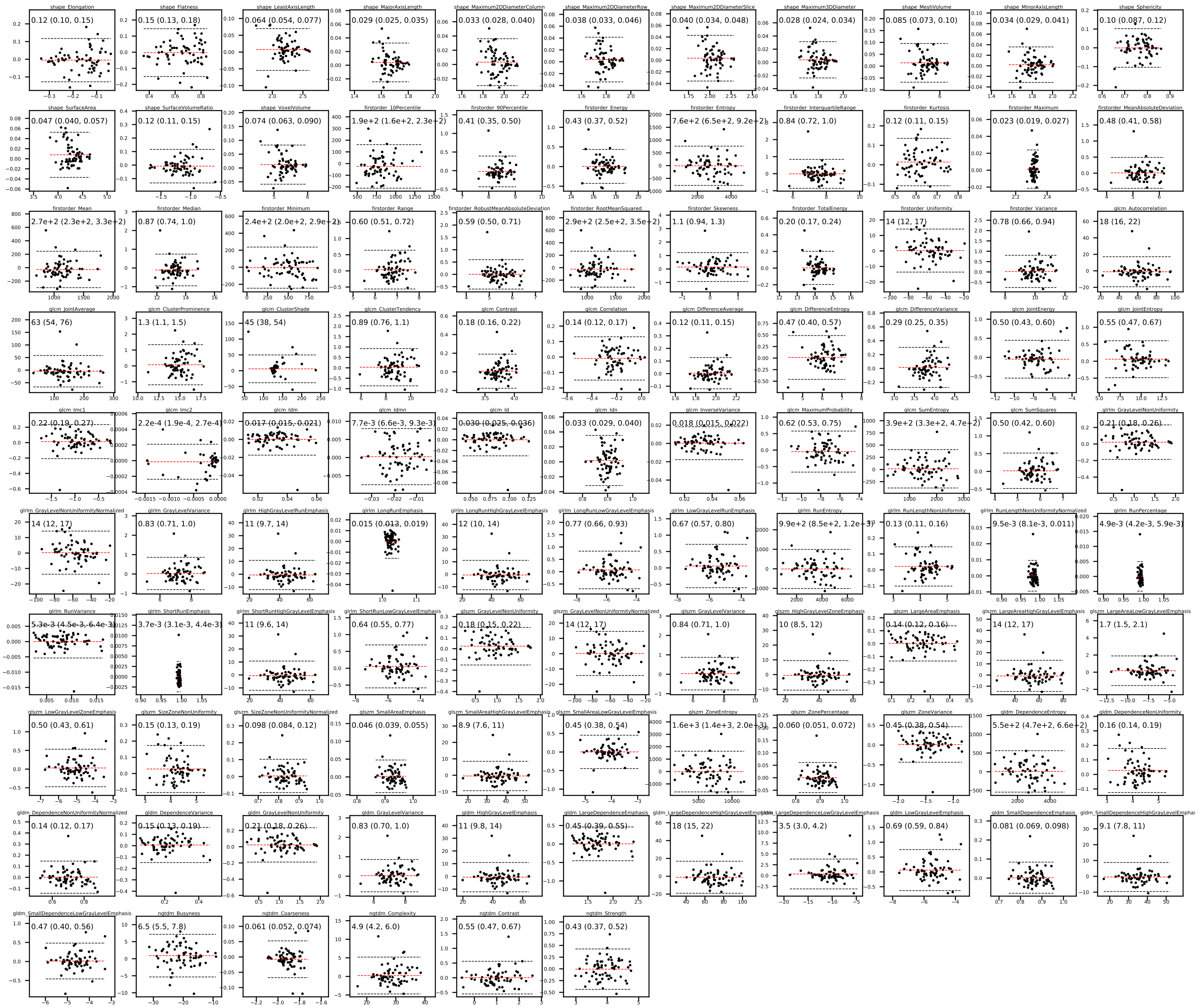

FIG. S11. Bland-Altman plots for qT1 pre-contrast images. For each feature, the difference between visits (visit 2 − visit 1) is plotted against the mean of the two visits. Red and black dashed lines indicate the mean difference and 95% limits of agreement, respectively. In each panel, the feature's RC and CIs are quoted.

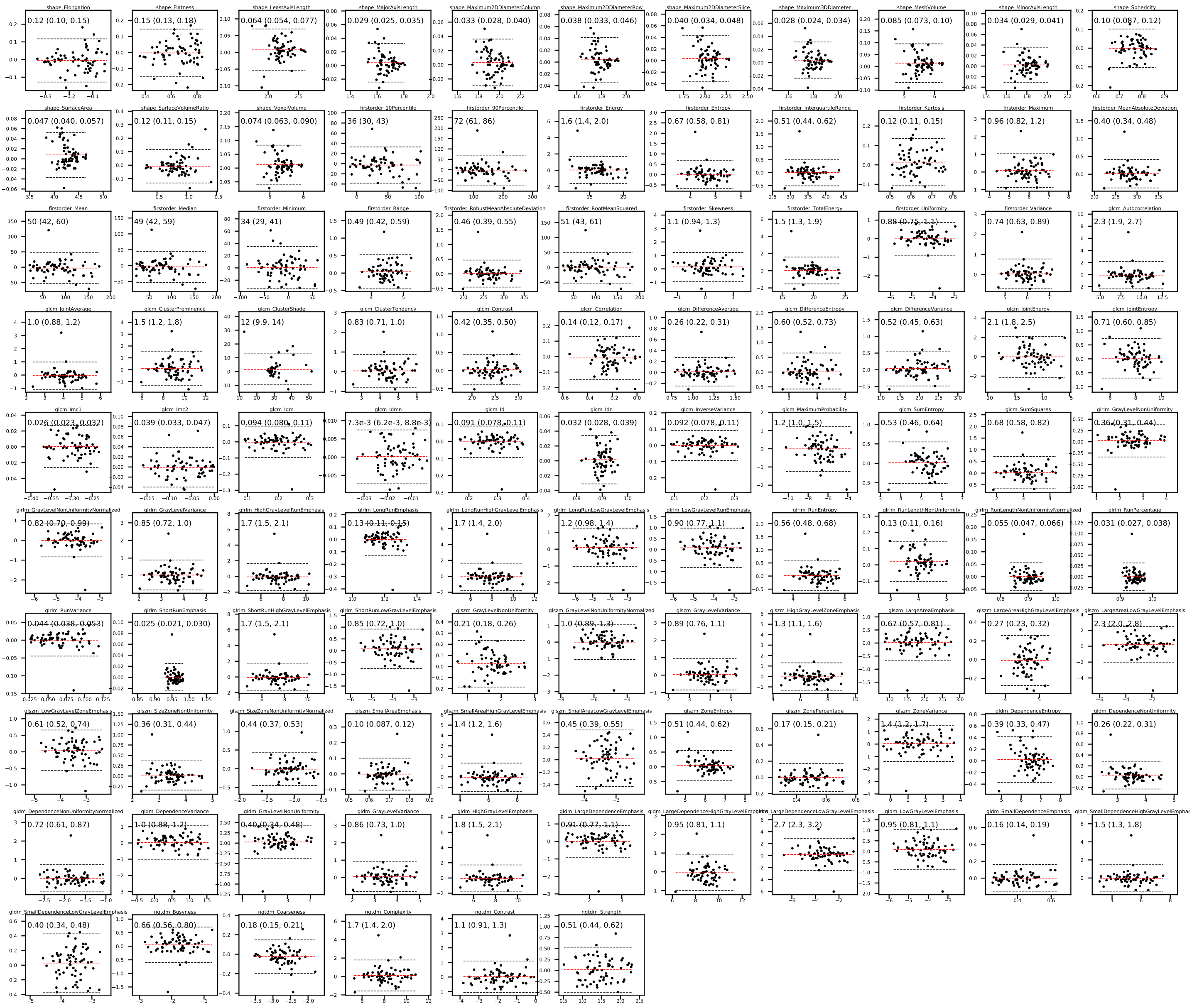

FIG. S12. Bland-Altman plots for normalised qT1 pre-contrast images. For each feature, the difference between visits (visit 2 − visit 1) is plotted against the mean of the two visits. Red and black dashed lines indicate the mean difference and 95% limits of agreement, respectively. In each panel, the feature's RC and CIs are quoted.

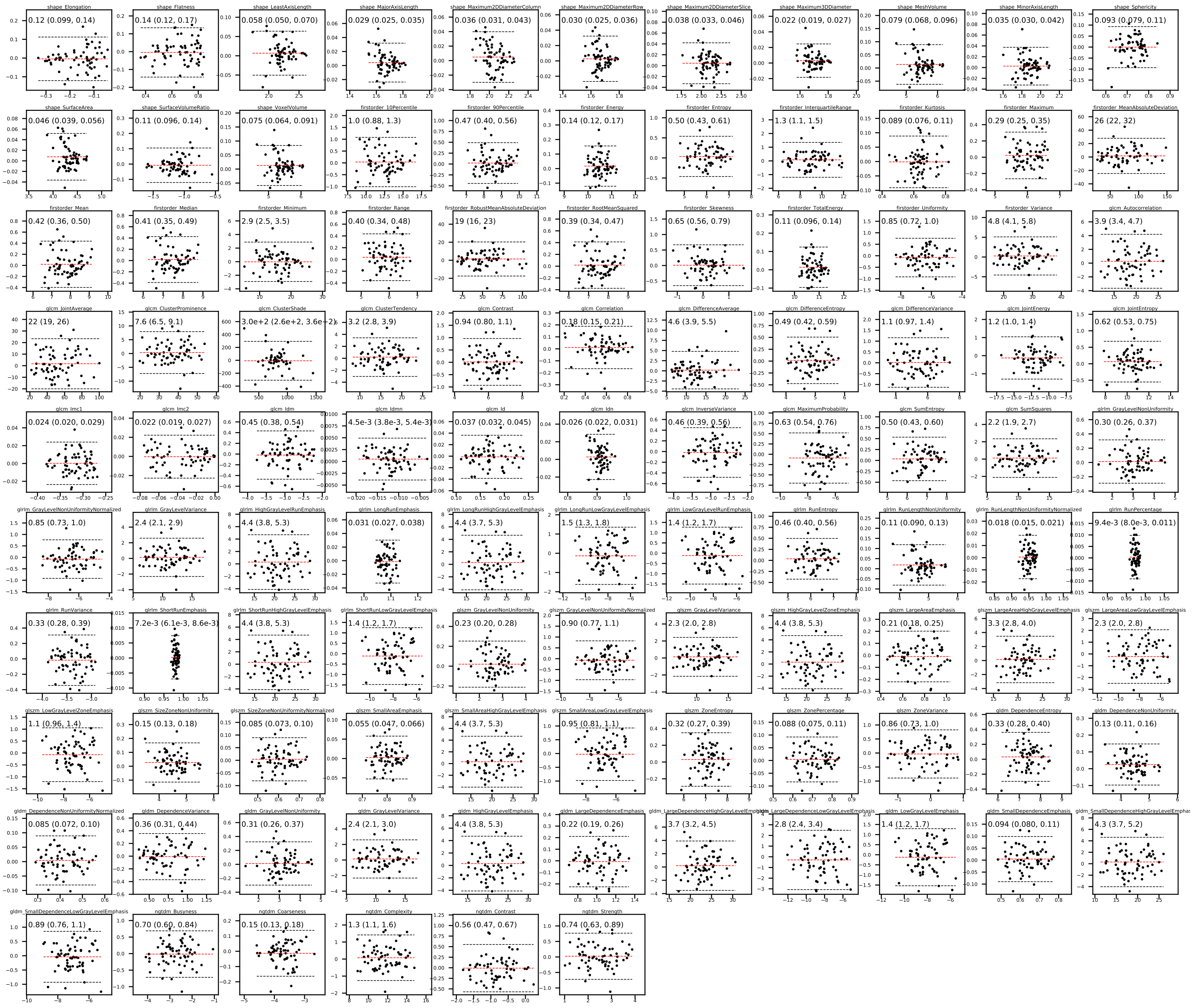

FIG. S13. Bland-Altman plots for T1W post-contrast images. For each feature, the difference between visits (visit 2 − visit 1) is plotted against the mean of the two visits. Red and black dashed lines indicate the mean difference and 95% limits of agreement, respectively. In each panel, the feature's RC and CIs are quoted.

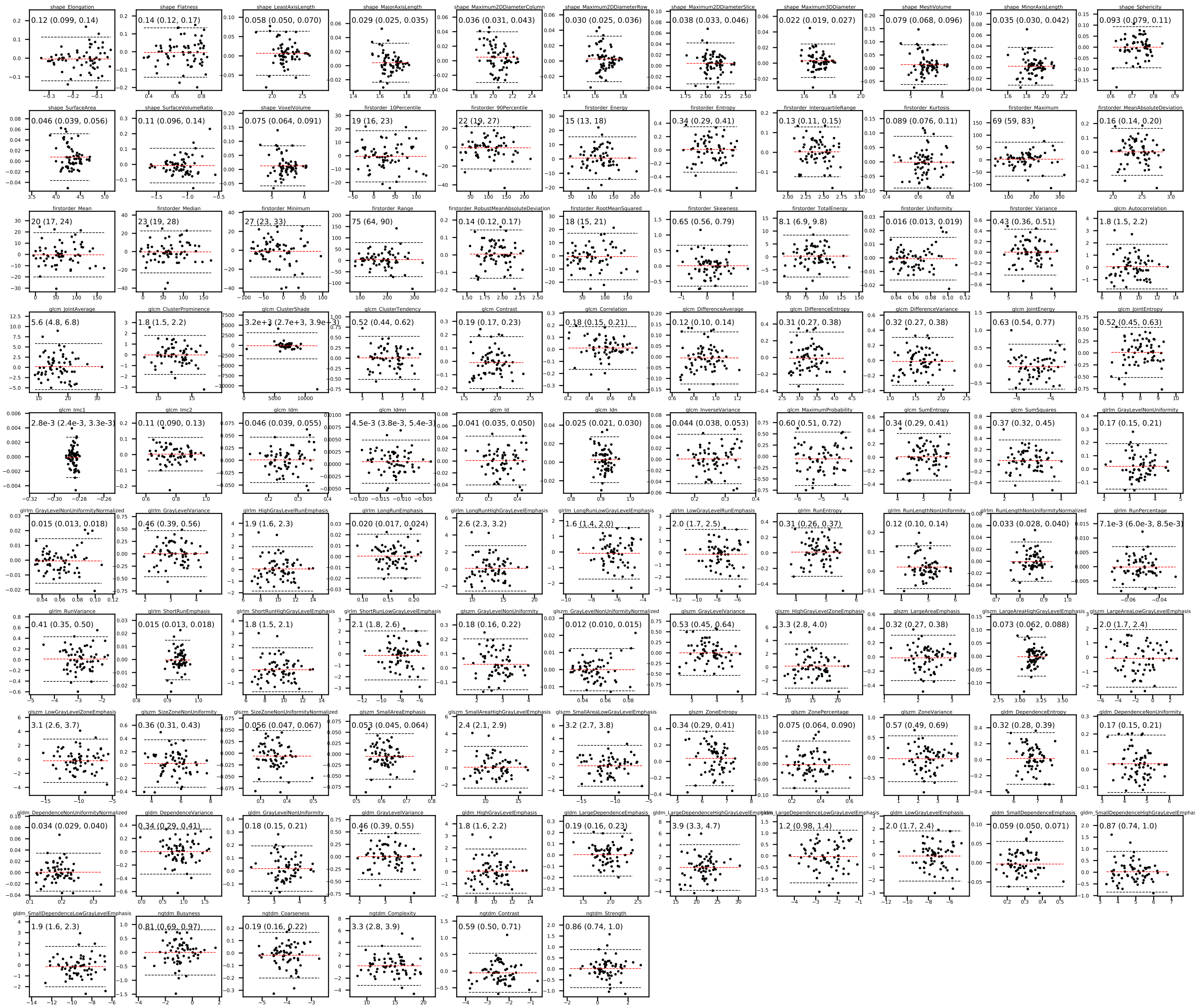

FIG. S14. Bland-Altman plots for normalised T1W post-contrast images. For each feature, the difference between visits (visit 2 − visit 1) is plotted against the mean of the two visits. Red and black dashed lines indicate the mean difference and 95% limits of agreement, respectively. In each panel, the feature's RC and CIs are quoted.

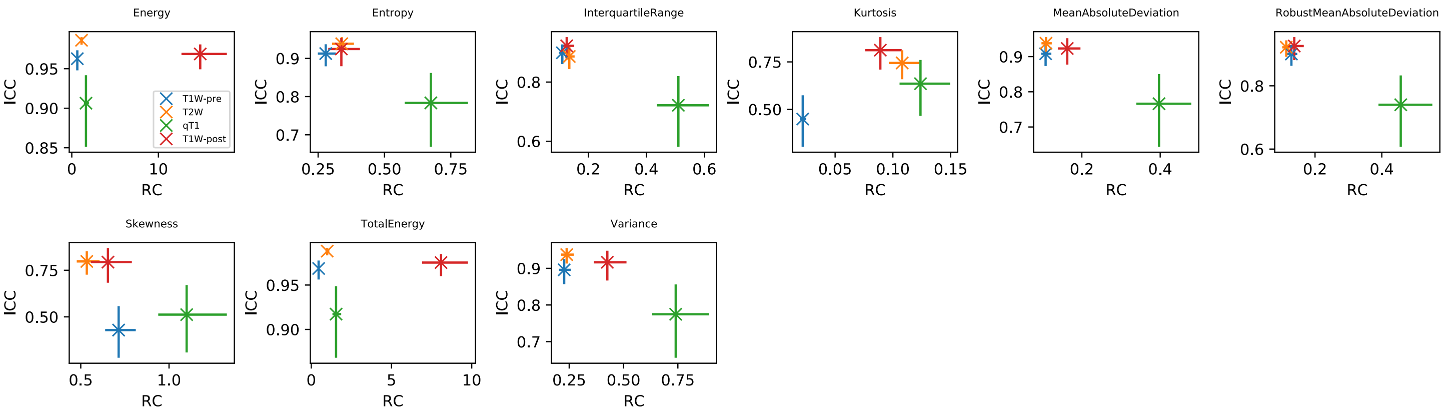

FIG. S15. RC plotted against ICC (point estimates and 95% CIs) for the four sequences (colours), for the subset of First Order features where Box-Cox transformations were consistently applied across the four sequences.

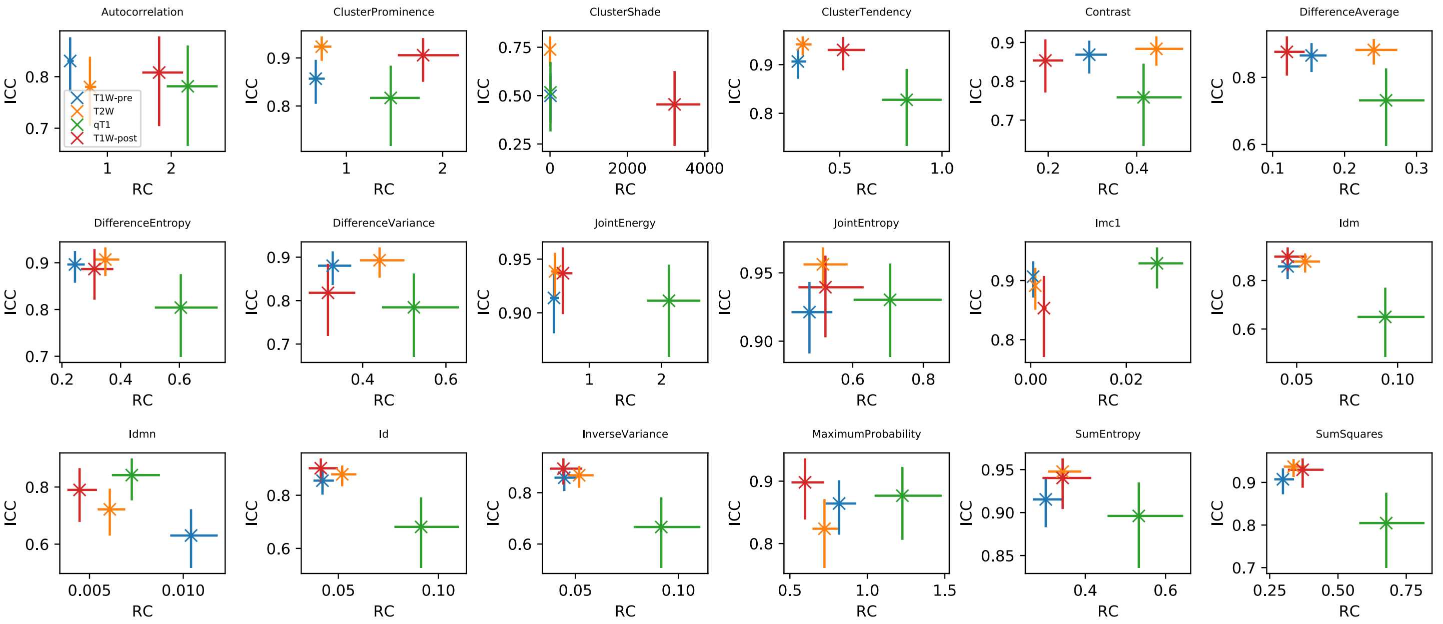

FIG. S16. RC plotted against ICC (point estimates and 95% CIs) for the four sequences (colours), for the subset of GLCM features where Box-Cox transformations were consistently applied across the four sequences.

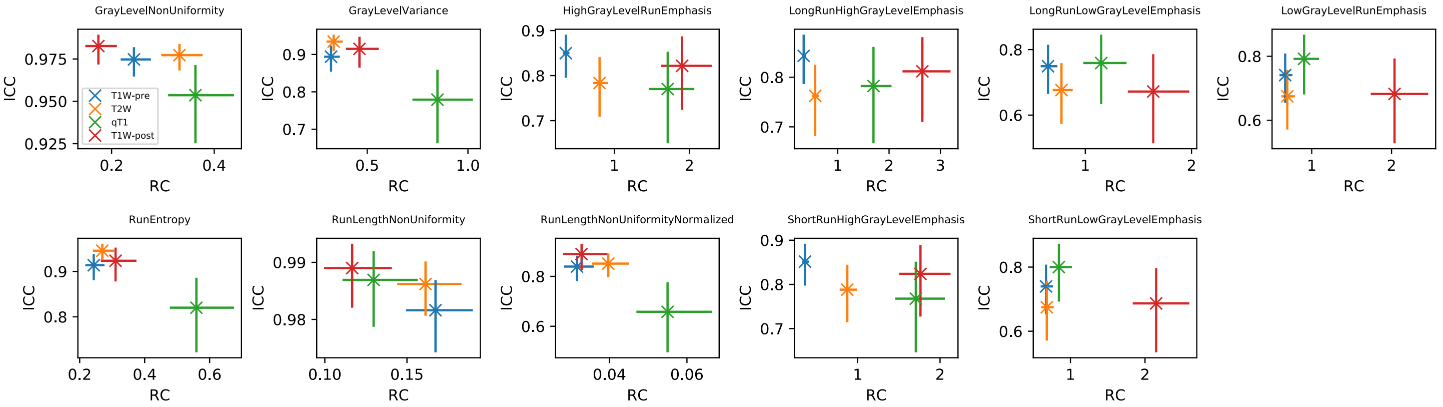

FIG. S17. RC plotted against ICC (point estimates and 95% CIs) for the four sequences (colours), for the subset of GLRLM features where Box-Cox transformations were consistently applied across the four sequences.

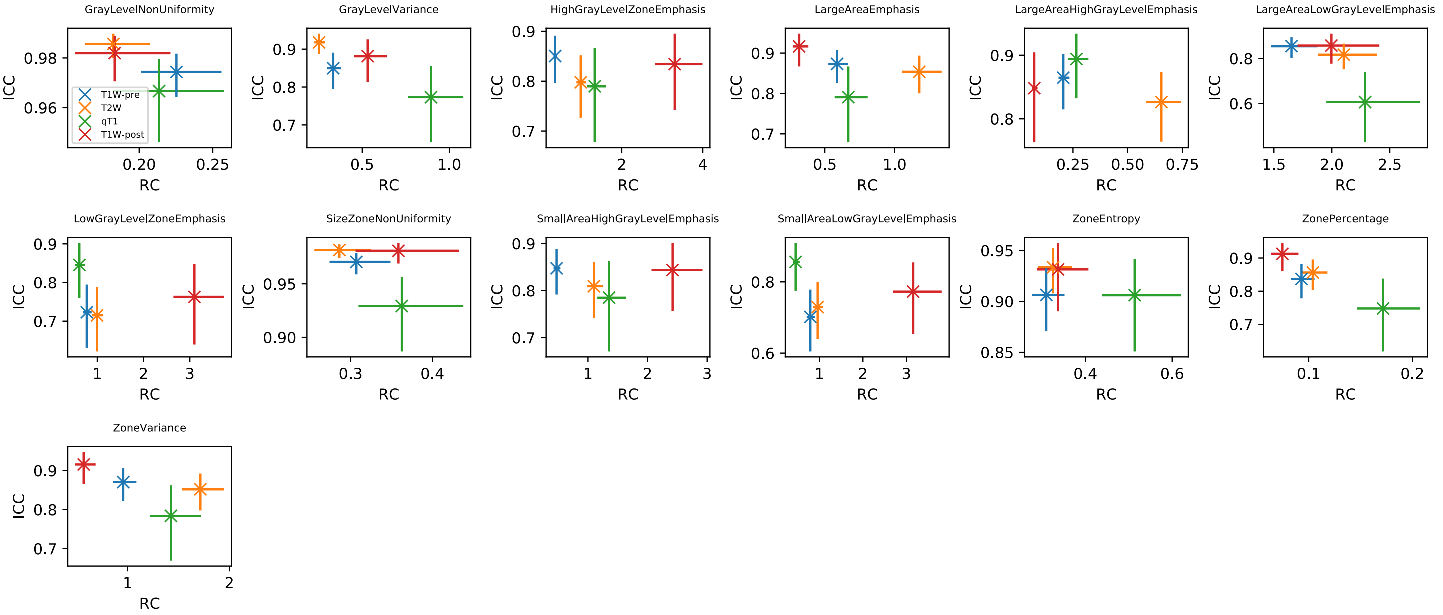

FIG. S18. RC plotted against ICC (point estimates and 95% CIs) for the four sequences (colours), for the subset of GLSZM features where Box-Cox transformations were consistently applied across the four sequences.

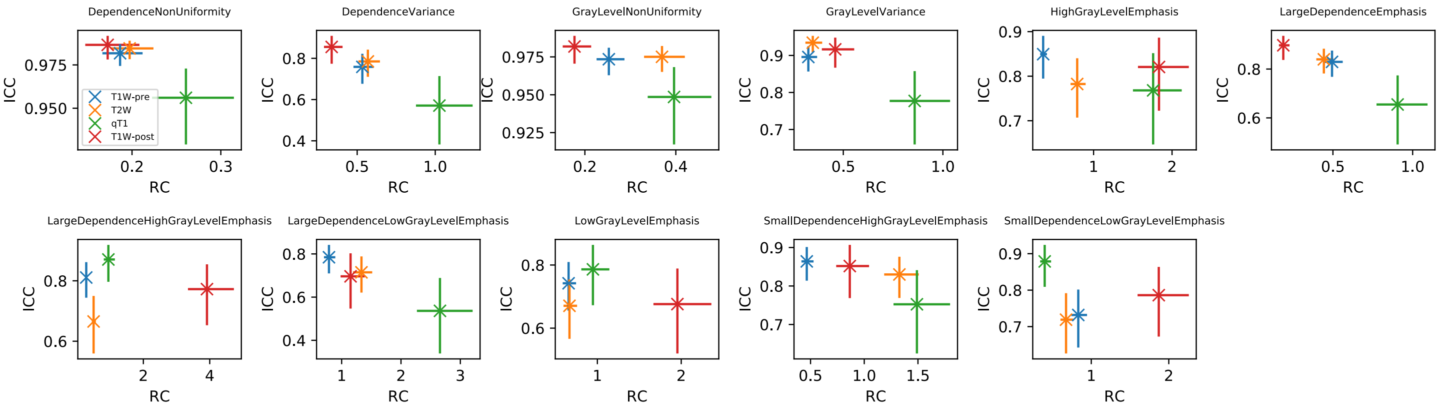

FIG. S19. RC plotted against ICC (point estimates and 95% CIs) for the four sequences (colours), for the subset of GLDM features where Box-Cox transformations were consistently applied across the four sequences.

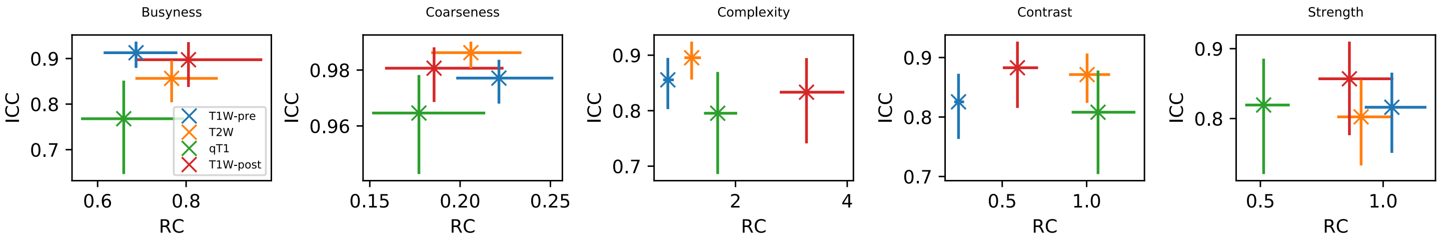

FIG. S20. RC plotted against ICC (point estimates and 95% CIs) for the four sequences (colours), for the subset of NGTDM features where Box-Cox transformations were consistently applied across the four sequences.

References

1. Weir, J.P. Quantifying test-retest reliability using the intraclass correlation coefficient and the SEM. *Journal of Strength and Conditioning Research* **2005**, *19*, 231–240.

2. Liljequist, D.; Elfving, B.; Roaldsen, K.S. Intraclass correlation – A discussion and demonstration of basic features. *PLoS ONE* **2019**, *14*, e0219854.

3. Bates, D.; Mächler, M.; Bolker, B.; Walker, S. Fitting Linear Mixed-Effects Models Using lme4. *Journal of Statistical Software* **2015**, *67*, 1–48.

4. R Core Team. *R: A Language and Environment for Statistical Computing.* R Foundation for Statistical Computing, Vienna, Austria, 2018.

5. McGraw, K.O.; Wong, S.P. Forming Inferences about Some Intraclass Correlation Coefficients. *Psychological Methods* **1996**, *1*, 30–46.

6. Stoffel, M.A.; Nakagawa, S.; Schielzeth, H. rptR: repeatability estimation and variance decomposition by generalized linear mixed-effects models. *Methods in Ecology and Evolution* **2017**, *8*, 1639–1644.

7. Barnhart, H.X.; Barboriak, D.P. Applications of the repeatability of quantitative imaging biomarkers: A review of statistical analysis of repeat data sets. *Translational Oncology* **2009**, *2*, 231–235.
