# Supplementary figures and images for: Image Contrast, Image Pre-Processing, and T1 Mapping Affect MRI Radiomic Feature Repeatability in Patients with Colorectal Cancer Liver Metastases
Source: Cancers (Basel). 2021 Jan 11;13(2):240. doi: 10.3390/cancers13020240 (PMC7826650; doi:10.3390/cancers13020240)

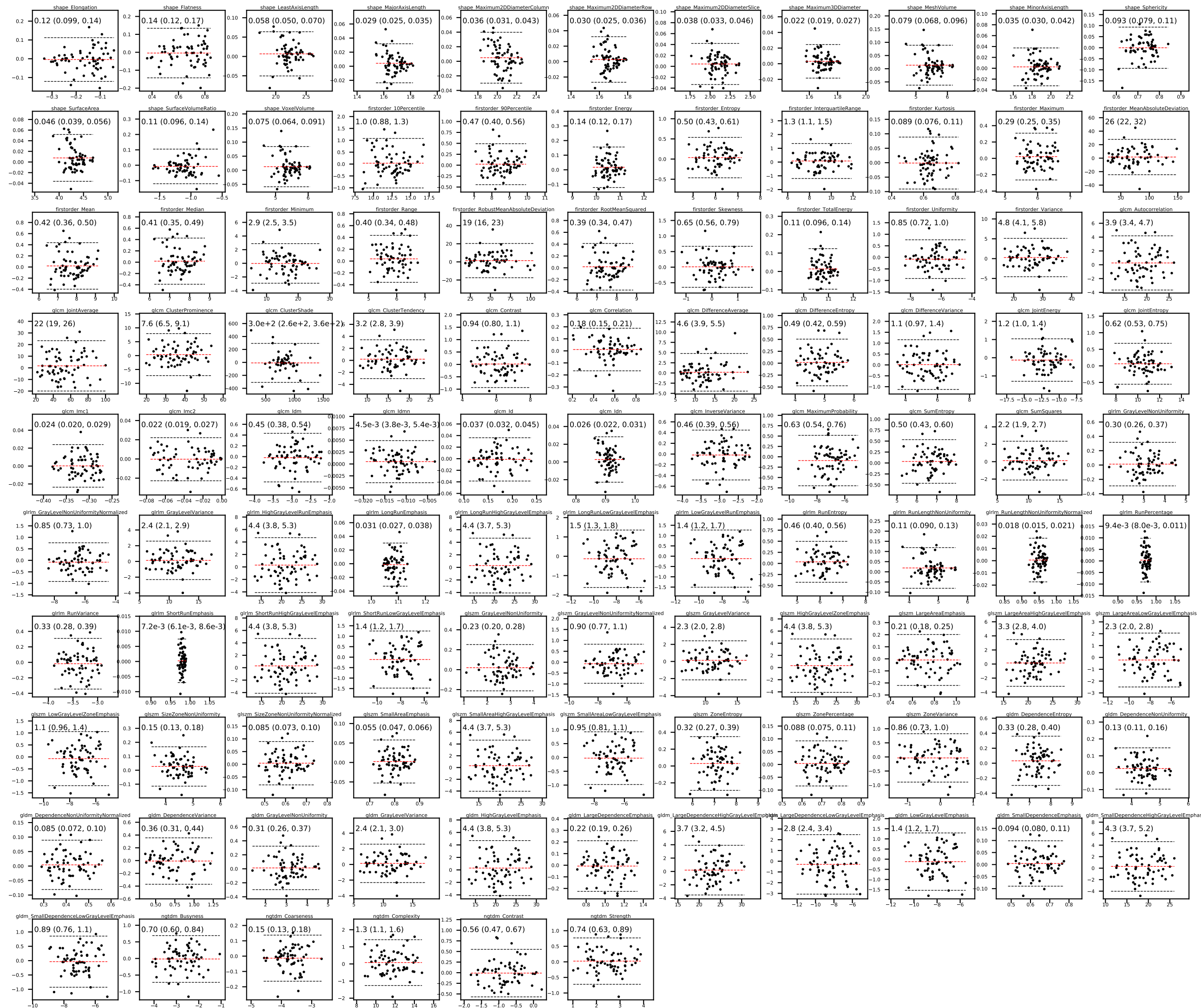

Supplement: Supplementary file 1 [file cancers-13-00240-s001.zip › MDPI_template_Cancers_radiomics_supporting_information_submission_071220/figures/supporting_bland-altman_BoxCox_105features_T1_postcon_Params_normalise_false_bw5.pdf]

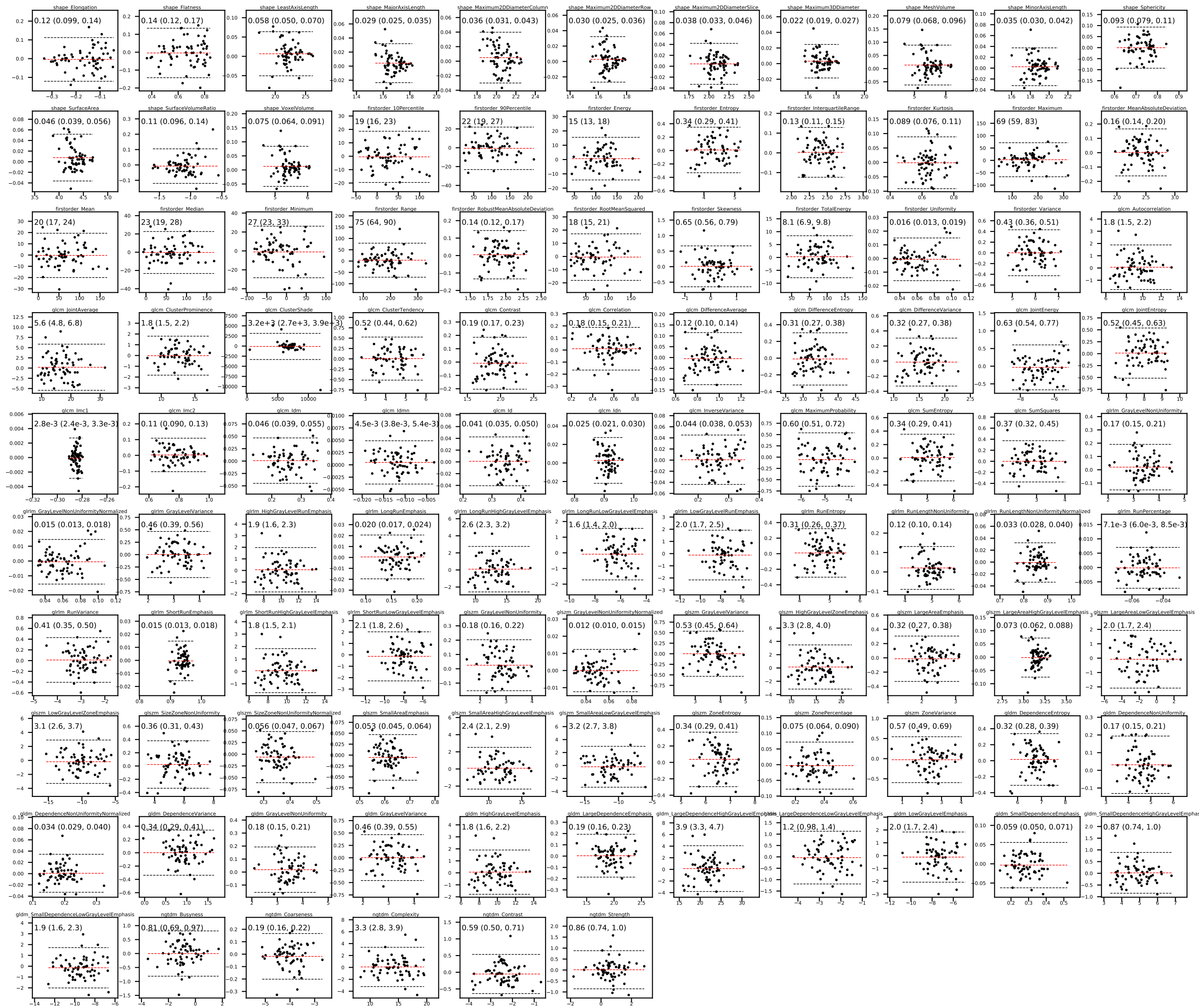

Supplement: Supplementary file 1 [file cancers-13-00240-s001.zip › MDPI_template_Cancers_radiomics_supporting_information_submission_071220/figures/supporting_bland-altman_BoxCox_105features_T1_postcon_Params_normalise_true_scale100_shift0_bw5.pdf]

(a)

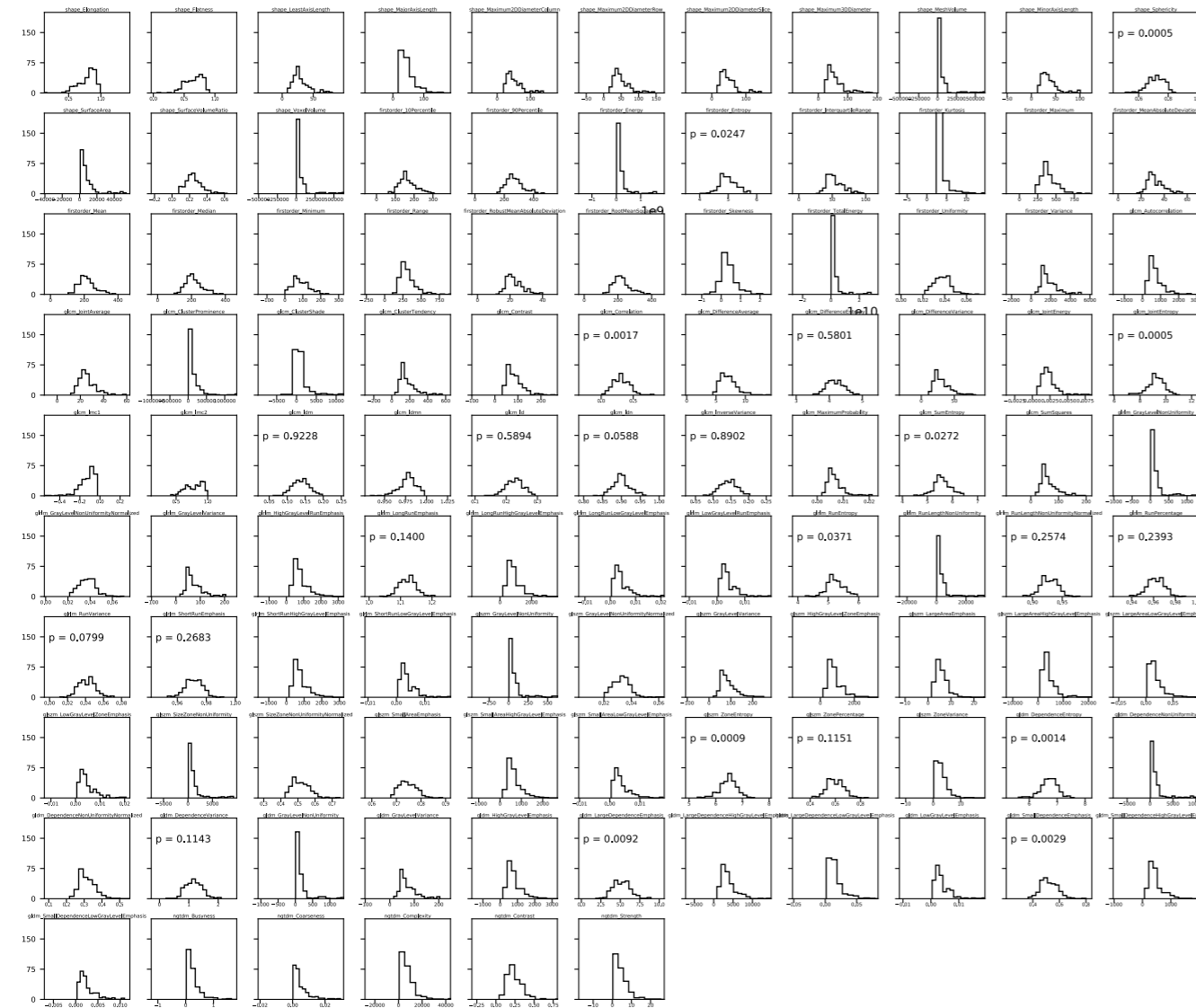

(b)

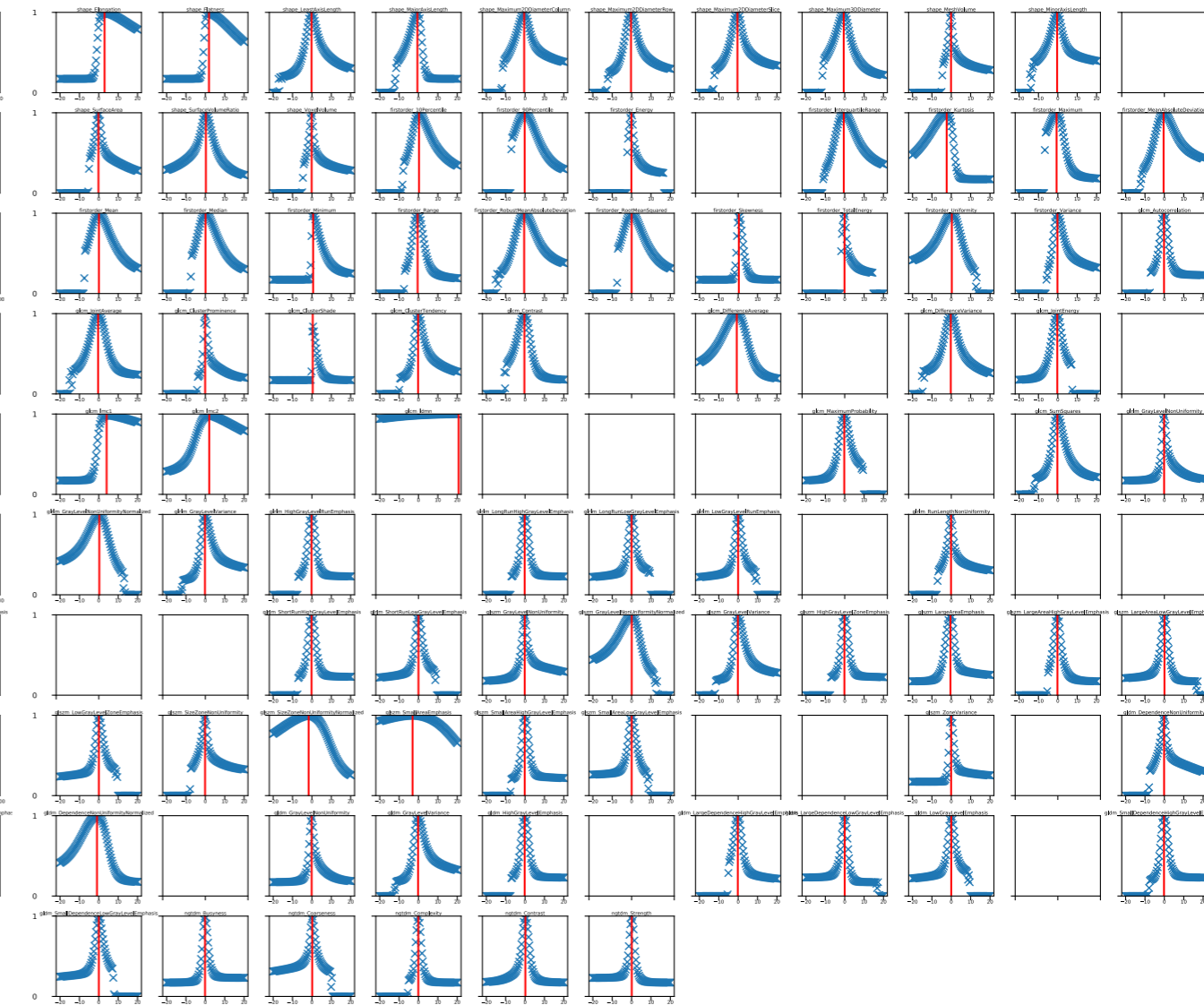

(c)

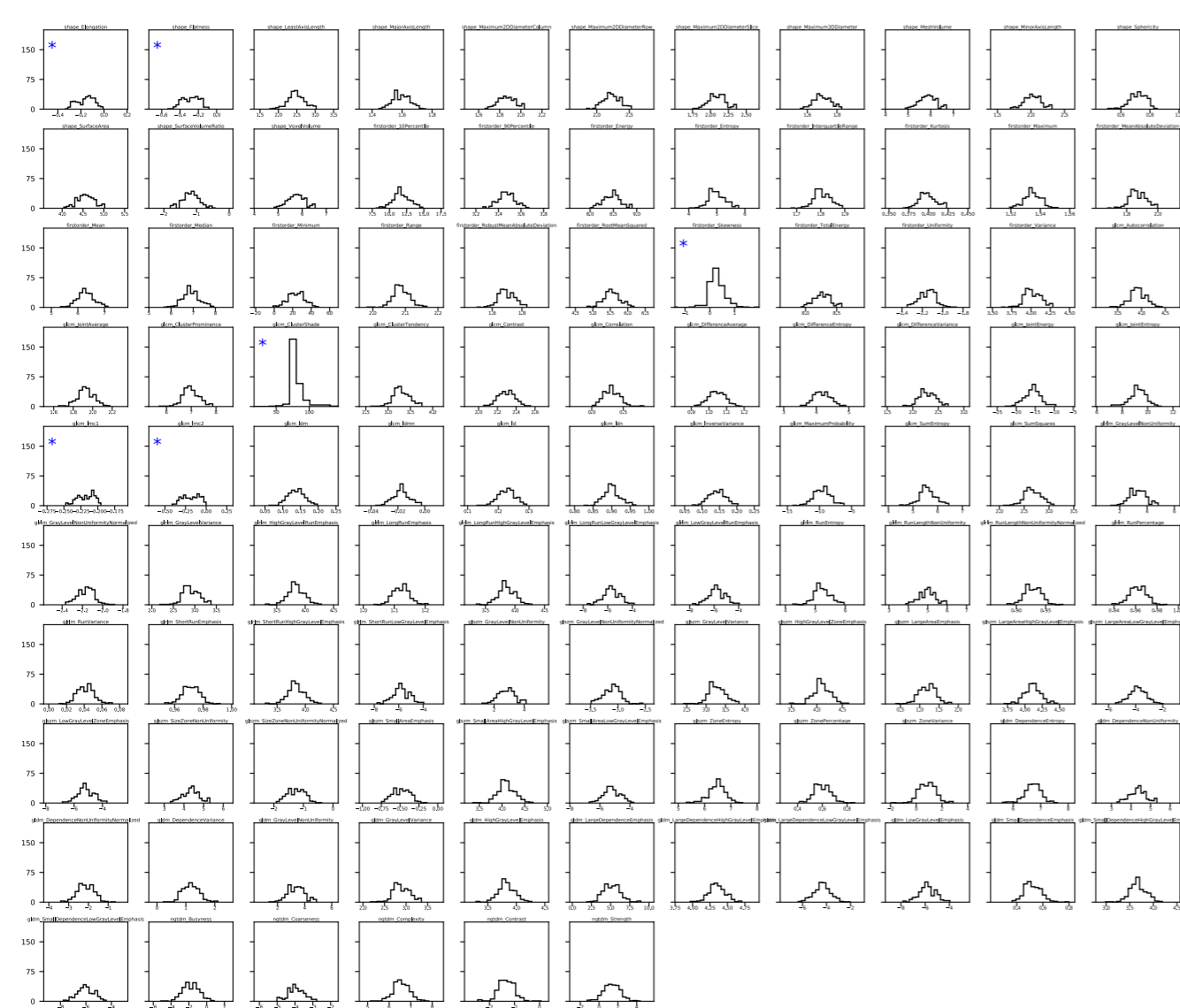

(d)

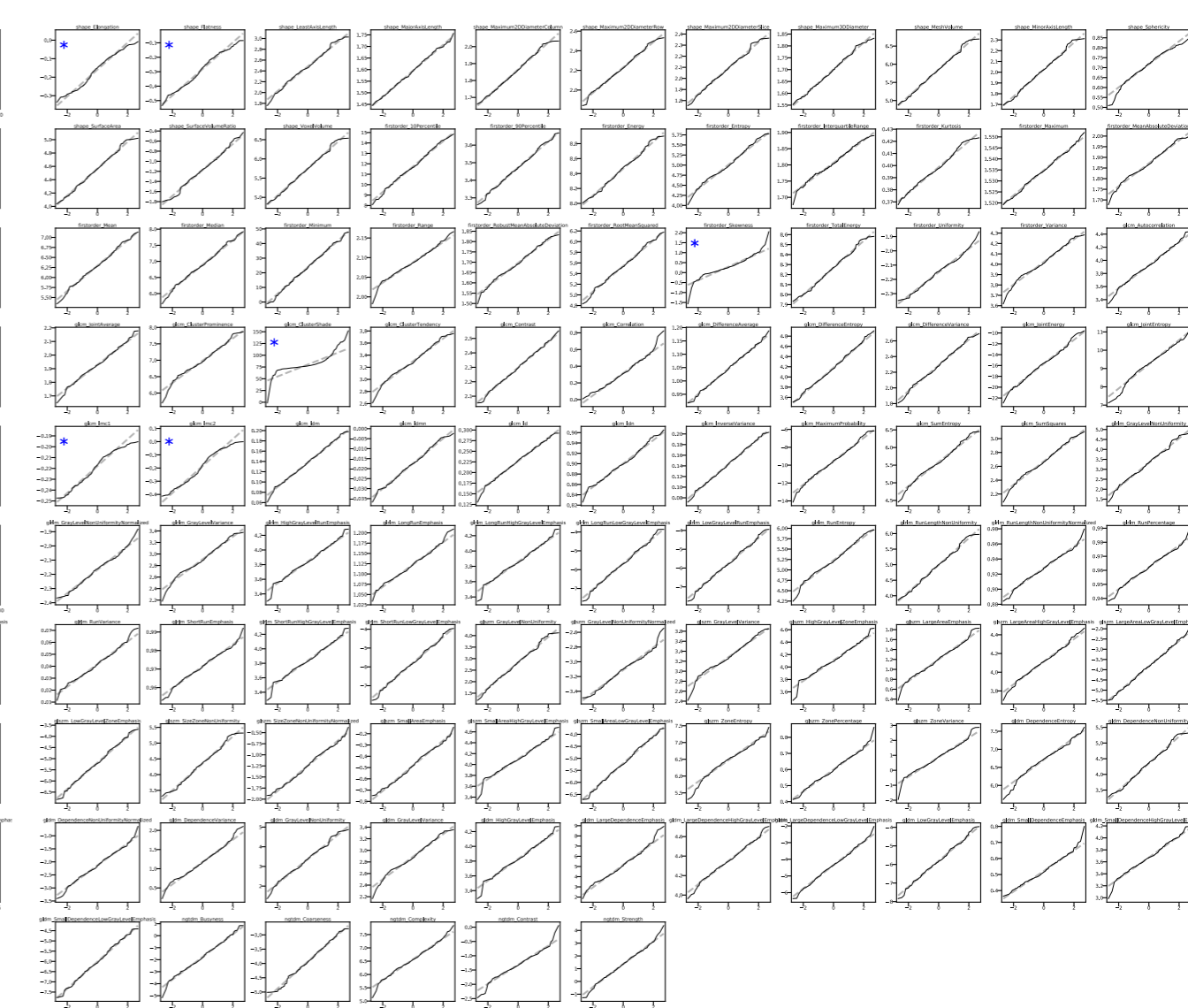

Supplement: Supplementary file 1 [file cancers-13-00240-s001.zip › MDPI_template_Cancers_radiomics_supporting_information_submission_071220/figures/supporting_feature_hists_box_cox_qq_t1w.pdf]

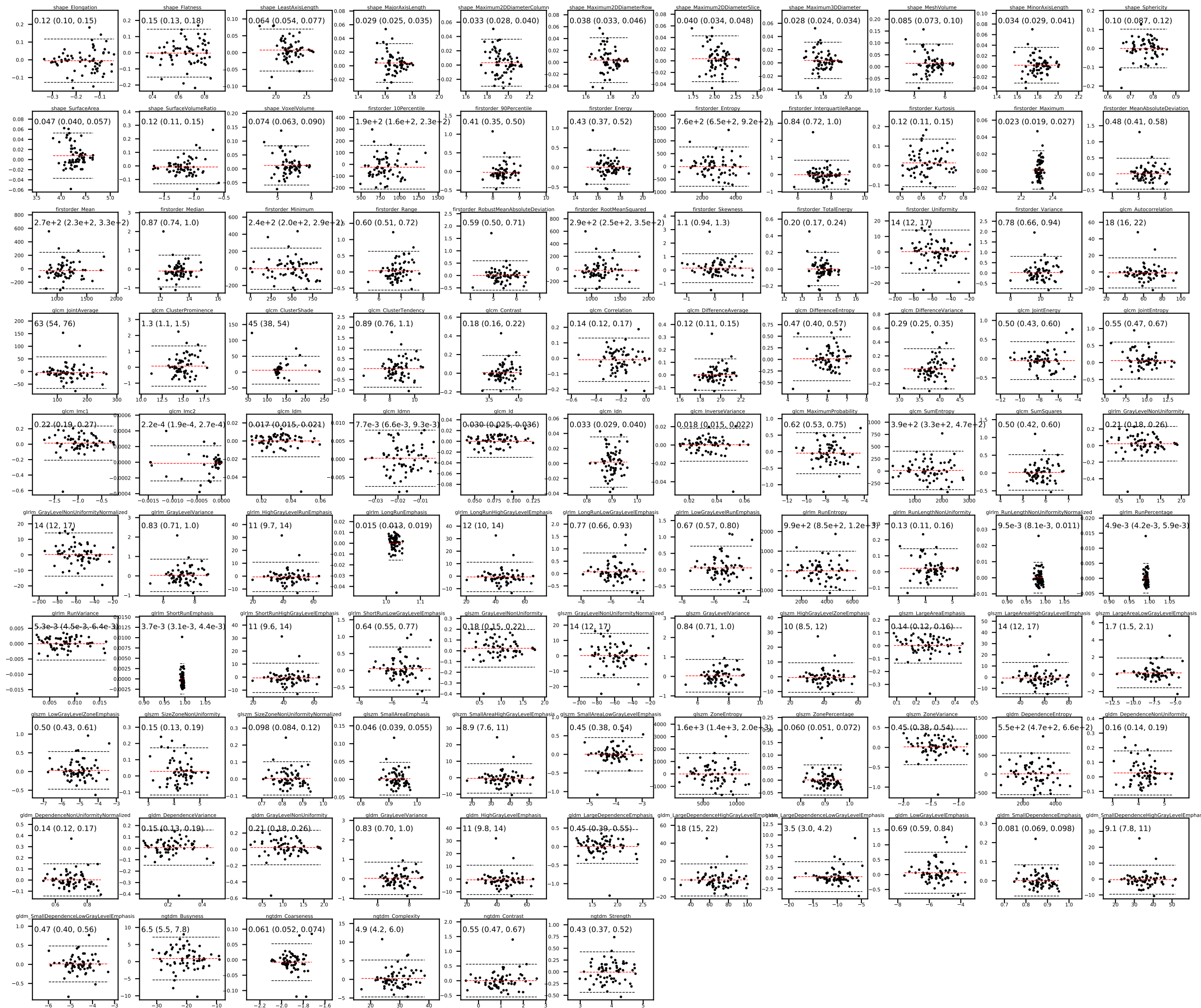

Supplement: Supplementary file 1 [file cancers-13-00240-s001.zip › MDPI_template_Cancers_radiomics_supporting_information_submission_071220/figures/supporting_bland-altman_BoxCox_105features_T1_map_Params_normalise_false_bw5.pdf]

**(a)**

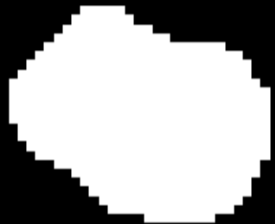

T1W pre-contrast mask  
256 x 256

**(b)**

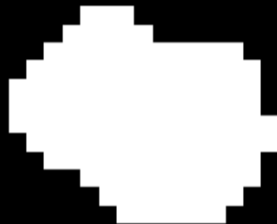

qT1 map mask  
128 x 128

**(c)**

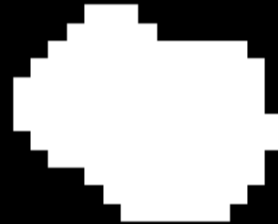

T1 pre-contrast subset mask  
256 x 256

Supplement: Supplementary file 1 [file cancers-13-00240-s001.zip › MDPI_template_Cancers_radiomics_supporting_information_submission_071220/figures/supporting_masks_t1wpre_qt1_t1wpre-subset.pdf]

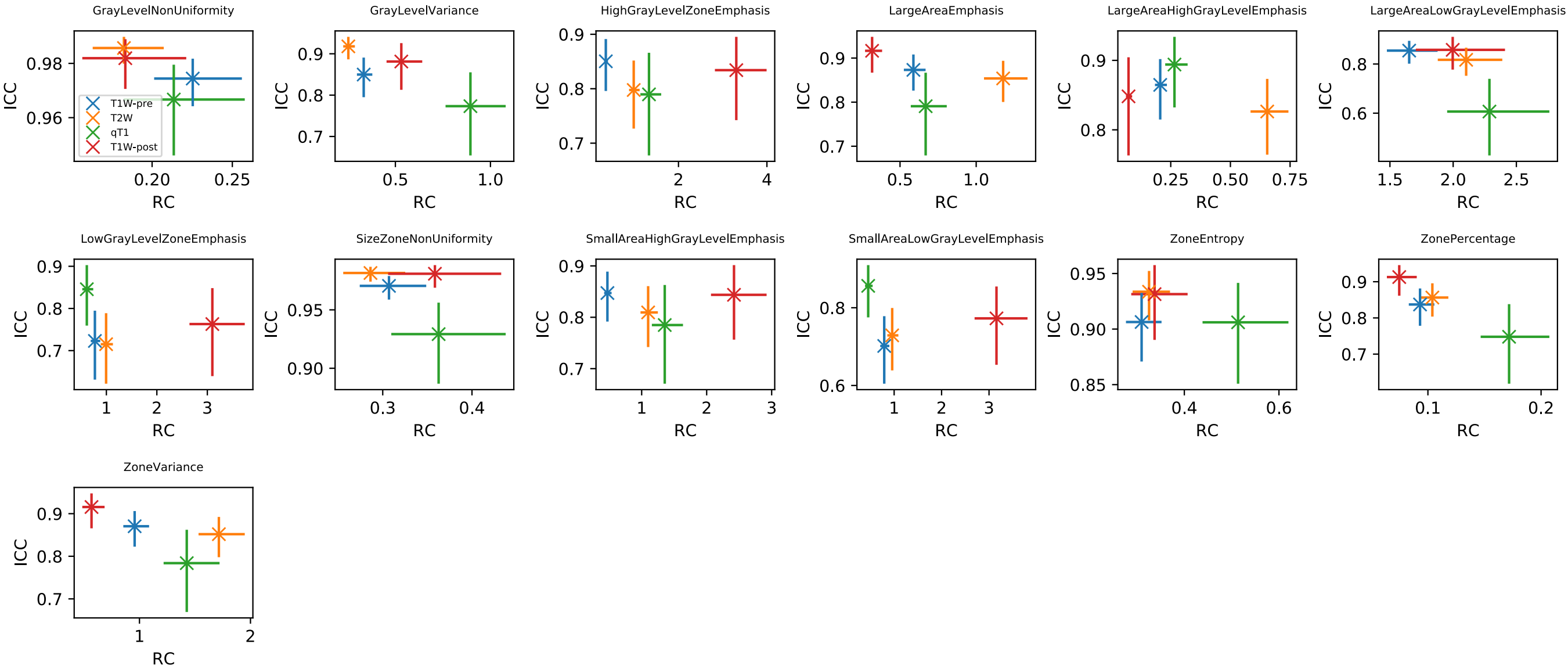

Supplement: Supplementary file 1 [file cancers-13-00240-s001.zip › MDPI_template_Cancers_radiomics_supporting_information_submission_071220/figures/supporting_cf_icc_rc_glszm.pdf]

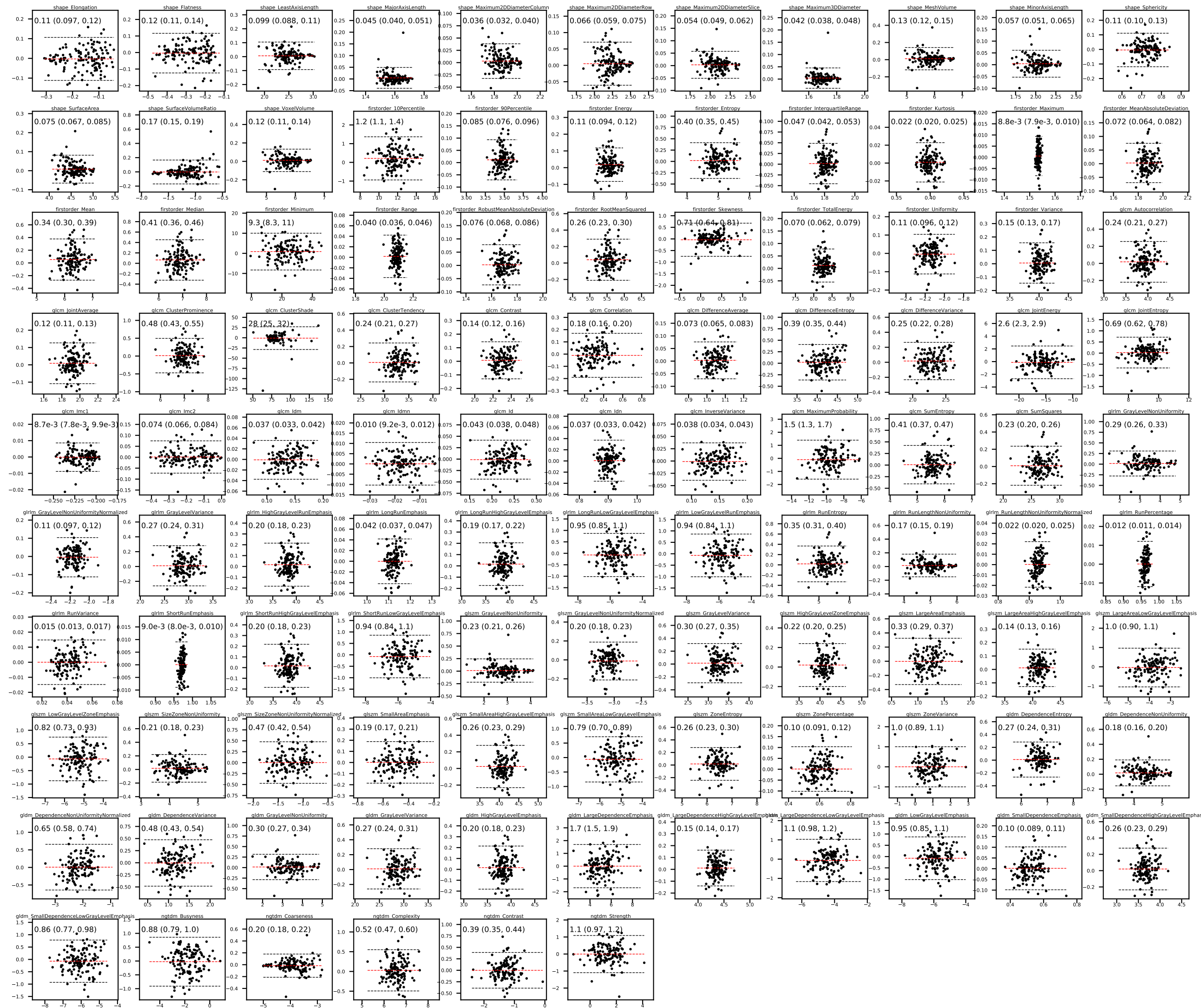

Supplement: Supplementary file 1 [file cancers-13-00240-s001.zip › MDPI_template_Cancers_radiomics_supporting_information_submission_071220/figures/supporting_bland-altman_BoxCox_105features_T1_precon_Params_normalise_false_bw5.pdf]

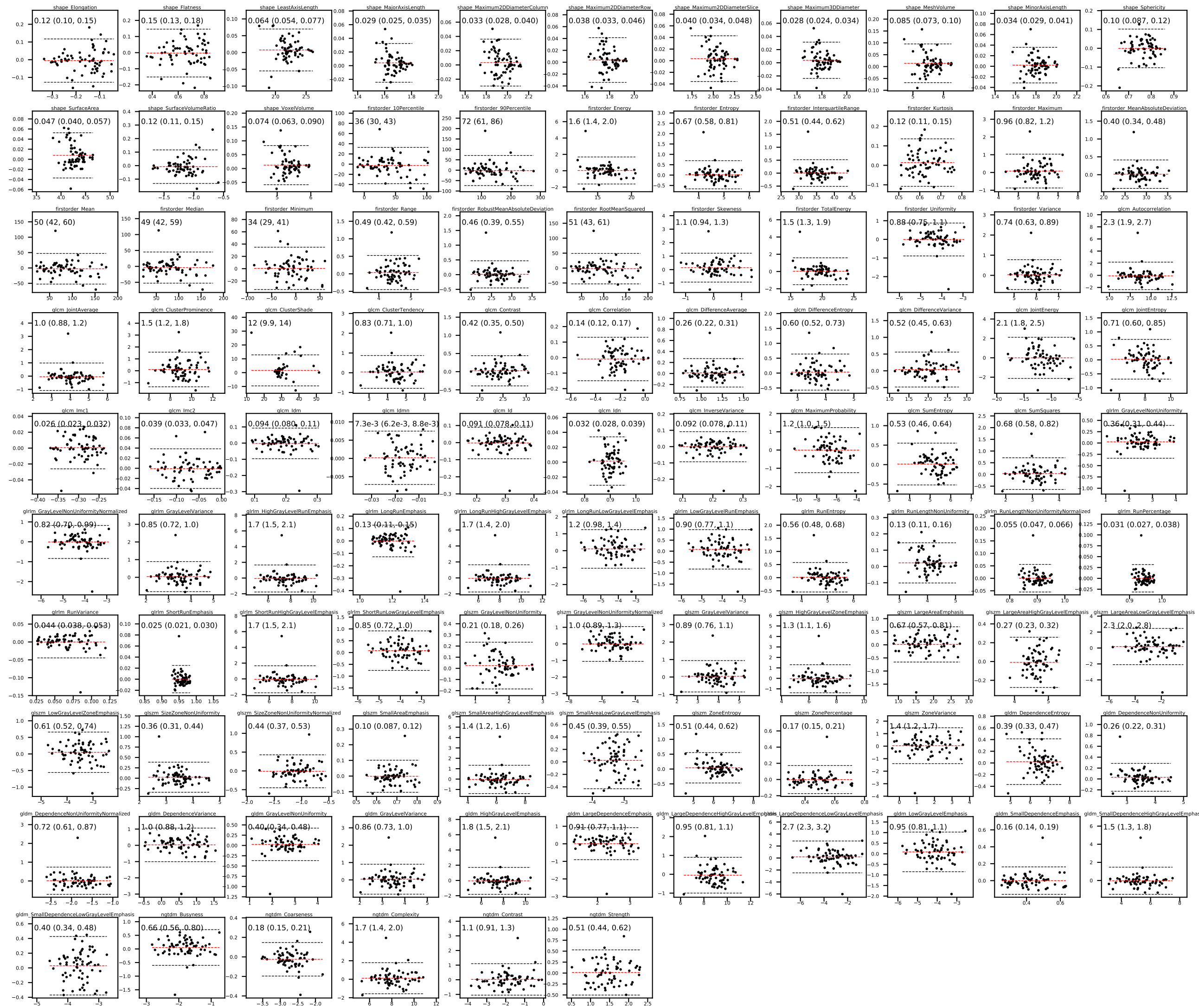

Supplement: Supplementary file 1 [file cancers-13-00240-s001.zip › MDPI_template_Cancers_radiomics_supporting_information_submission_071220/figures/supporting_bland-altman_BoxCox_105features_T1_map_Params_normalise_true_scale100_shift0_bw5.pdf]

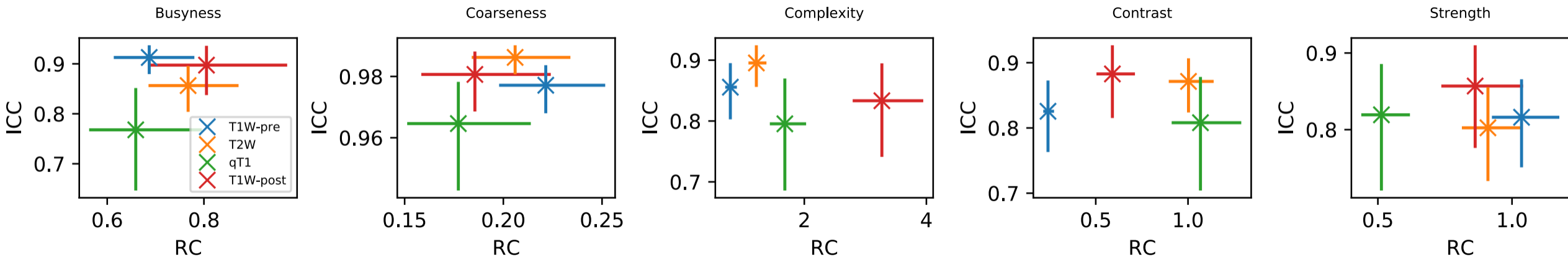

Supplement: Supplementary file 1 [file cancers-13-00240-s001.zip › MDPI_template_Cancers_radiomics_supporting_information_submission_071220/figures/supporting_cf_icc_rc_ngtdm.pdf]

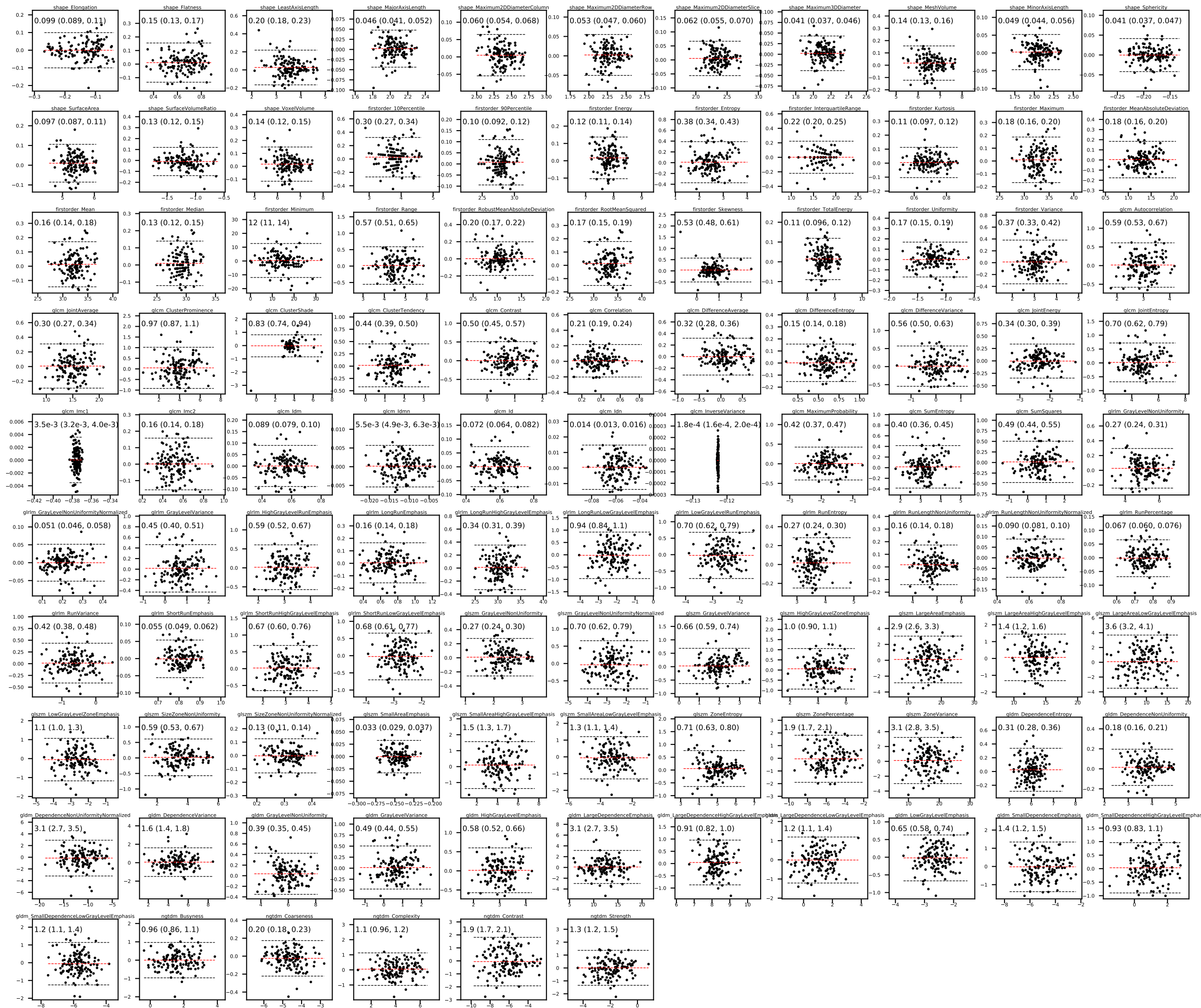

Supplement: Supplementary file 1 [file cancers-13-00240-s001.zip › MDPI_template_Cancers_radiomics_supporting_information_submission_071220/figures/supporting_bland-altman_BoxCox_105features_T2_Params_normalise_false_bw5.pdf]

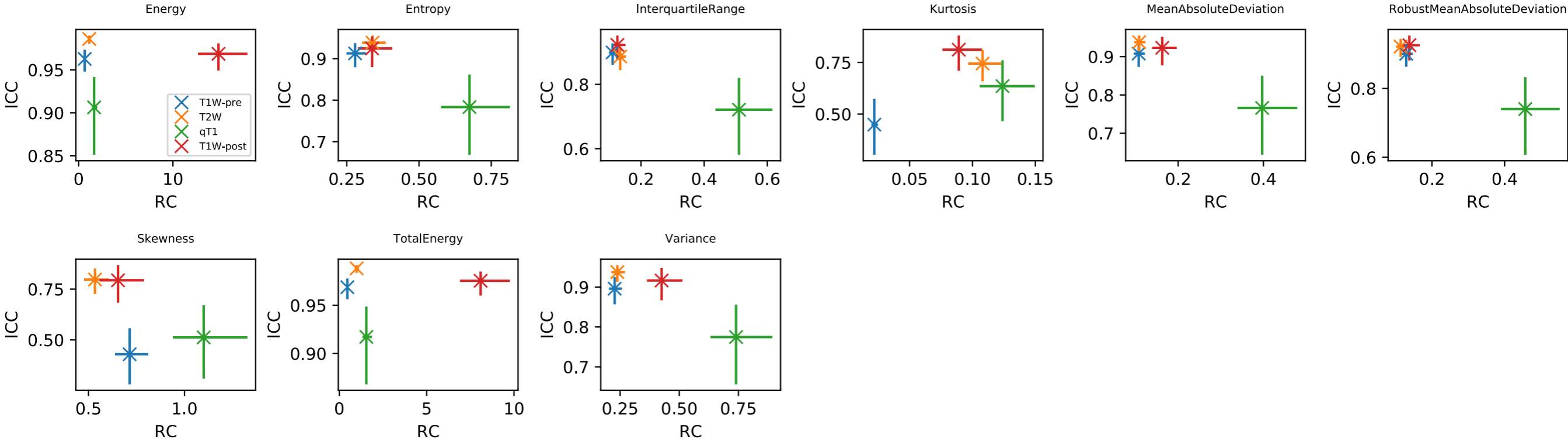

Supplement: Supplementary file 1 [file cancers-13-00240-s001.zip › MDPI_template_Cancers_radiomics_supporting_information_submission_071220/figures/supporting_cf_icc_rc_firstorder.pdf]

**(a)**

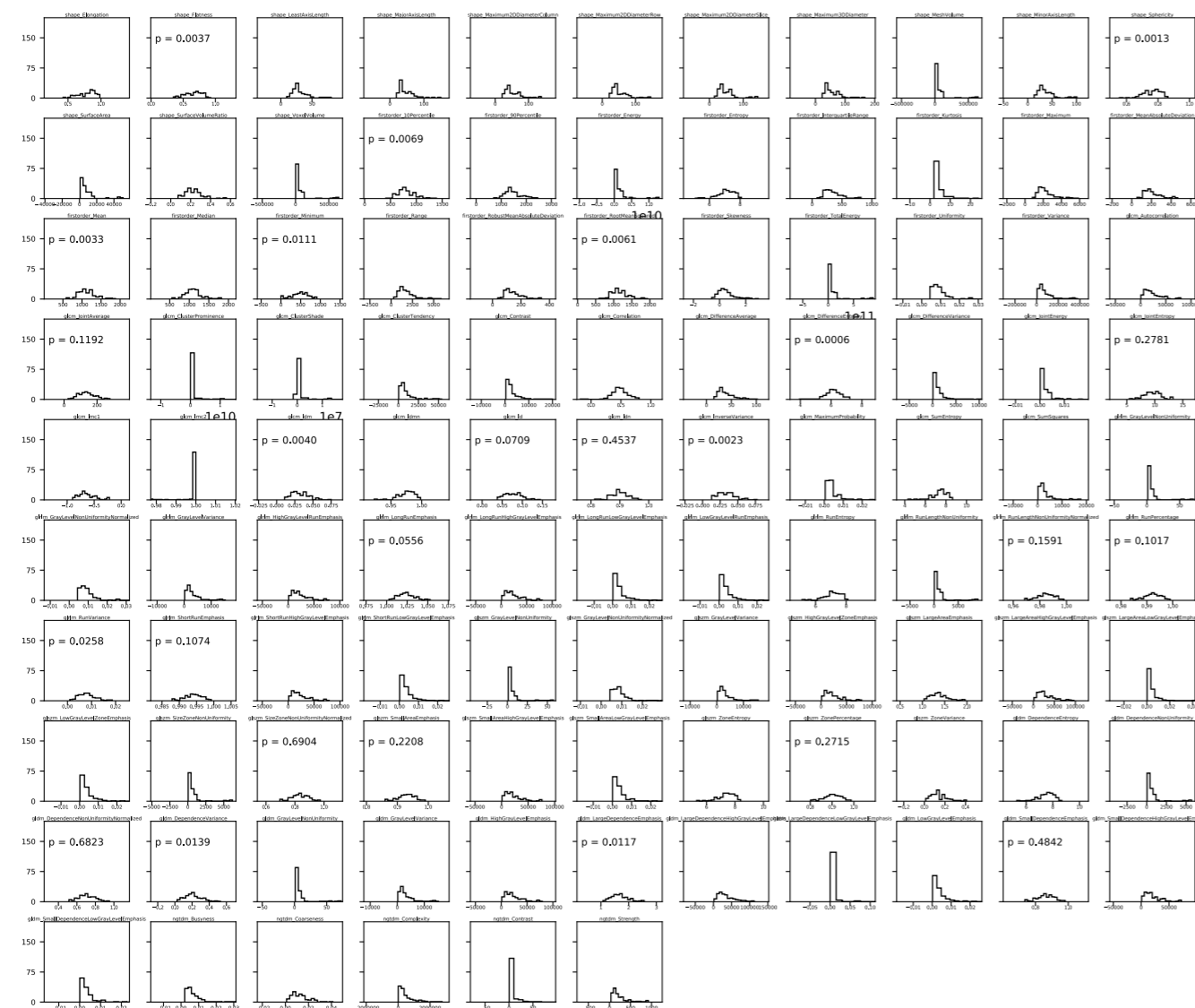

**(b)**

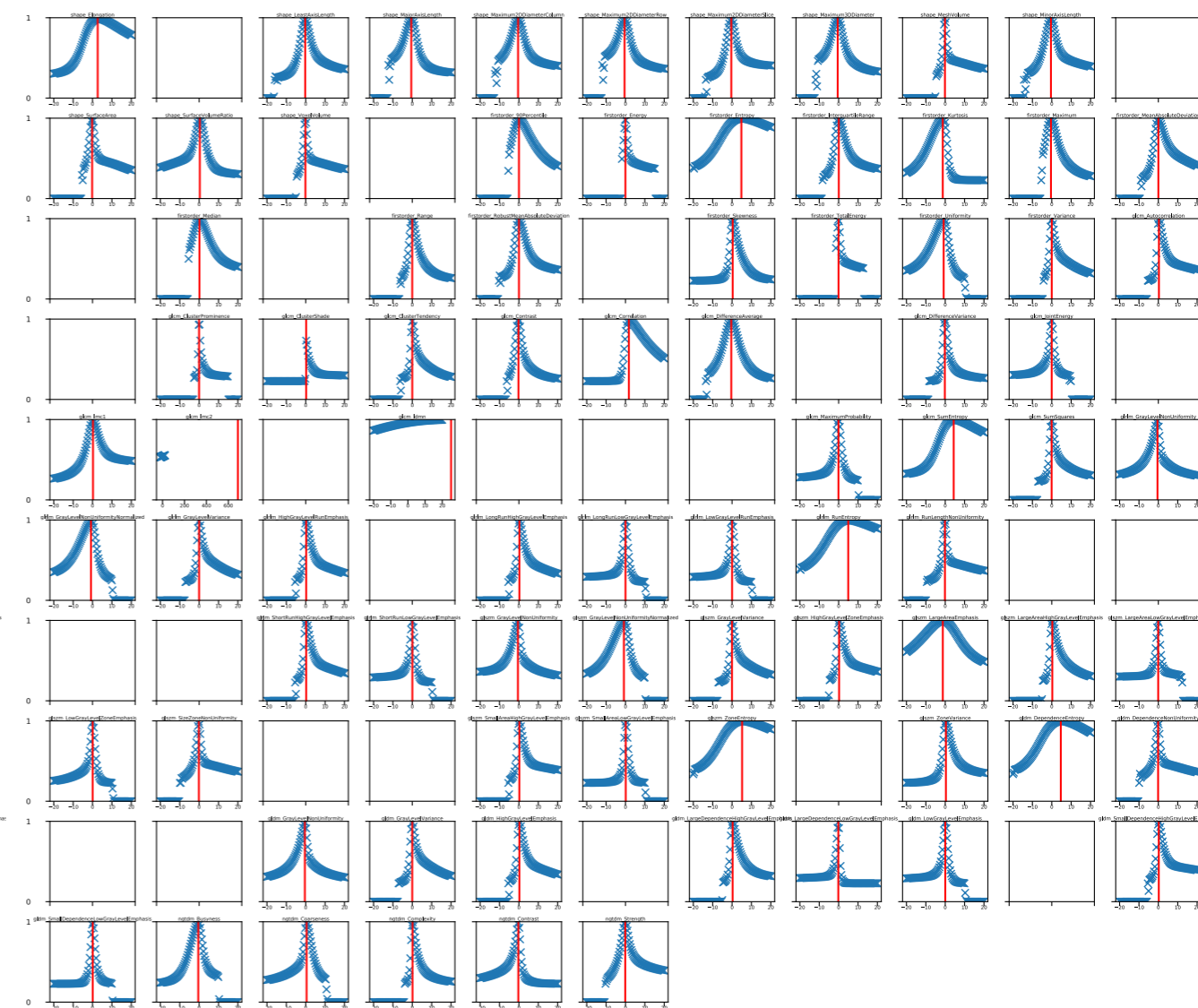

**(c)**

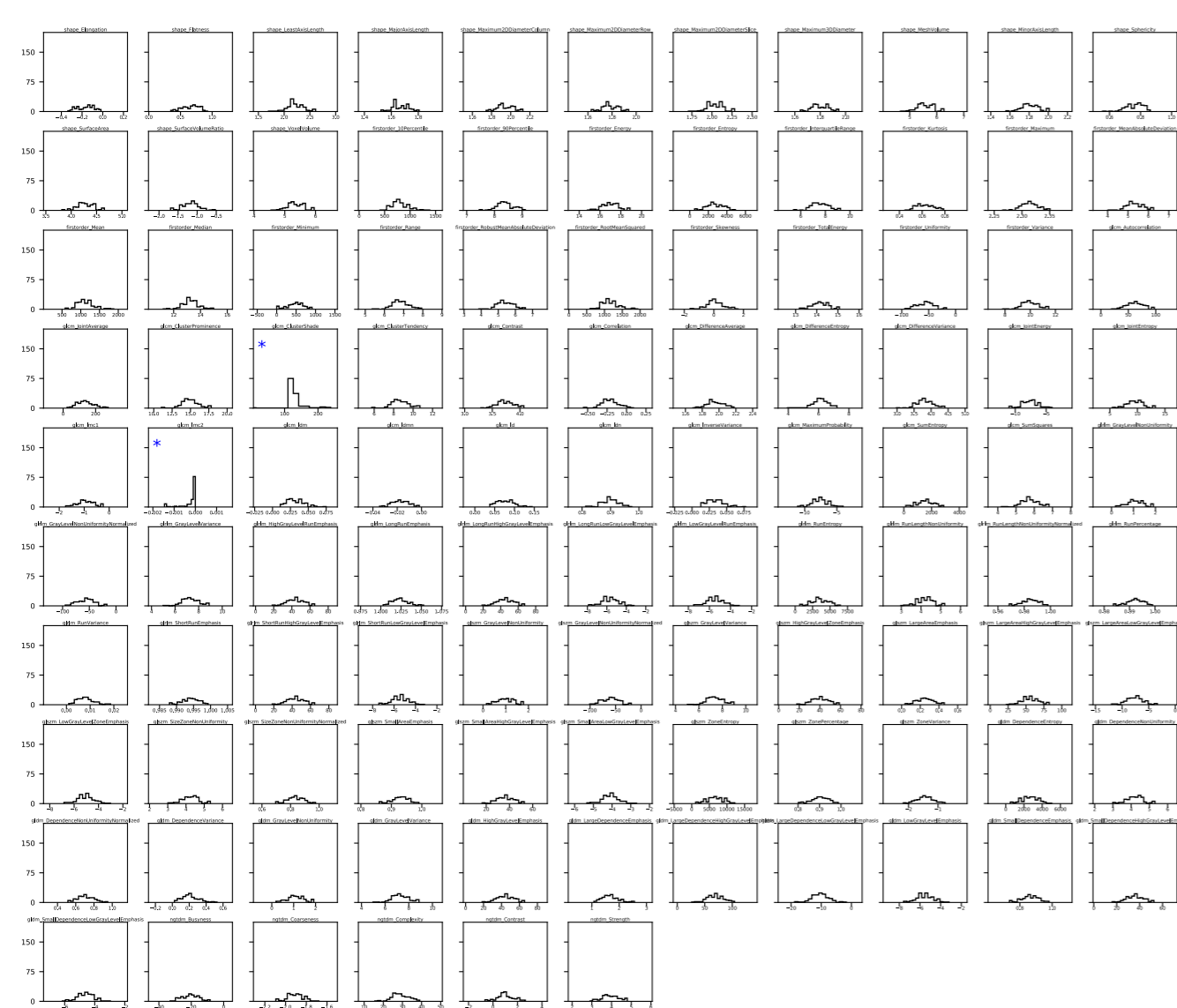

**(d)**

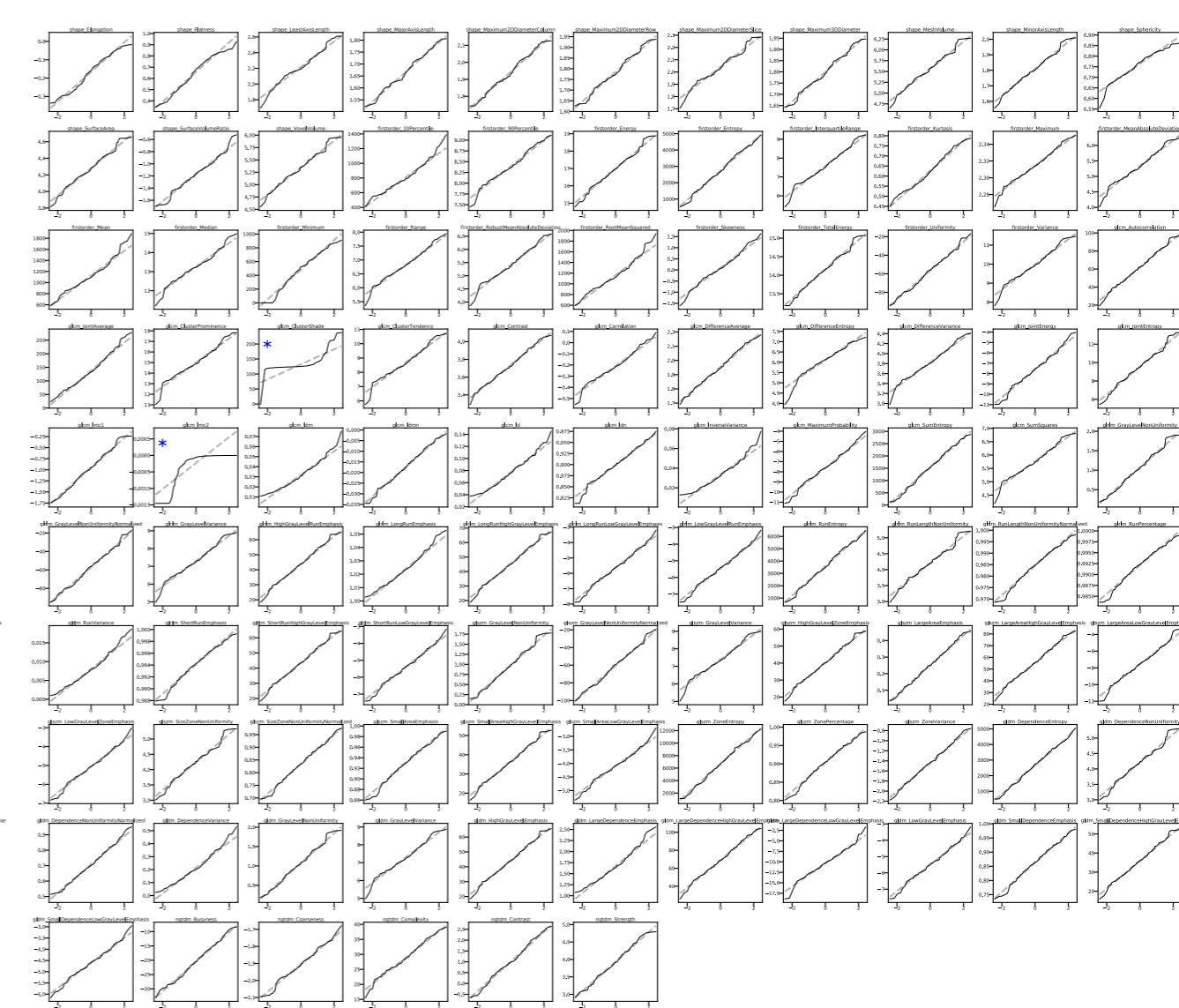

Supplement: Supplementary file 1 [file cancers-13-00240-s001.zip › MDPI_template_Cancers_radiomics_supporting_information_submission_071220/figures/supporting_feature_hists_box_cox_qq_qt1map.pdf]

**(a)**

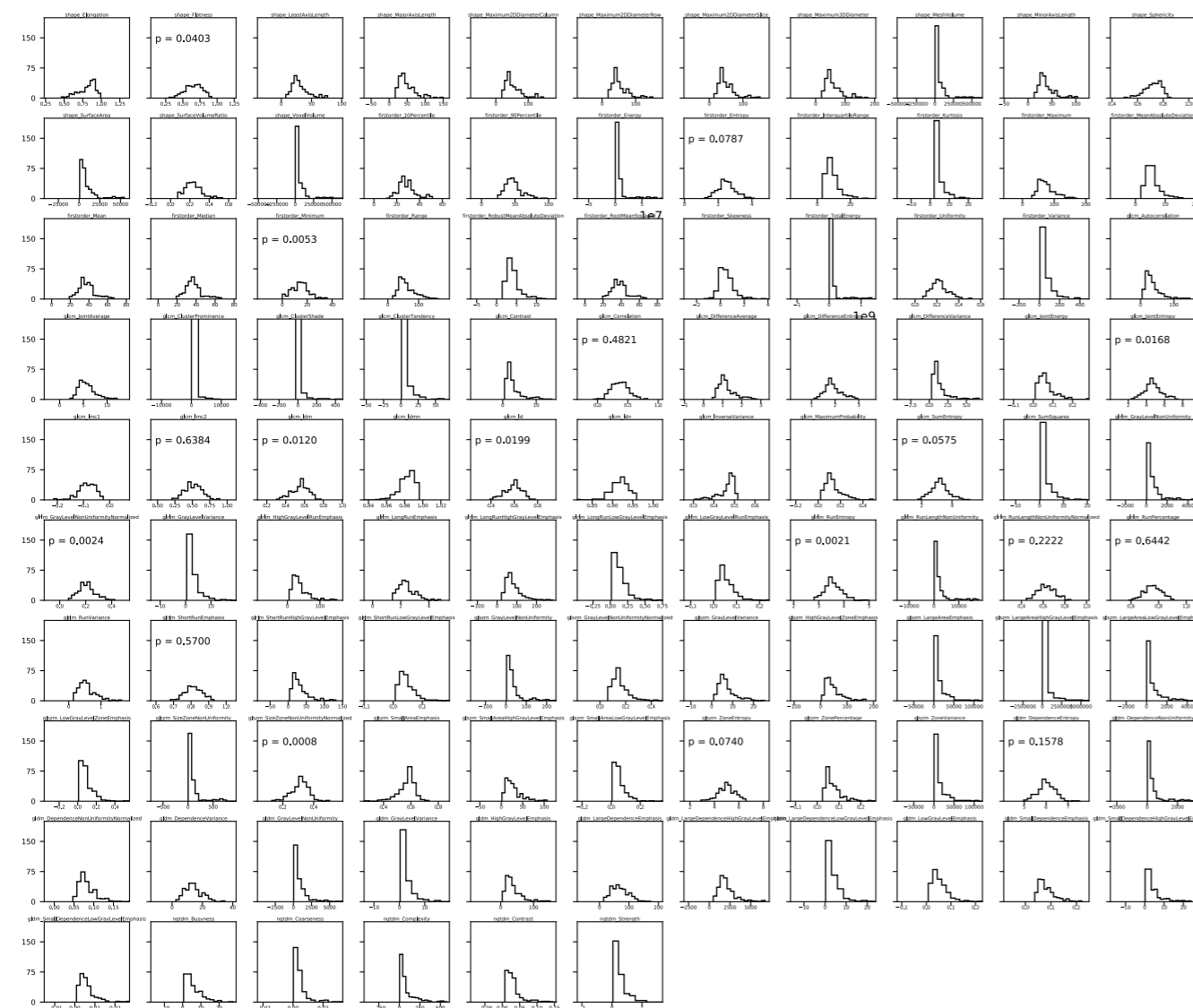

**(c)**

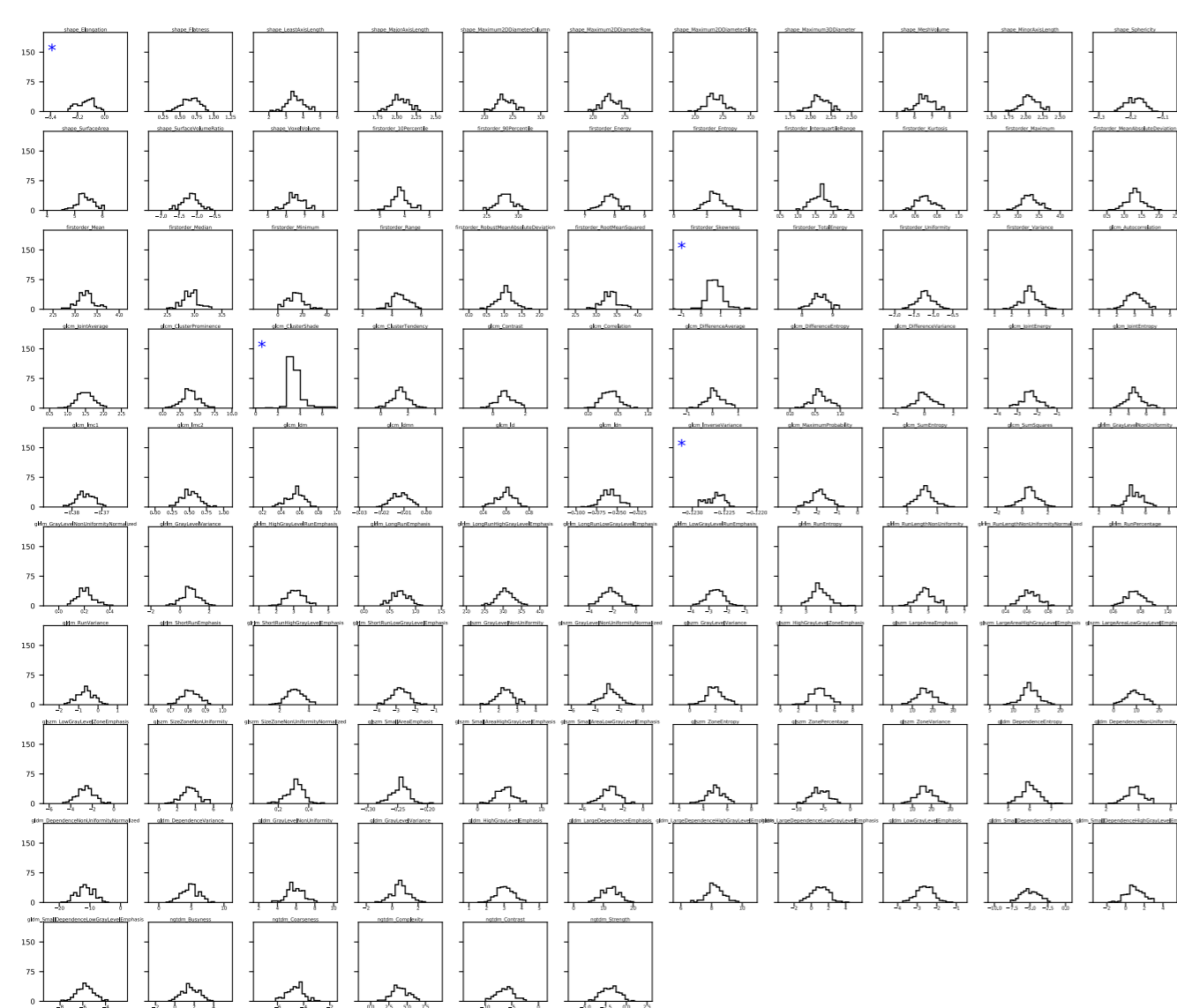

(b)

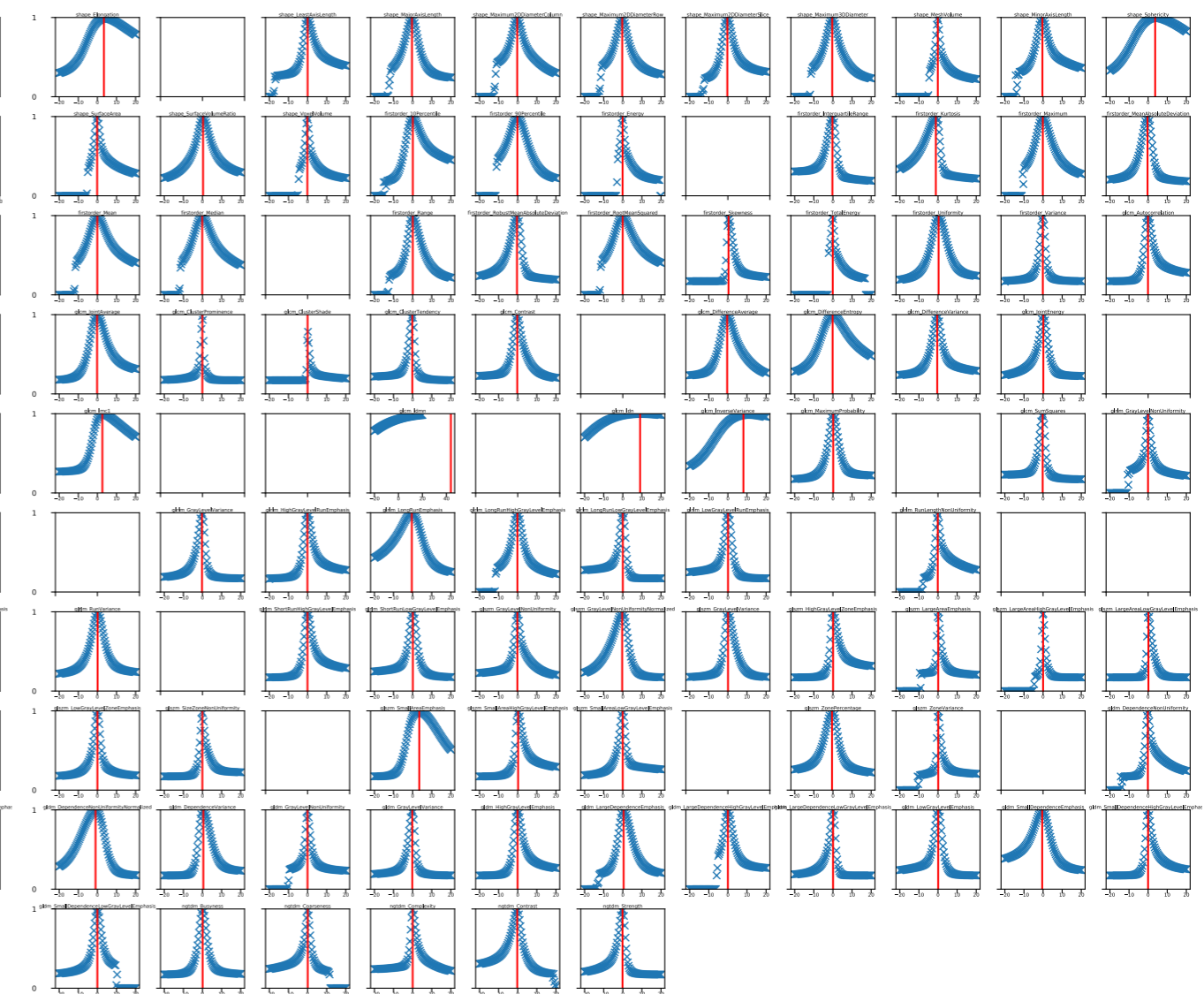

**(d)**

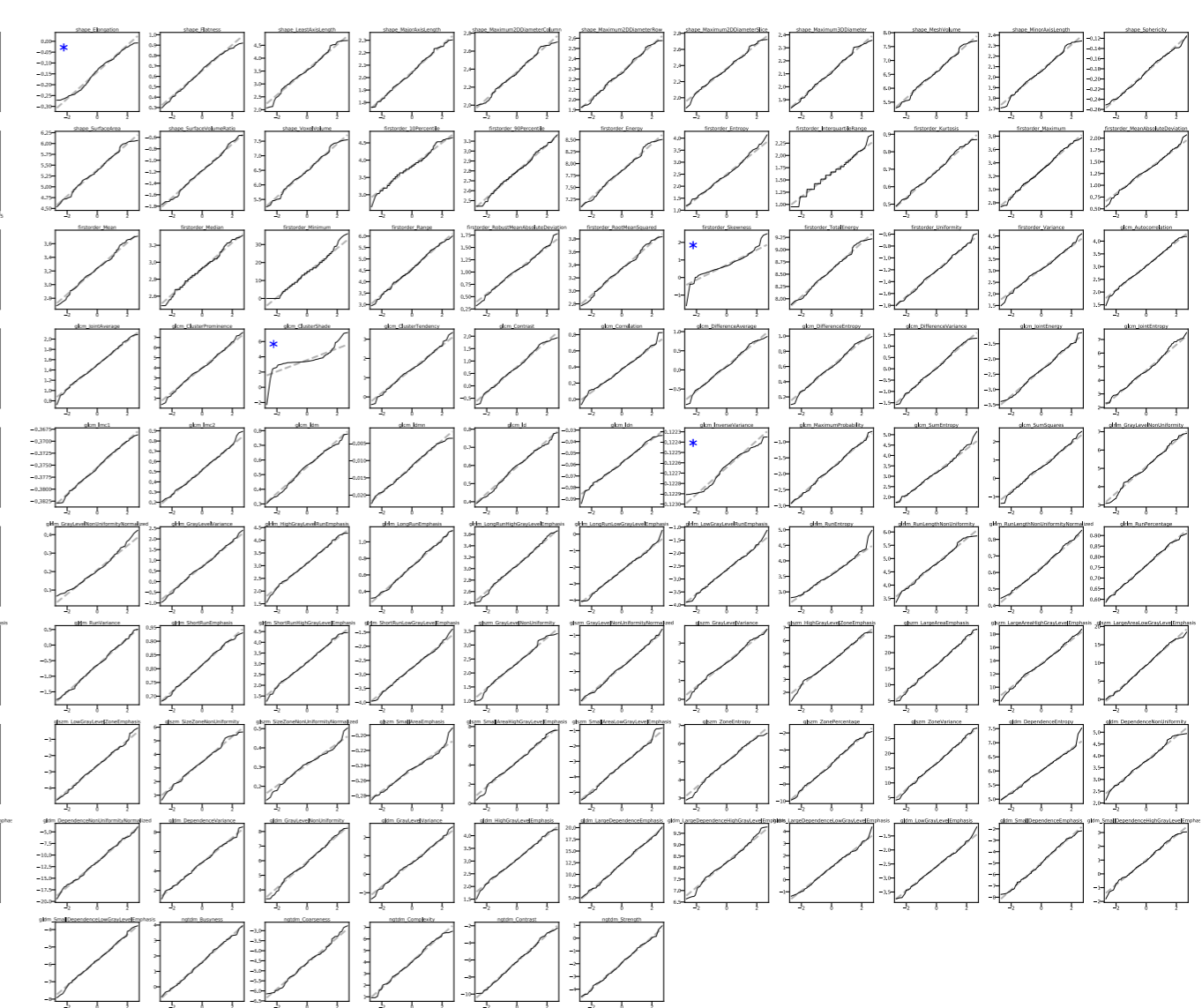

Supplement: Supplementary file 1 [file cancers-13-00240-s001.zip › MDPI_template_Cancers_radiomics_supporting_information_submission_071220/figures/supporting_feature_hists_box_cox_qq_t2w.pdf]

Δ transformed ICC  
(Original - Box-Cox)

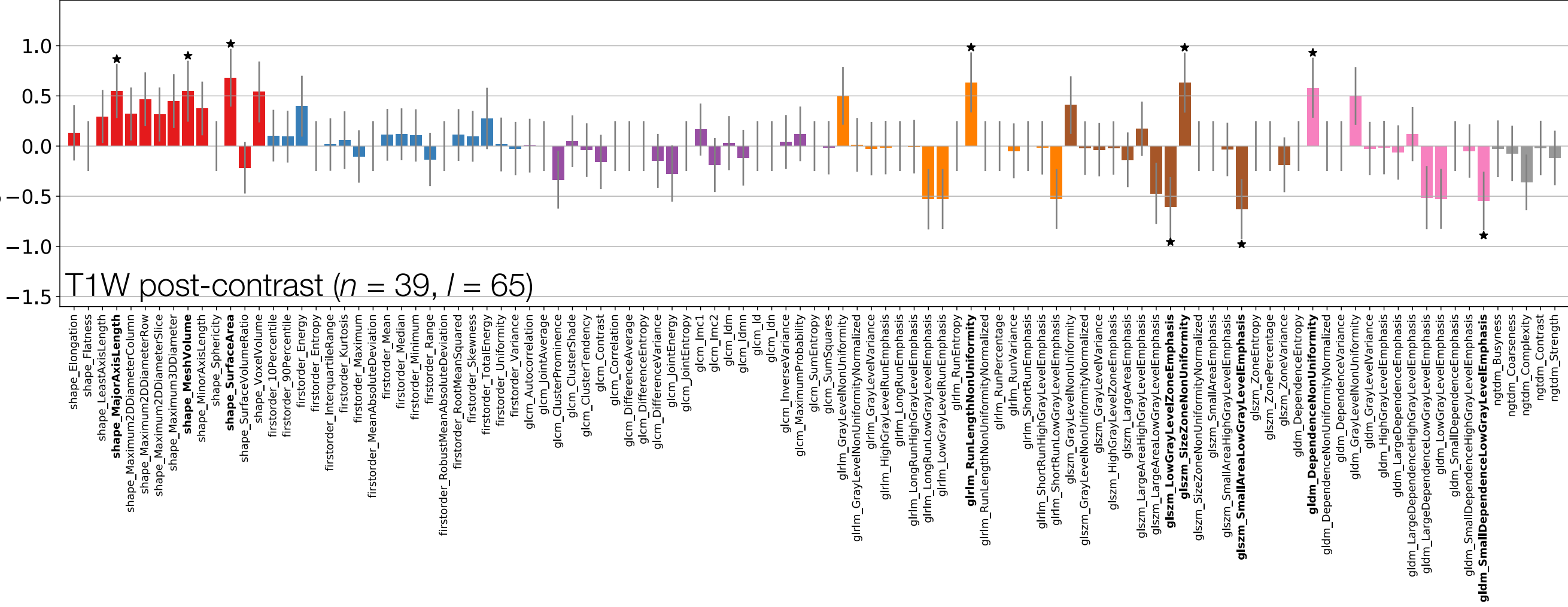

Δ transformed ICC  
(Original - Box-Cox)

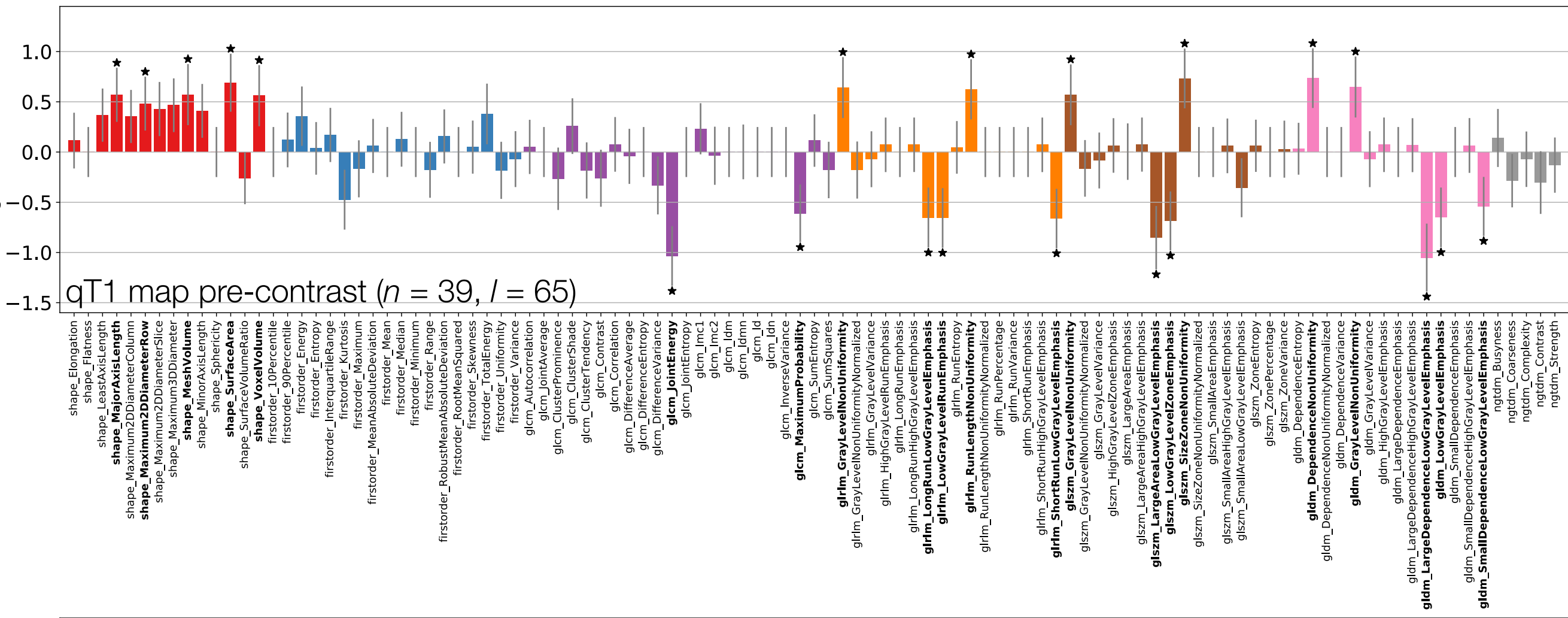

Supplement: Supplementary file 1 [file cancers-13-00240-s001.zip › MDPI_template_Cancers_radiomics_supporting_information_submission_071220/figures/supporting_feature_icc_orig_v_box_cox_qt1map_t1wpost.pdf]

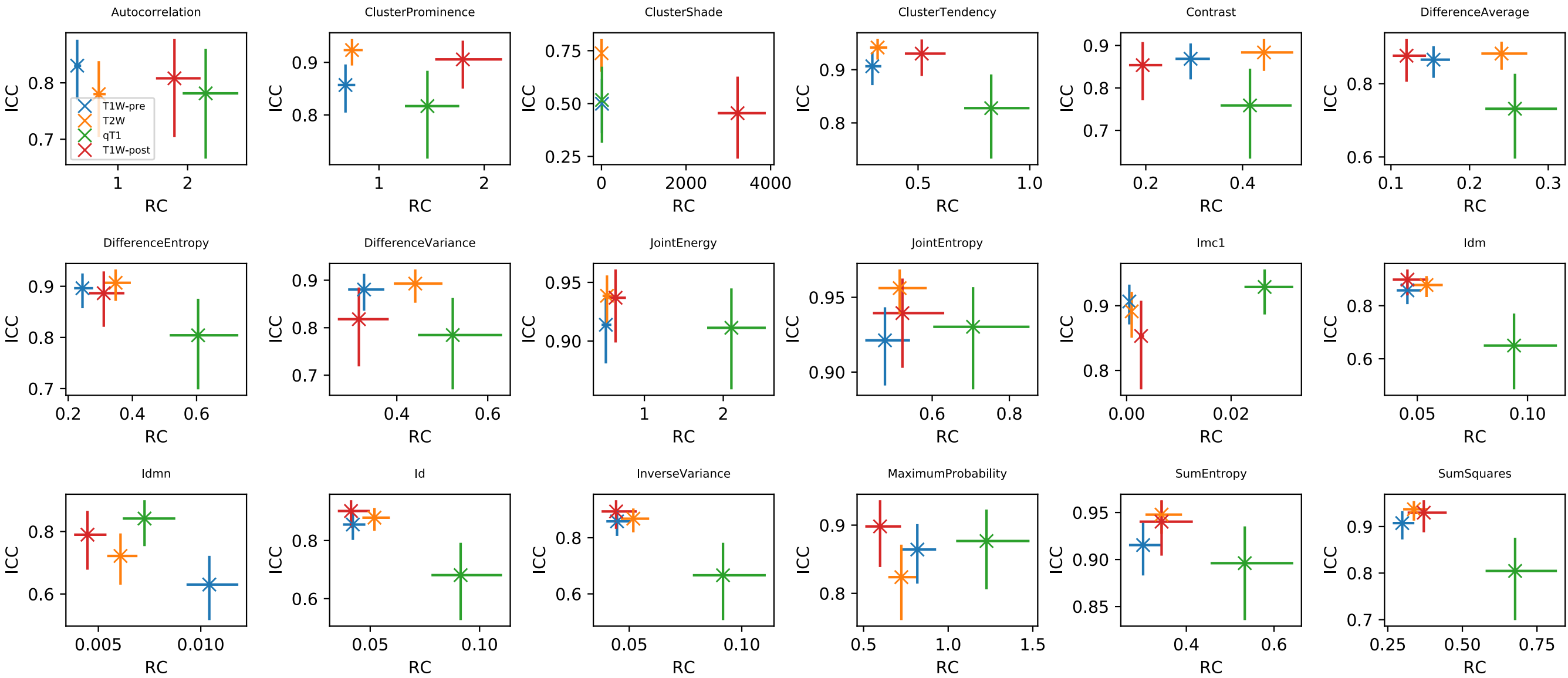

Supplement: Supplementary file 1 [file cancers-13-00240-s001.zip › MDPI_template_Cancers_radiomics_supporting_information_submission_071220/figures/supporting_cf_icc_rc_glcm.pdf]

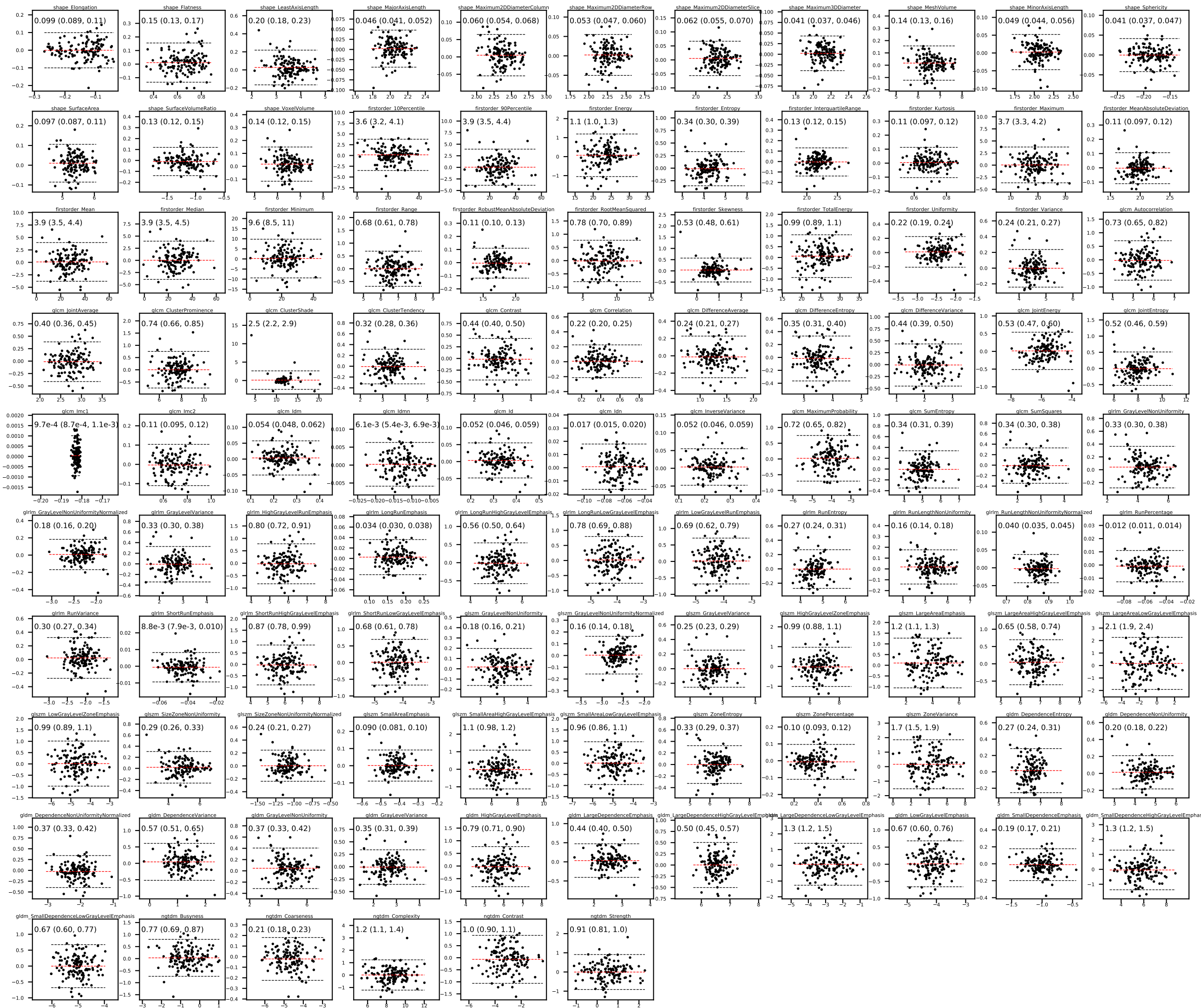

Supplement: Supplementary file 1 [file cancers-13-00240-s001.zip › MDPI_template_Cancers_radiomics_supporting_information_submission_071220/figures/supporting_bland-altman_BoxCox_105features_T2_Params_normalise_true_scale100_shift0_bw5.pdf]

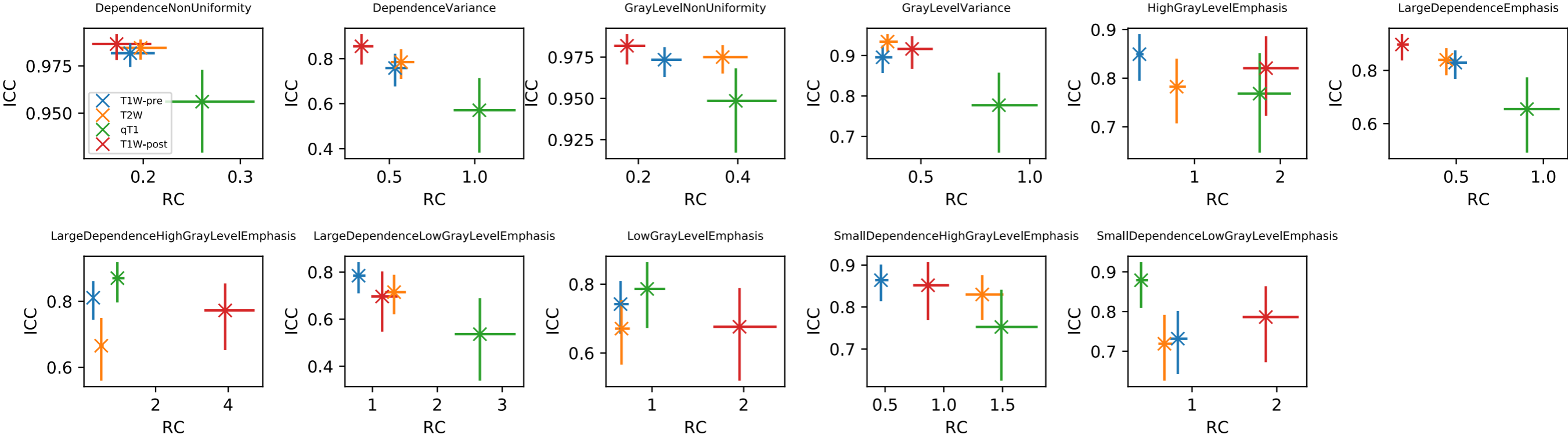

Supplement: Supplementary file 1 [file cancers-13-00240-s001.zip › MDPI_template_Cancers_radiomics_supporting_information_submission_071220/figures/supporting_cf_icc_rc_gldm.pdf]

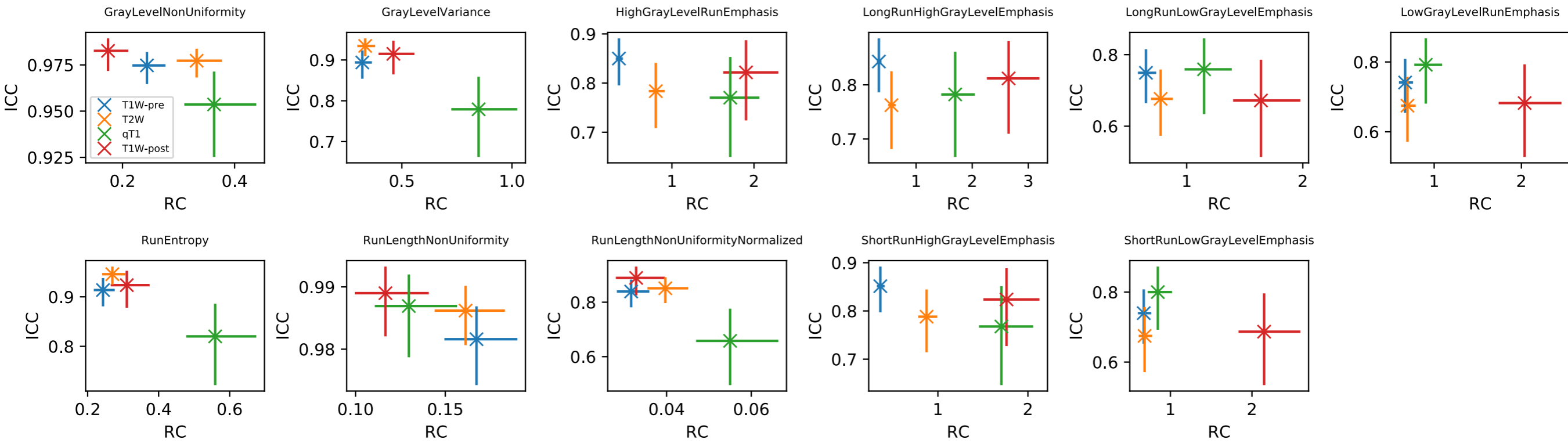

Supplement: Supplementary file 1 [file cancers-13-00240-s001.zip › MDPI_template_Cancers_radiomics_supporting_information_submission_071220/figures/supporting_cf_icc_rc_glrlm.pdf]

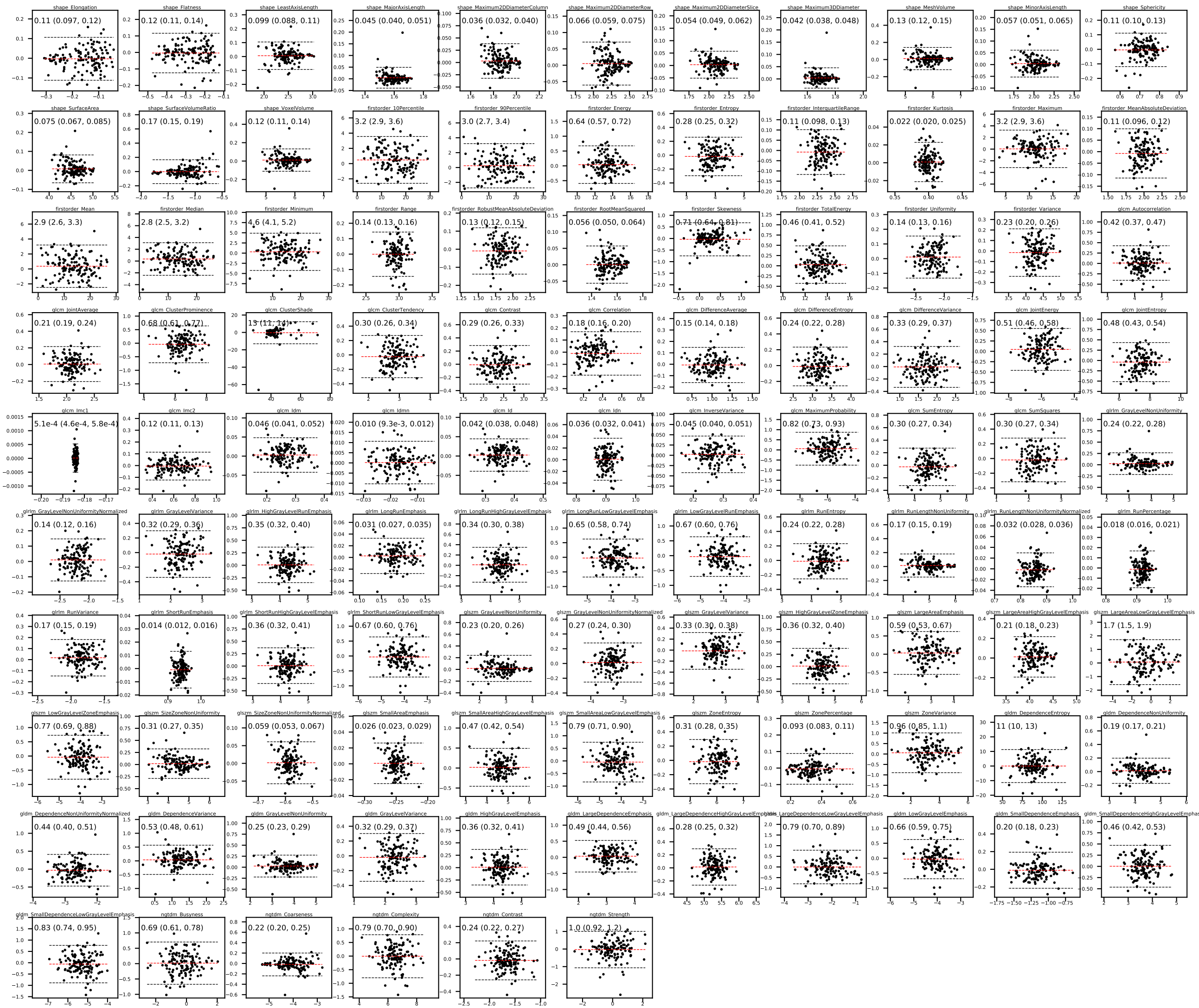

Supplement: Supplementary file 1 [file cancers-13-00240-s001.zip › MDPI_template_Cancers_radiomics_supporting_information_submission_071220/figures/supporting_bland-altman_BoxCox_105features_T1_precon_Params_normalise_true_scale100_shift0_bw5.pdf]

**(a)**

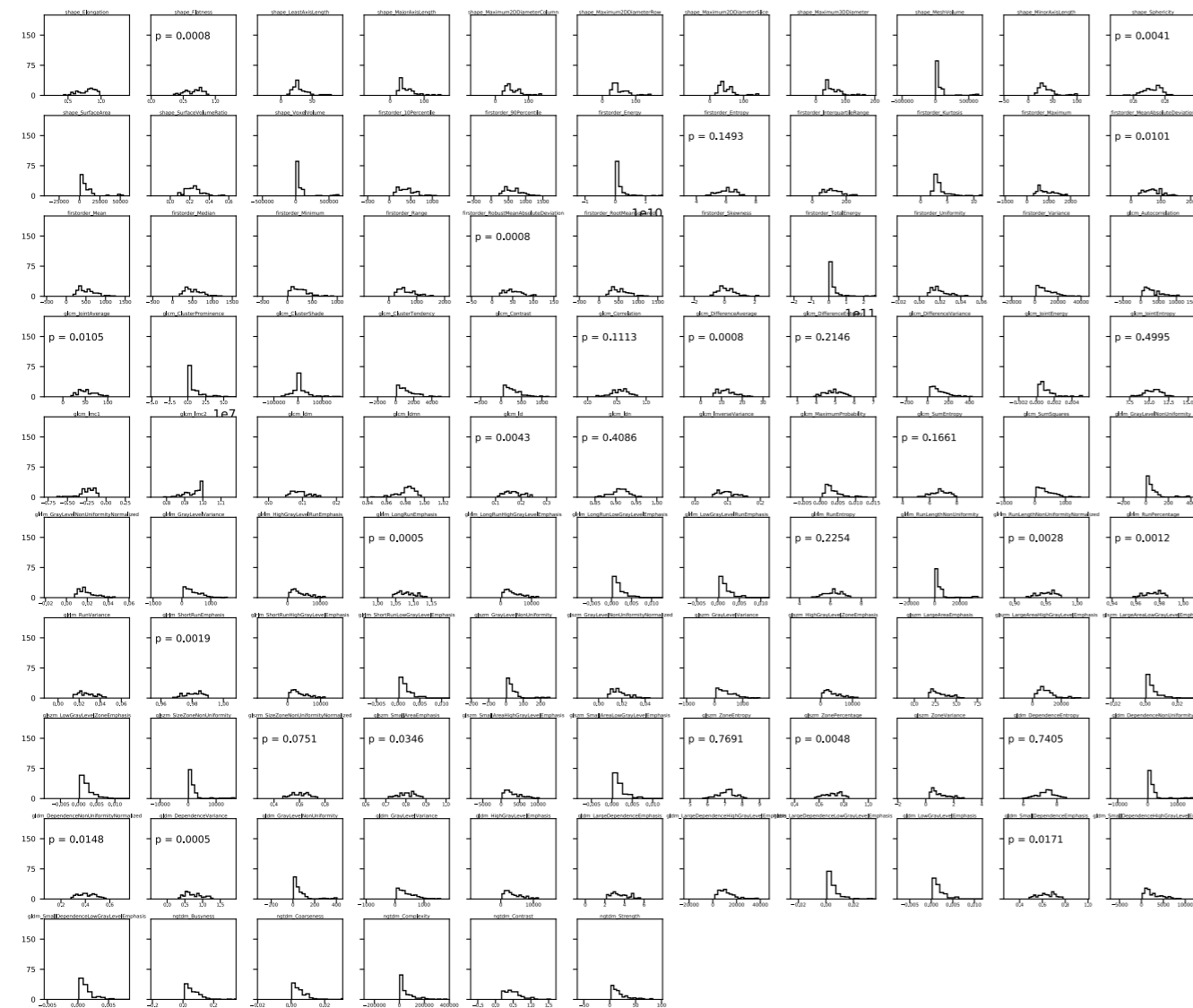

**(c)**

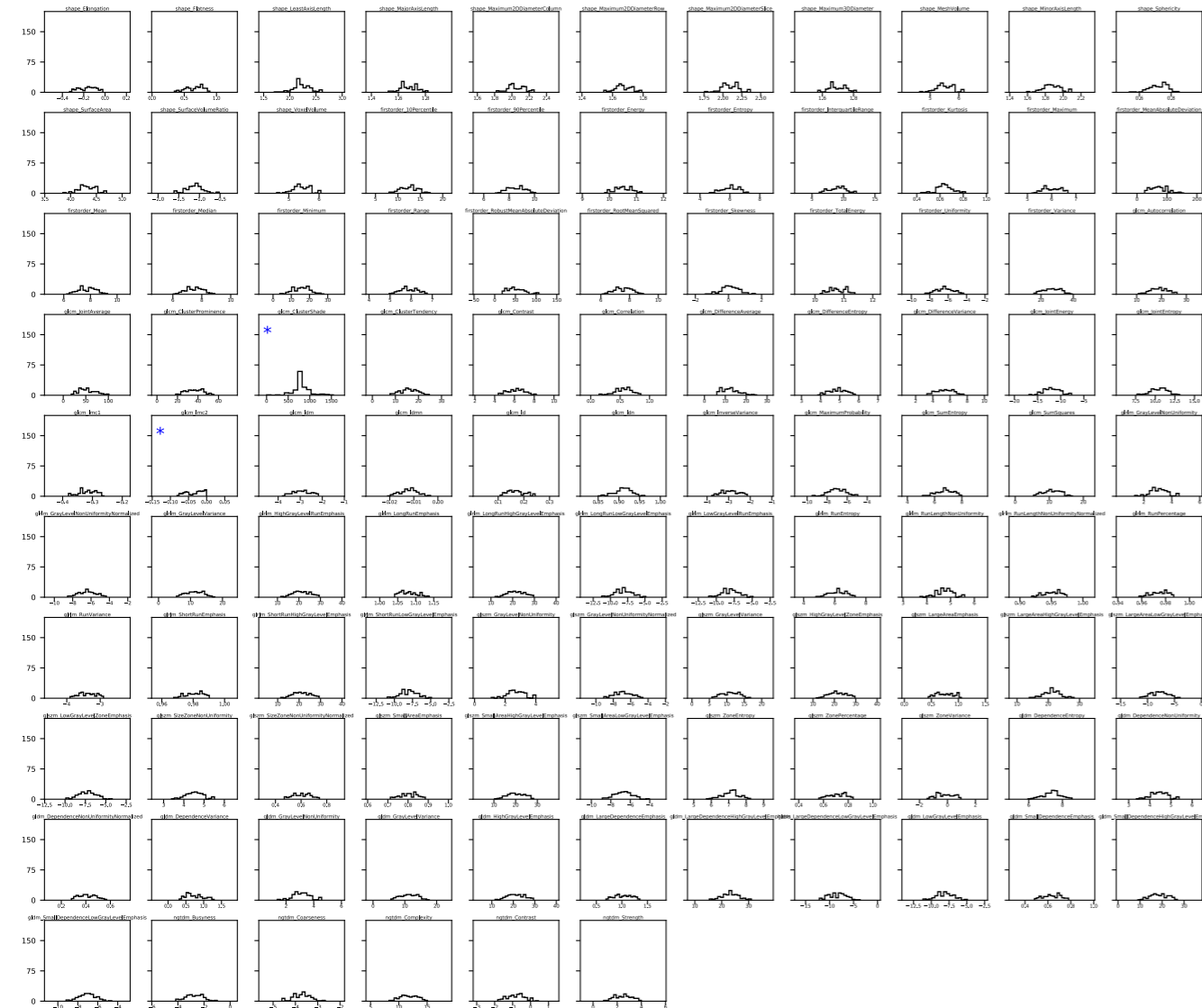

**(b)**

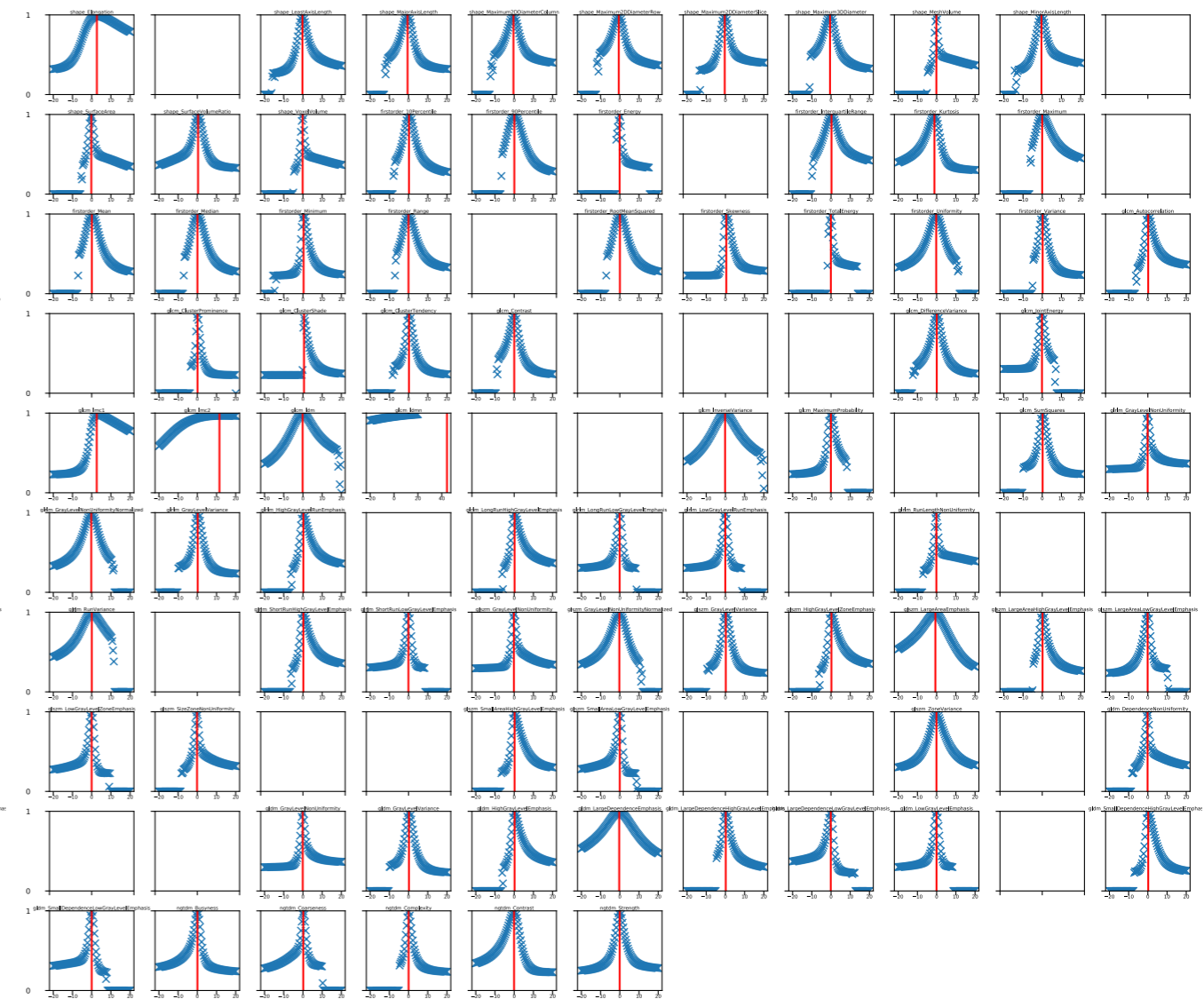

**(d)**

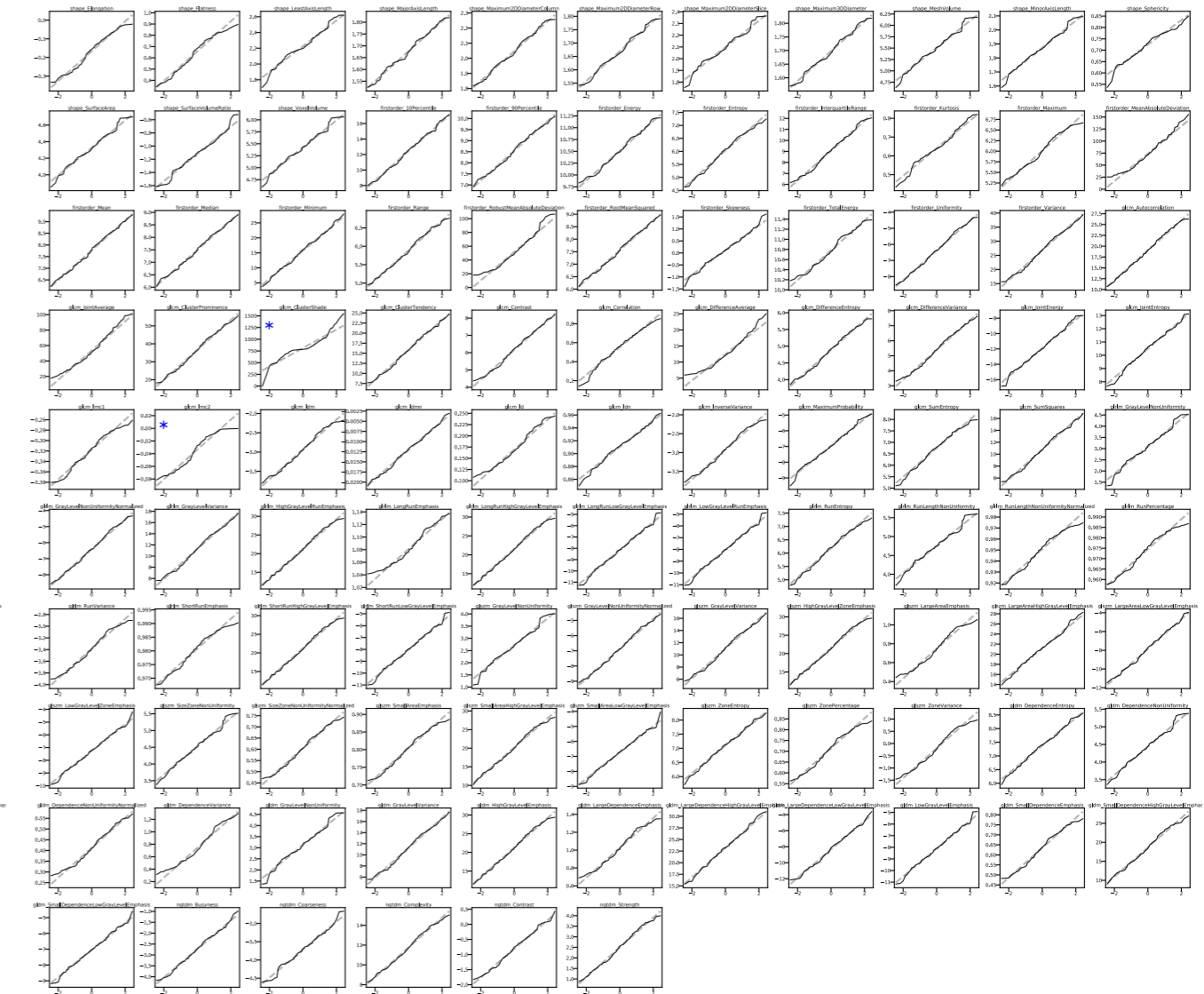

Supplement: Supplementary file 1 [file cancers-13-00240-s001.zip › MDPI_template_Cancers_radiomics_supporting_information_submission_071220/figures/supporting_feature_hists_box_cox_qq_t1wpost.pdf]

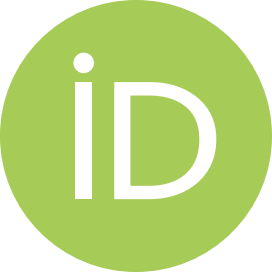

Supplement: Supplementary file 1 [file cancers-13-00240-s001.zip › MDPI_template_Cancers_radiomics_supporting_information_submission_071220/Definitions/logo-orcid-eps-converted-to.pdf]

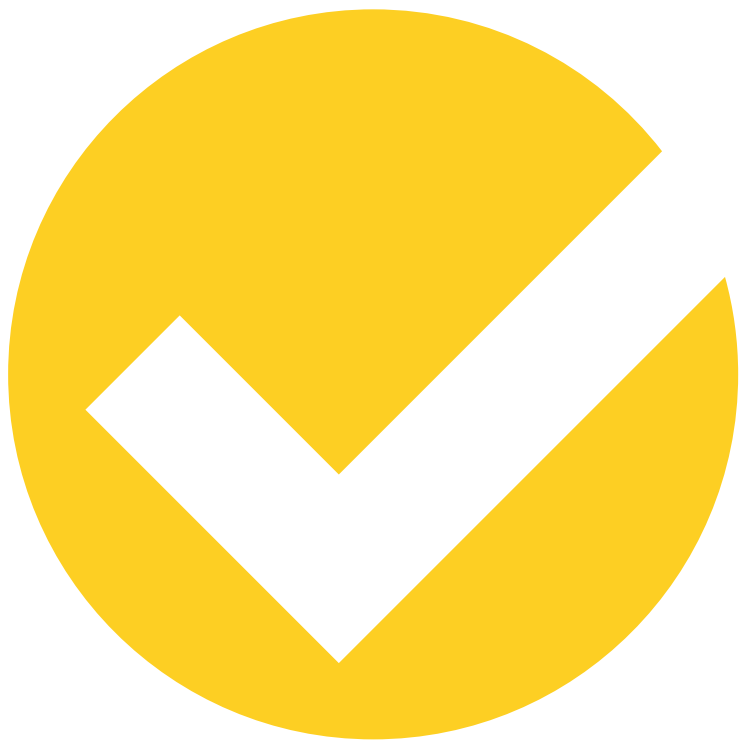

check for  
updates

Supplement: Supplementary file 1 [file cancers-13-00240-s001.zip › MDPI_template_Cancers_radiomics_supporting_information_submission_071220/Definitions/logo-updates.pdf]
